# Both L-Lactyl and D-Lactyl Enantiomers Modify Histones in Mouse Testis

**DOI:** 10.1016/j.mcpro.2026.101601

**Published:** 2026-06-12

**Authors:** Julie Manessier, Hassan Hijazi, Lisa Vizzini, Sabine Brugière, Marie Courçon, Christophe Masselon, Alberto de la Iglesia, Julie Cocquet, Delphine Pflieger

**Affiliations:** 1 University Grenoble Alpes, CEA, INSERM, UA13 BGE, CNRS, CEA, UAR 2048, Grenoble, France; 2 Institut Cochin, Université Paris Cité, INSERM, CNRS, Paris, France

**Keywords:** mouse testis, histones, lactylation, L-lactylation, D-lactylation, acetylation, targeted proteomic analysis, racemic mixture, lactate, methylglyoxal

## Abstract

Dynamic histone posttranslational modifications are crucial to precisely orchestrate gene expression programs. The recently discovered histone lysine lactylation has already been explored in various pathological contexts, but less in normal tissues. This modification exists as two enantiomers, L- and D-lactylation; the former may more likely modify histones due to abundant L-lactate produced by glycolysis. Here, we report the identification by proteomics of L- and D-lactylation on lysines of histones H3 and H4 in mouse testis. We developed a targeted proteomic analysis of histone peptides using synthetic sequences modified by L- or D-lactyl, to acquire reliable identification and quantification data. Some histone peptides bearing either enantiomer are separated by reversed-phase chromatography. Interestingly, despite the fact that L-lactate is much more abundant than D-lactate in mouse testis, we estimated abundance ratios of L-over D-lactylation to lie between 0.4 and 1.6 on seven residues of histones H3 and H4. Next, targeted proteomic analyses were performed on histones extracted from meiotic and postmeiotic male germ cells (spermatocytes and round spermatids, respectively), which are known to use L-lactate as a main source of energy. Nonetheless, residues 18 and 23 of histone H3 (H3K18 and H3K23) were reliably quantified and shown to harbor balanced amounts of both enantiomers. The stoichiometry of lactylation is low over the whole sequence of H3 and H4, representing about 0.01 to 0.44%: this contrasts with acetylation which exists at up to 25 to 35% relative abundances on some N-terminal lysines. Yet, lactylation appears to be more abundant than acetylation on the C-terminal half of H3 and H4, where the latter modification is scarce. Collectively, our results suggest a mechanism producing a mixture of the two enantiomers of lactate, or of a more direct substrate for lactylation, that leads to the modification of histones by L- and D-lactylation.

## Highlights

• L- and D-lactylation were identified on histones H3 and H4 in mouse testis.
• A targeted proteomic method was developed to distinguish lactylation enantiomers.
• L/D lactylation ratios are near parity despite excess L-lactate in mouse testis.
• Lactylation is of low stoichiometry but exceeds acetylation at H3 and H4 C-termini.

## Graphical Abstract

**Figure:**
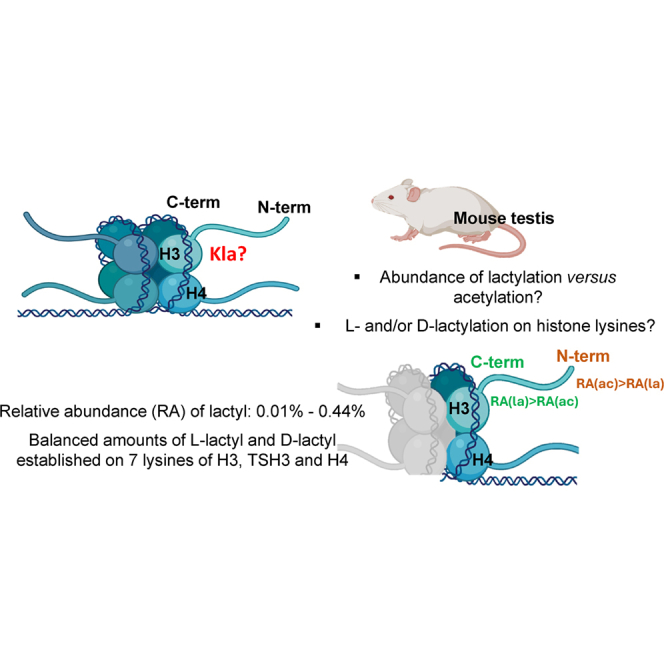

## In Brief

We report the proteomic identification and quantification of L- and D-lactylation on histones H3 and H4 in mouse testis. Using a targeted approach with synthetic modified peptides, we reliably distinguished both enantiomers and found similar levels at several residues, despite excess L-lactate. Although globally low in stoichiometry, lactylation exceeds acetylation on C-terminal regions. Our results encourage exploring new mechanisms to explain the addition of both L- and D-lactylation in histone lysines.

Gene expression is subtly regulated by the dynamics of histone posttranslational modifications (PTMs), the diversity of which has dramatically expanded with the successive discoveries of a variety of lysine acylations. These structures resemble acetylation but vary in length, hydrophobicity and charge, and are progressively described to be endowed with specific functions compared to the canonical PTM acetylation. These modifications are likely related to the corresponding acyl-CoA metabolites, which establishes an elaborate link between metabolism and epigenetics regulatory mechanisms (1, 2). In 2019, lactylation was discovered to regulate gene expression in macrophages challenged by bacteria and hypoxia (3). It was described to be induced by L-lactate, a metabolite produced by glycolysis *via* the enzyme lactate dehydrogenase A, which can be very abundant in cell types heavily relying on this energy producing cycle, such as cancer cells (4). Yet, further publications supported two scenarios to explain the addition of lactylation onto histones, and beyond, to other proteins (5). The first one is enzymatic and uses L-lactate as indirect substrate (3), whereas the second one hypothesizes a nonenzymatic addition of lactylation from D-lactoyl-glutathione (6).

Indeed, on the one hand, several studies described the transfer of a lactyl moiety to lysines by enzymes of the histone acetyltransferase (HAT) families, or, more astonishingly, *via* other metabolic enzymes. In the original description of histone lactylation, Zhang *et al.* demonstrated that lactyl-CoA could serve as the substrate for histone lactylation in chromatin template-based histone modification and transcription assays *in vitro,* involving p300 as the lactyltransferase (3). Whether this mechanism holds true *in vivo* remains to be studied, due to the very low abundance of lactyl-CoA in mammalian tissues (7). Among the enzymes recently described to be involved in lysine lactylation are AARS1 and 2 (8, 9, 10), which couple lactate with AMP to form a reactive substrate. Besides, HBO1, a member of the MYST family of HATs, was demonstrated to catalyze histone lactylation, particularly at lysine 9 from histone H3 (H3K9), and of other proteins in HeLa cells (11). Other described mechanisms of lactylation involve ACSS2 associated with KAT2A (12), and guanosine triphosphate-specific succinyl-CoA synthetase in complex with p300 (13). The overall mechanisms leading to enzymatic histone lactylation and in particular the enzymes responsible for producing the substrate for lactylation (lactyl-CoA or lactate-AMP) and its transfer to histone and nonhistone lysine residues may very well depend on the cell type considered, which determines the cellular abundance of these enzymes (13).

On the other hand, Gaffney *et al.* described in 2020 the nonenzymatic addition of D-lactyl, mostly on nonhistone proteins, D-lactoylglutathione (LGSH) being the substrate for this modification (6). The two major enzymes involved in regulating this PTM are GLO1 and GLO2. The same researchers further demonstrated *in vitro* that lactyl-CoA led more readily to histone lactylation than LGSH, but that LGSH efficiently reacted with CoA to generate lactyl-CoA, thus constituting an important indirect source of nonenzymatic protein lactylation (14).

Many of the above studies describing L-lactylation were performed on cancer cells and tumors, characterized by remarkably high intracellular levels of L-lactate derived from exacerbated aerobic glycolysis, a phenomenon called the Warburg effect (13). Whether L- or D-lactate actually contributes to histone lysine lactylation in other cellular contexts is still a matter of debate. This question is worth investigating under physiological conditions, that is, in healthy tissues. Another important aspect to investigate is the relative abundance of lactylation compared to acetylation. To the best of our knowledge, this quantity has never been assessed in reports on histone lactylation.

In the present work, we carefully dissected the lactylation patterns of histones H3 and H4 extracted from mouse testis. This organ constitutes an interesting model to study histone PTMs, because the differentiation of male germ cells into spermatozoa is characterized by dramatic chromatin remodeling and waves of histone PTM changes, including acetylation (15), butyrylation (16) and crotonylation (1). We studied in particular the latter PTM on histone H3 lysine 27 (H3K27) and its association with H3K27ac to induce transcription (17). Since lactate is the predominant energy source of differentiating male germ cells (18), we sought to characterize histone L- and D-lactylation in the testis. Interestingly, a couple of recent studies have investigated histone lactylation in this organ (19, 20). Both studies exclusively relied on antibody-based experiments targeting selected N-terminal lysines of H3 and H4. Here, by quantitative proteomics using heavy labeled synthetic peptides modified with L- or D-lactylation, we demonstrate that the two enantiomers coexist on several lysine residues from H3 and H4, in protruding N-terminal ends as well as globular regions. We also estimate that the relative stoichiometry of lactylation is constantly low, ranging between 0.01 and 0.44%, over the whole sequence of both histones. This is in stark contrast with the stoichiometries of acetylation, which can be very abundant on the N-terminal tails (*e.g*. between 22% and 35% at H3K14, H3K23 and H4K16), but appear to be of lower abundance than lactylation at H3K56, H3K64, H3K79, H3K122 and H4K77. In all, our findings prompt a reconsideration of the mechanisms by which histone lysines become lactylated, to account for the addition of both enantiomers in healthy tissues.

## Experimental Procedures

### Materials

Mouse testes were obtained from adult (2–3 months old) C57BL/6 males. Procedures were approved by local ethical review (Comité d'Ethique pour l'Experimentation Animale, Université Paris Descartes, registration number CEEA34.JC.114.12, APAFIS 14214–2017072510448522v26). Synthetic peptides were purchased from JPT Peptide Technologies, in SpikeTides_L quality; their sequences and modifications are listed in Supplemental Table S1. Triethylamine bicarbonate (TEAB) (ref. T7408), sulfuric acid (ref. 339741), tricholoroacetic acid (TCA, ref. T0699), propionic anhydride (ref. 240311), hydroxylamine (ref. 438,227) and NH_4_OH (ref. 221228) were purchased from Sigma.

### Acid Extraction of Histones from Mouse Testes and from Purified Spermatocytes and Round Spermatids

Histones were extracted from mouse testicular nuclei using the following protocol, derived from (21). Briefly, each testis was homogenized in 1 ml of Nuclei Isolation Buffer (NIB; 15 mM Tris-HCl (pH 7.5), 60 mM KCl, 11 mM CaCl2, 5 mM MgCl2, 250 mM sucrose, 1 mM DTT, 10 mM sodium butyrate, 3 μM trichostatin A, 50 mM nicotinamide, complete mini protease inhibitor cocktail from Roche, and 0.3% final (v/v) NP-40) using a Dounce homogenizer. Lysates containing nuclei were centrifuged at 1000g, 4 °C for 5 min. The pelleted nuclei were then resuspended in NIB without NP-40 and centrifuged at 1000g, 4 °C for 5 min. This wash was repeated a second time. The nuclei were resuspended in 450 μl of 0.2 M H_2_SO_4_ and rotated at 4 °C for 1 h, then centrifuged at 4 °C at maximum speed for 5 min. Histone-containing supernatants were collected in new tubes and precipitated by adding pure TCA (final concentration 20%) and incubating for 2 h in ice, then centrifuged at 20,000*g*, 4 °C for 15 min. The supernatant was aspirated and the precipitated histones were coated with 0.1% HCl in freezer-cold acetone, then centrifuged at 4 °C for 5 min at maximum speed. This step was repeated once with nonacidified acetone. After removing the acetone, samples were dried under the hood. Finally, histone samples were resuspended in 100 μl of Milli-Q water and stored at −20 °C. An aliquot of histones was separated by SDS-PAGE (4–12%) to verify their purity and estimate the total amount (approximately 200 μg of histones were extracted per testis). For the gel-based analysis, the bands corresponding to histones H3 and H4 were cut with a scalpel and stored at −20 °C until being processed.

Histones were extracted from whole spermatocytes (SC) and round spermatids (RS) that had been collected from mouse testis by elutriation. Between 2 and 2.8 million SC and 5 million RS were used to enrich for histones as described in (17). About one third of each sample was loaded on a 4 to 12% acrylamide gel to separate histones from higher-molecular-weight proteins. Slices containing H3 to H4 were cut to be subjected to the propionylation-trypsin digestion protocol described below.

### *In vitro* Propionylation and Tryptic Digestion of Histones

For each step of primary amine derivatization by propionyl, two propionic anhydride solutions were extemporaneously prepared as follows: a first dilution of pure propionic anhydride diluted at 1:100 in water, followed by a second dilution of this solution in 1 ml of 100 mM TEAB (pH 8.5). In-gel and in-solution derivatization procedures shared most steps. Histone-containing gel pieces were first destained. For derivatization, 100 μl of propionic anhydride solution diluted in TEAB were added to each gel band for 30 min. This step was repeated a second time, after removing the first solution and drying the gel pieces in pure acetonitrile. Histones in solution were propionylated in two rounds as above, separated by a drying of samples in a SpeedVac. In both approaches, histones were then digested with trypsin diluted in 50 mM TEAB overnight at 37 °C, at an enzyme:substrate mass ratio of 1:20. After digestion, peptides were extracted from the gel pieces. Another propionylation step was performed to label the N-terminal ends of peptides, as previously performed on intact histones. To remove nonspecific propionylated sites (typically at Ser/Thr residues), a reverse propionylation step was carried out by applying 20 μl of 500 mM hydroxylamine and 6 μl of ammonium hydroxide (pH 12) for 20 min at RT with stirring (600 rpm) (22, 23, 24). The reaction was stopped by adding a few drops of pure TFA. Samples were dried in a SpeedVac and desalted using Affinisep SPE cartridges (BioSPE PurePrep).

### Biochemical Processing and LC-MS/MS Analyses of Synthetic Peptides

The synthetic heavy peptides modified by either L-lactylation, D-lactylation or acetylation were verified by LC-MS/MS analysis in terms of correct sequence, modification site, and absence of light counterparts. To test the chromatographic behavior of D- and L-lactylated peptides, three different mixtures of these synthetic peptides were prepared, in approximate molar ratios 1:1, 1:2, and 2:1, respectively. These samples were propionylated as described above, followed by a reverse propionylation step. The peptides were then desalted and analyzed by LC-MS/MS on a C18 Aurora column. To assess the presence of L- and then also D-lactylation in mouse testis histones, endogenous samples were successively spiked with two mixtures of synthetic peptides. A first mixture was made up of all L-lactylated and acetylated peptides and a second mixture of L-lactylated, acetylated, and D-lactylated peptides. About 0.5 μg of endogenous histone peptides and 50 fmol of each synthetic peptide were injected each time.

### Liquid Chromatography-Mass Spectrometry Analyses

Peptides were loaded onto a PepMap C18 precolumn (300 μm × 5 mm, Thermo Fisher Scientific) with 0.1% formic acid. The peptides were then separated on a reverse-phase capillary column (Aurora C18, 75 μm × 25 cm, 1.7 μm beads, 120 Å particles) on a Nano Ultimate 3000 system (Thermo Fisher Scientific) coupled to an Exactive HF mass spectrometer (Thermo Fisher Scientific). The mobile phases consisted of solvent A (water with 0.1% formic acid) and solvent B (acetonitrile with 0.08% (v/v) formic acid). The peptides were separated by applying a gradient consisting of an increase from 2% to 7% of B in 5 min, then 7% to 31% of B in 55 min, then 31% to 41% B in 8 min, followed by a column wash at 72% B for 9 min and column reequilibration at 2% B for 14 min.

For analyses in data-dependent acquisition (DDA) mode, MS1 spectra were acquired at a resolution of 60,000 with an AGC of 1e6, over an m/z range from 300 to 1300. MS2 spectra were acquired at a resolution of 15,000 with an AGC of 2e5, with a maximum injection time of 100 ms, a loop count of 20 and an isolation window of 1.5. Peptides were isolated for fragmentation using higher-energy collisional dissociation with a collision energy of 30 and a dynamic exclusion of 10 s. The first fixed mass of MS/MS spectra was set at m/z 80, to be able to detect the cyclic immonium (CycIm) ion produced by monomethylated lysines (at m/z 98.09). For analyses by parallel reaction monitoring (PRM), MS2 spectra were acquired at a resolution of 30,000, with an AGC of 1e6, a maximum injection time of 120 ms and an isolation window of 1.6.

Scheduled PRM analyses were designed to target (i) L-lactylated and acetylated peptides to start studying the presence of L-lactylation in mouse testis histones (Supplemental Table S2), (ii) L-, D-lactylated and acetylated peptides to determine the abundance ratio of L-*versus* D-lactylation (L/D), as well as of acetylation *versus* lactylation (ac/la) (Supplemental Table S3). In the latter case, we included the targeting of oxidized forms of methionine-containing peptides H3 VTIMPK122DIQLAR and H4 K79TVTAMDVVYALK91R. Finally, to be able to get an estimate of the stoichiometry of L- and/or D-lactylation as a percentage of each H3 and H4 lysine residue, we determined the stoichiometry of each corresponding acetylation site. DDA analyses were relevant to estimate these values for N-terminal lysines where this PTM is quite abundant (Supplemental Table S4), whereas PRM analyses were designed to systematically characterize lysines from the histone fold domain (Supplemental Table S5).

### Preprocessing, Identification, and Quantification of Modified Peptides from DDA and PRM Analyses

For DDA analyses, the acquired RAW data were converted to.MGF files by the in-house developed tool MGFBoost (article in preparation; https://www.profiproteomics.fr/proline/other-tools/), which maintains the relative intensities of fragments in MS/MS spectra (23). Identification of histone peptides was obtained with the Mascot search engine (Matrix Science v2.8) using an in-house histone database complemented with a list of classical contaminants (25). The search parameters were as follows: Arg-C as the enzyme with no missed cleavage, Propionyl (N-term) as a fixed peptide modification, acetyl (K), butyryl (K) standing for the dual modification by endogenous monomethyl and chemical propionyl, dimethyl (K), trimethyl (K), lactyl (K), propionyl (K), oxidation (Q), and oxidation (M) as variable modifications. Peptide mass tolerance was set at 5 ppm and fragment mass tolerance at 20 ppm. Filtering identification results in terms of false discovery rate at peptide level is not relevant due to the small sequence database explored. We considered peptide identifications with a minimum Mascot score of 25 and visually inspected interpreted MS/MS spectra, by checking (i) that peptides of same sequence and same charge but bearing different PTMs shared similar fragmentation patterns, and (ii) intense CycIm ions were detected for N-terminal lysines modified by me1/ac/pr/la. Estimation of the relative abundance of acetylation sites on the N-terminal half of H3 and H4 was obtained by processing DDA analyses in proline (26), while applying the match-between-runs option to get quantification of all peptides identified in at least one of the four biological replicates analyzed. For PRM analyses, .RAW files were imported into Skyline software v23.1 and lactylated or acetylated peptides of interest were quantified, based on the presence of heavy-labeled synthetic sequences. Each peptide identification was visually inspected in Mascot, in particular to verify the presence of intense CyIm ions corresponding to the modified lysine in first position (23). Peptide quantification was calculated from PRM analyses based on specific y-type fragments indicated in Supplemental Tables S3 and S5, while considering the area under the curve.

### Experimental Design and Statistical Rationale

Samples of synthetic D- and L-lactylated peptides prepared in 1:1, 1:2, and 2:1 ratios were analyzed by LC-MS/MS in technical duplicates which are both shown in Supplemental Fig. section S3. Histones from mouse testes were obtained from four mice. These four biological replicates were analyzed to estimate the abundance ratio of L-*versus* D-lactylation, as well as acetylation. Of note, when estimating the abundance ratios of L-*versus* D-lactyl and lactyl *versus* acetyl at the same lysine sites, we compared mass spectrometry signals within a single PRM analysis, so that normalization of total histone amounts between samples was not necessary. We, however, had to normalize the raw L- and D-lactyl amounts estimated from MS2-fragments to determine whether the L/D lactyl ratios at lysine residues whose enantiomers were chromatographically separated were significantly different from 1 or not. The MS2 signals quantified for these peptides are provided in Table S10 and the different tests performed using the program JMP are detailed in Supplementary fig. section S4. Among the various types of targeted proteomic analyses, this work corresponds to Tier 3. Indeed, our PRM analyses aimed to get an estimate of the relative abundance of lactylation at various lysines of histones H3 and H4, by label-free quantification, using the heavy-labeled synthetic peptides as a reference to correctly integrate the signal for lactylated species. For peptides identified in multiple charge states by DDA, we selected the doubly charged ones, for both lactylated and acetylated peptides of same sequence. We observed that the ratio dot product, rdotp, which measures similarity between the endogenous peptide and its isotopically labeled version, had a median value of 0.93 for lactylated peptides over all runs aiming to estimate the relative abundance of L- and D-lactylated species. Furthermore, we verified that all peptides bearing a lactylation in first position exhibited an intense CyIm ion at m/z 156.1019. However, this fragment ion was not taken into account for quantification, because its contribution to the MS2 signal would have introduced a discrepancy between positional isomers, being modified on the N-terminal lysine or inside the sequence. Importantly, the fact that the tentatively lactylated endogenous peptide and the definitely lactylated synthetic peptide possessed the exact same retention time constituted a critical validation criterion: slightly differing histone peptides indeed shared numerous transitions. For relative quantification, one to foury fragments of m/z values above that of the precursor ion were chosen, that usually represented >80% of the summed area of all y fragments (Supplemental Tables S3 and S5). The sole exception was the N-terminal peptide of histone H4, which contains four lysines H4K5, K8, K12, and K16, and for which three positional isomers coeluted. For this peptide, we only followed the intense y5 fragment, which allowed discriminating between acetylation or lactylation at either H4K5/K8/K12 or at H4K16.

PRM analyses were used to estimate ac/la abundance ratios. Even though we considered the same y fragments from the same charge state of each peptide as being either lactylated or acetylated, ionization efficiencies may differ between peptidoforms bearing each modification. Yet, these two PTMs are chemically similar, as shown by their very close retention times (Supplemental Fig. section S7). This chemical proximity makes the calculation of ac/la abundance ratios relevant. To estimate the relative abundance of lactylation sites (RA(la)), we estimated the stoichiometry of the corresponding acetylation moiety (RA(ac)), and used the following equation:

(1) $$RA\left(la\right)\;=\;\frac{RA\left(ac\right)}{\left[\frac{ac}{la}ratio\right]}$$

For abundant acetylation sites on the N-terminal tails of H3 and H4, we processed DDA analyses using the program Proline (26) which quantifies the MS1 intensity of all identified peptides. The RA of each acetylated peptide was calculated as follows:

(2) $$RA\left(peptide\left(i\right)\_ac\right)\;=\;\frac{MS1\;intensity\;\left(peptide\left(i\right)\_ac\right)}{\Sigma MS1\;intensities\;of\;all\;modified\;forms\;of\;peptide\left(i\right)}$$

We acknowledge that this calculation may be biased by differences in ionization efficiencies of acylated *versus* methylated peptides, as previously reported in (27, 28). For very low-abundance acetylation sites in the histone-fold domain of H3 and H4, we resorted to PRM analyses. Except for H3K79 that exists in monomethylated and dimethylated forms at high stoichiometries, the peptides containing the lysines of the C-terminal half are mostly nonmodified (see Supplemental Table S5 for the list of considered peptidoforms containing these lysine residues). So, in most cases, the PRM signal of the acetylated peptide was compared to that of the propionylated one using Equation (2).

### Dosage of L- and D-Lactate in Mouse Testes by Colorimetric Assays

Four mouse testes (between 110 and 123 mg fresh tissue) from four three-month-old mice were used to estimate the abundance of both enantiomers, L- and D-lactate. Kit ab65331 for dosing L-lactate and kit ab83429 for dosing D-lactate were purchased from Abcam. Each testis was lysed in 1 ml of Assay Buffer 12 provided in the kits, using a Dounce homogenizer. The resulting lysate was centrifuged at maximum speed for 5 min and 900 μl of supernatant were transferred to a new tube. Proteins were precipitated by adding 90 μl of TCA, incubating on ice for 5 min, and centrifuging at maximum speed for 5 min 800 μl of supernatant were collected and transferred to a fresh tube, to which 126 μl of 4M KOH were added, to reach a pH around 6.5 to 7. Then, the enzymatic assays were performed following the instructions of the Abcam protocols, by depositing in a 96-well plate 50 μl out of the total volume of 926 μl that represented around 73% of each total testis extract. That is, the extract of about 4% of testis was submitted to dosage of L- or D-lactate.

## Results

### Identification by Proteomics of Probable Lactylation Sites on Histones H3 and H4 from Mouse Testis

**Fig. 1:**
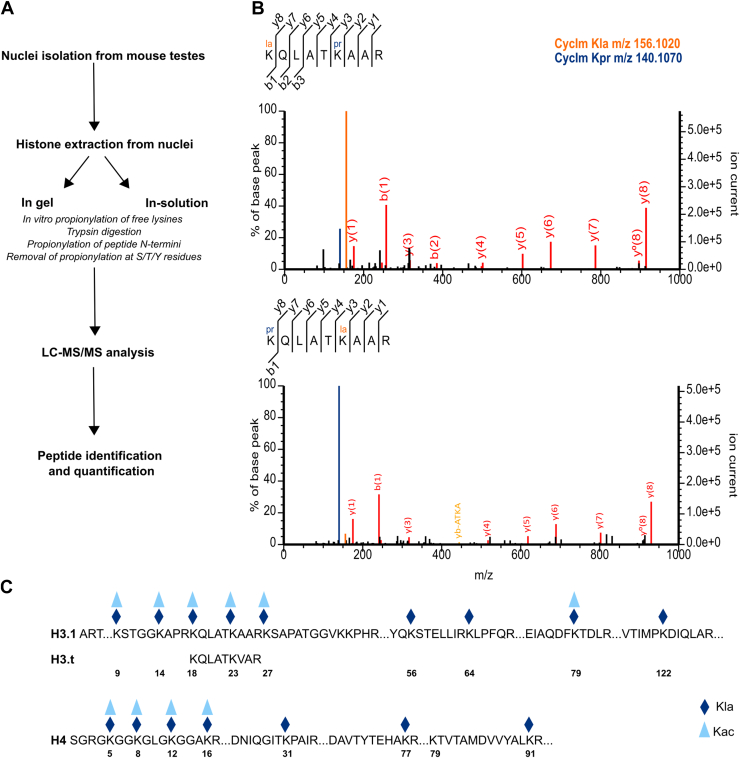
**Establishment of a mapping of lactylation sites on histones H3 and H4 from mouse testis.***A*, workflow followed to identify lactylation sites by DDA analysis. *B*, validation of the presence of a lactylation (mass increment of 72.021 u) at lysines H3K18 and H3K23, by detection of the cyclic immonium ion expected for Kla at m/z 156.1019 and of discriminating y fragments. *C*, global mapping of lactylation and acetylation sites over the sequences of H3 and H4. DDA, data-dependent acquisition.

We sought to obtain a global mapping of tentative lactylation sites on histones H3 and H4 from mouse testis, by following the workflow depicted in Figure 1*A*. After enrichment of nuclei from tissues, histones were acid-extracted and then either separated by SDS-PAGE (see Supplemental Fig. section S1 for a representative gel obtained) or directly processed in solution by propionylation of endogenously nonmodified lysines, trypsin digestion, treatment by a second propionylation of peptide N-termini, and finally with a basic solution to remove excess propionylation at S/T/Y residues (22, 24). Mascot processing of the liquid chromatography-tandem mass spectrometry (LC-MS/MS) data acquired on these samples yielded several peptide identifications bearing a precise 72.021 u mass increment, which may correspond to lactylation (Supplemental Tables S6 and S7). We carefully scrutinized each MS/MS spectrum matched to a lactylated peptide from histones H3 and H4, by verifying the following criteria. If the lactylation site was attributed to the lysine in first position of the sequence, we required the b1 fragment and the CycIm ion corresponding to lactylated lysine (Kla) to be detected with high intensity, as formerly reported (23, 29, 30, 31) (Fig. 1*B*). If lactylation was attributed to a lysine residue inside the peptide sequence, we verified the presence of y fragments directly surrounding the Kla site (Fig. 1*B*). We finally established a global mapping of probable lactylation sites on histones H3 and H4, and compared it to the acetylation sites identified from the same LC-MS/MS analyses (Fig. 1*C* and MS/MS spectra in Supplemental Fig. section S1). Interestingly, lactylation sites were detected on H3K56, H3K64, H3K122 as well as H4K77 and H4K91, whereas acetylated counterparts on the same sites were not identified. Of note, the latter lysines are known to be acetylated at very low stoichiometry, which likely explains the difficulty to identify them in exploratory (DDA) LC-MS/MS analyses, in which peptide ions are selected for fragmentation based on their MS1 signal intensity.

### False Identifications of Presumably Lactylated Peptides Correspond to Propionylated and Oxidized Sequences, which can be of Prevailing Abundance

**Fig. 2:**
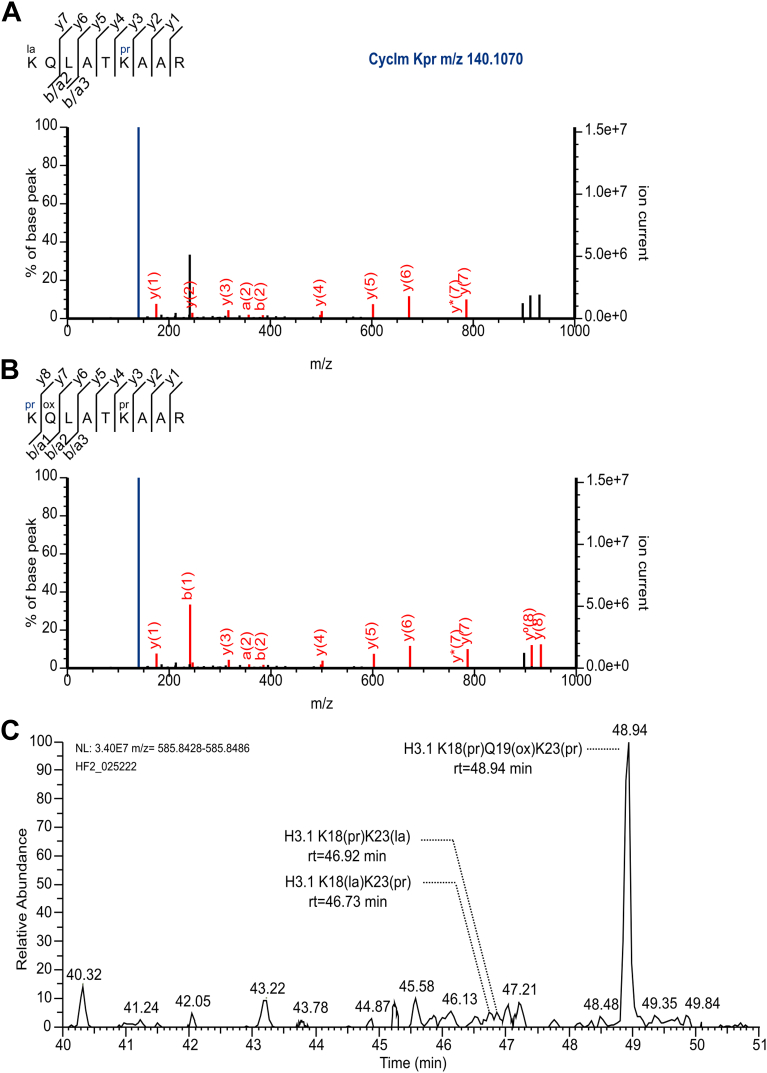
**Oxidized peptides lead to erroneous identifications of lactylated sequences.***A*, MS/MS spectrum tentatively matched by Mascot to H3 K18QLATK23AAR lactylated on the first lysine. The presence of an intense CycIm ion at m/z 140.107 that signs H3K18pr, and the absence of CycIm ion for Kla (expected at m/z 156.1019) and of b1, lead to rejecting this theoretical sequence. *B*, taking into account the oxidation of glutamine leads to the correct identification of H3 KprQoxLATKprAAR. *C*, relative abundances estimated by their MS1 XICs of H3 KQLATKAAR lactylated at either K18 or K23, and of KprQoxLATKprAAR.

When visually inspecting the MS/MS spectra attributed to lactylated peptides by the program Mascot, we rejected some identifications because they did not match our strict validation criteria. Manual processing of these spectra usually confirmed the proposed amino acid sequence, yet the absence of the CycIm ion for Kla refuted the lactylated nature of the fragmented peptide. Figure 2*A* shows an MS/MS spectrum presumably identifying peptide K_18_laQLATK_23_prAAR from H3. Yet, the dominant fragment at m/z 140.107 corresponding to the CycIm ion of Kpr reveals that the first lysine is propionylated instead, and supports positioning the mass difference between lactyl and propionyl at an upcoming residue. Considering that this mass difference is 15.9949 u, matching that of oxidation (ox), we reinterpreted our datasets with considering possible oxidation of various residues, including glutamine. This led to the attribution of the aforementioned spectrum to H3 K_18_prQoxLATK_23_prAAR, and specifically allowed interpreting the two highest-mass fragments as y/y_0_ ions (Fig. 2*B*). Chromatographic peaks extraction for strictly isobaric peptides H3 K_18_laQLATK_23_prAAR, K_18_prQLATK_23_laAAR and K_18_prQoxLATK_23_prAAR revealed a low, hardly quantifiable, MS signal for the two first peptides at very similar retention times (RT, about 46.7 min) and a much stronger MS signal for the oxidized peptide species at RT about 49 min (Fig. 2*C*). The oxidized peptide originates from H3 molecules which are endogenously nonmodified at H3K18 and H3K23 and constitute the most abundant proteoform. Comparing the MS intensities of K_18_prQoxLATK_23_prAAR and K_18_prQLATK_23_prAAR indicated that oxidation occurs at about 1.5% (Supplemental Table S8). Oxidation events likely occur during the harsh conditions required for TCA precipitation and during the reverse propionylation at pH 12.

Histone peptides modified by propionylation and with an oxidation on reactive residues can thus lead to erroneous identifications of lactylated peptides, and their abundance often overshadows that of actual lactylated peptides that really bear a 72.021 u modification on a specific lysine residue. To assess whether this phenomenon could be generalized, we considered other peptides from histones H3 and H4 and could confirm oxidation events at residues M (singly and doubly oxidized), Q, T, Y, and H (see Supplemental Fig. section S2). All these species contribute to the multitude of (histone variant × modifications) combinations that share the very same mass, and support the careful inspection of fragmentation spectra to avoid mis-identifications (23).

### Validating the Presence of L-Lactylation on H3K18 in Mouse Testis Histones and the Necessity to Also Envision D-Lactylation

Beyond the impact on identifications, the presence of propionylated and oxidized peptides in the samples hinders quantification of lactylated peptides based on their MS1 signal. To definitely validate the nature of lactylated peptides and reliably quantify them, it appeared necessary to synthesize lactylated peptides containing a C-terminal arginine labeled with naturally stable heavy atoms, namely ^13^C and ^15^N (Supplemental Table S1). These synthetic peptides, propionylated and then spiked into the histone samples, would strictly elute and fragment identically with their endogenous counterparts if the latter are truly lactylated. Because histones have been described to be modified by the L-lactyl enantiomer rather than the D-lactyl one, we first assessed this hypothesis (3, 32).

**Fig. 3:**
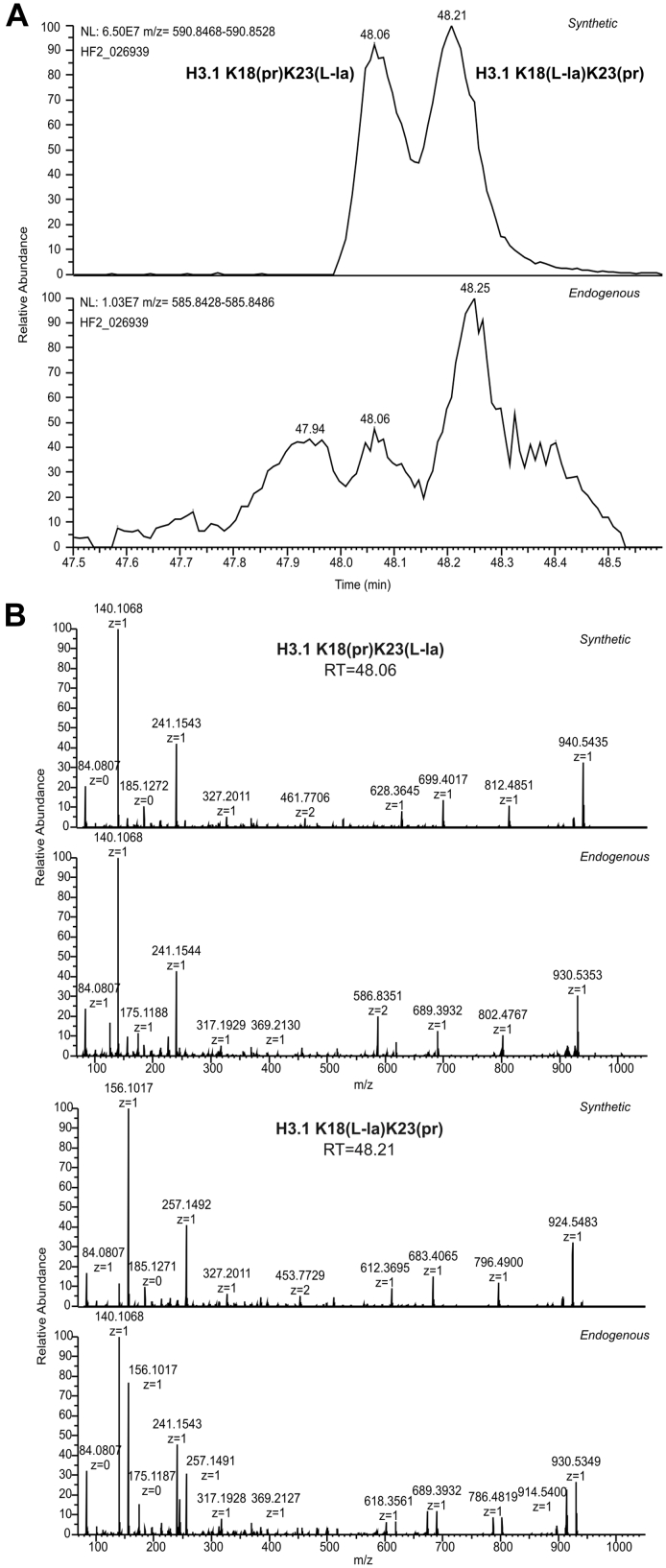
**Assessing the presence of L-lactylation at lysines H3K18 and H3K23 in histones from mouse testis.***A*, MS1 signal detected for the heavy-labeled peptides of sequences H3 K18(L-la)QLATK23prAAR and H3 K18prQLATK23(L-la)AAR (*top panel*) and corresponding MS1 signal in endogenous (*light*) mouse testis histones. *B*, MS/MS spectra acquired on the pairs of heavy/endogenous peptides at the apex of the two chromatographic peaks defined by the synthetic peptides.

Proteolyzed mouse testis histones were mixed with heavy labeled L-lactylated synthetic peptides covering a range of lysine residues from histones H3 and H4, and analyzed by LC-MS/MS, by DDA and PRM (Supplemental Table S2). Because H3K18la has been described and functionally characterized in various contexts (3, 12, 13, 33, 34, 35), we first scrutinized peptide H3 K_18_laQLATK_23_prAAR and its positional isomer K_18_prQLATK_23_laAAR. Chromatographic peaks extraction for the synthetic and endogenous peptides (m/z = 590.8498 (2+) and 585.8457 (2+), respectively) showed a good retention time match between the heavy and light sequences modified on either H3K18 or H3K23 (Fig. 3*A*). This observation suggested the presence of both endogenous peptides L-lactylated on H3K18 and H3K23. To fully validate their identity, we examined the MS/MS spectra acquired on the light/heavy pairs within the two extracted chromatographic peaks. In the first peak, MS/MS spectra acquired on the light and heavy species were identical, except for the 10.008 mass shift due to the ^13^ C/^15^N labeling, which validated the presence of peptide K_18_prQLATK_23_(L-la)AAR in mouse testis histones (Fig. 3*B*, two top panels). In contrast, in the second chromatographic peak, the MS/MS spectrum yielded by the light peptide corresponded to a composite spectrum generated by sequences lactylated at either H3K18 or H3K23 (Fig. 3*B*, two bottom panels). From these observations, we could validate the identification of H3 K_18_(L-la)QLATK_23_prAAR, together with another coeluting isobaric sequence H3 K_18_prQLATK_23_modAAR. We hypothesized that this modification could correspond to D-lactylation.

### Determination of the Chromatographic Behavior of Synthetic Peptides from Histones H3 and H4 Bearing L- or D-Lactylated Lysines

The peptides formerly synthesized with an L-lactylation on their lysine residues and bearing a heavy C-terminal arginine were similarly produced with D-lactylated lysines (Supplemental Table S1). To assess whether enantiomeric peptides bearing either lactyl moiety could be chromatographically separated, all synthetic peptides were propionylated, mixed in variable D:L abundance ratios (1:1, 1:2, and 2:1) and analyzed by LC-MS/MS.

**Fig. 4:**
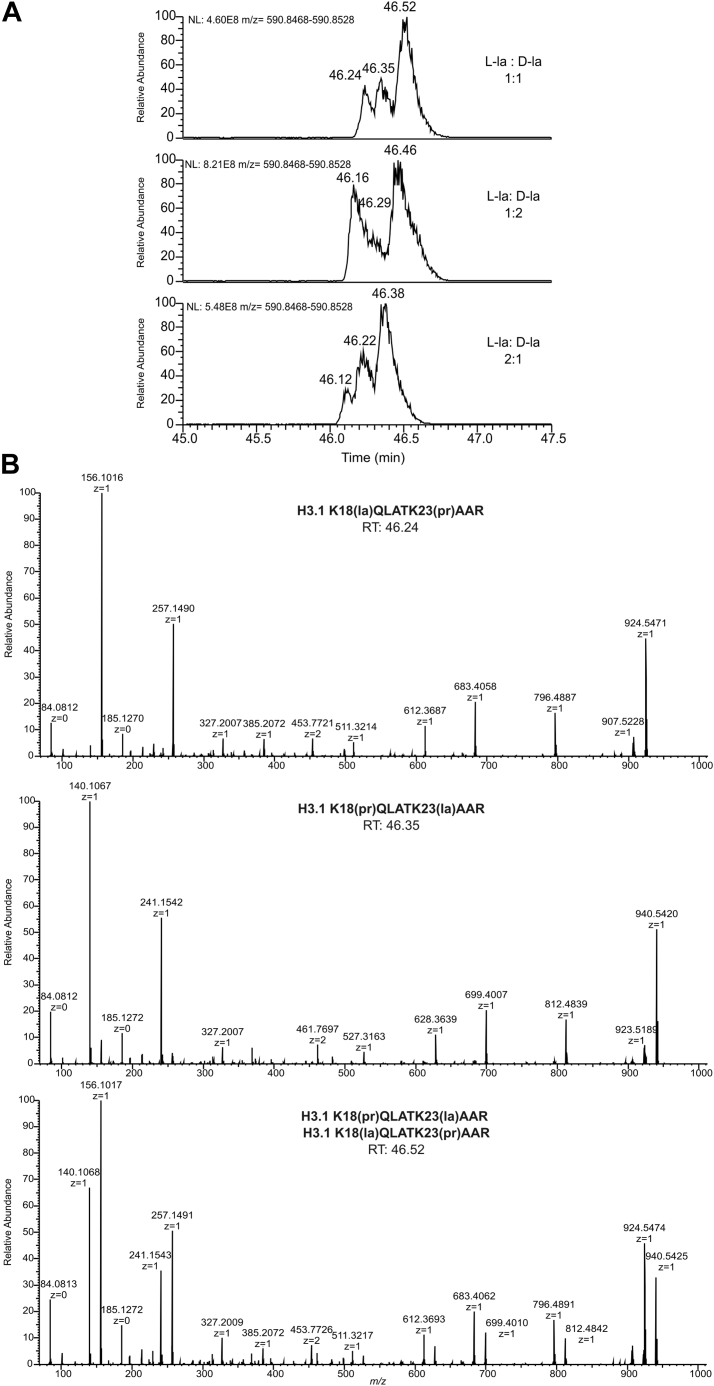
**Establishment of the elution order of L- and D-lactylated peptides of same sequence.***A*, MS1 signals detected when analyzing three mixtures of synthetic peptides H3 K18QLATK23AAR, being L- or D-lactylated at either H3K18 or H3K23, in relative molar ratios 1:1, 1:2, and 2:1. *B*, MS/MS spectra acquired at the apex of the three successive chromatographic peaks.

The four peptidoforms corresponding to sequence H3 K_18_QLATK_23_AAR yielded three chromatographic peaks (Fig. 4*A*). The fragmentation spectra at the apex of these peaks in the 1:1 mixture allowed identifying successively the modified peptides K18(la)K23(pr), then K18(pr)K23(la), and finally both positional isomers in the third chromatographic peak which exhibited a doubled intensity (Fig. 4*B*). The elution order between the D- and L-lactylated peptides could be established from the MS1 signal intensities measured in the mixtures of imbalanced ratios: the K18(D-la)K23(pr) form elutes first, followed by K18(pr)K23(L-la), and finally the two positional isomers K18(pr)K23(D-la) and K18(L-la)K23(pr) coelute. In spite of their coelution, these two peptidoforms can be distinguished by their fragmentation profiles. In all, MS2-based quantification allows characterizing all four peptide species L- or D-lactylated at H3K18 or H3K23.

**Fig. 5:**
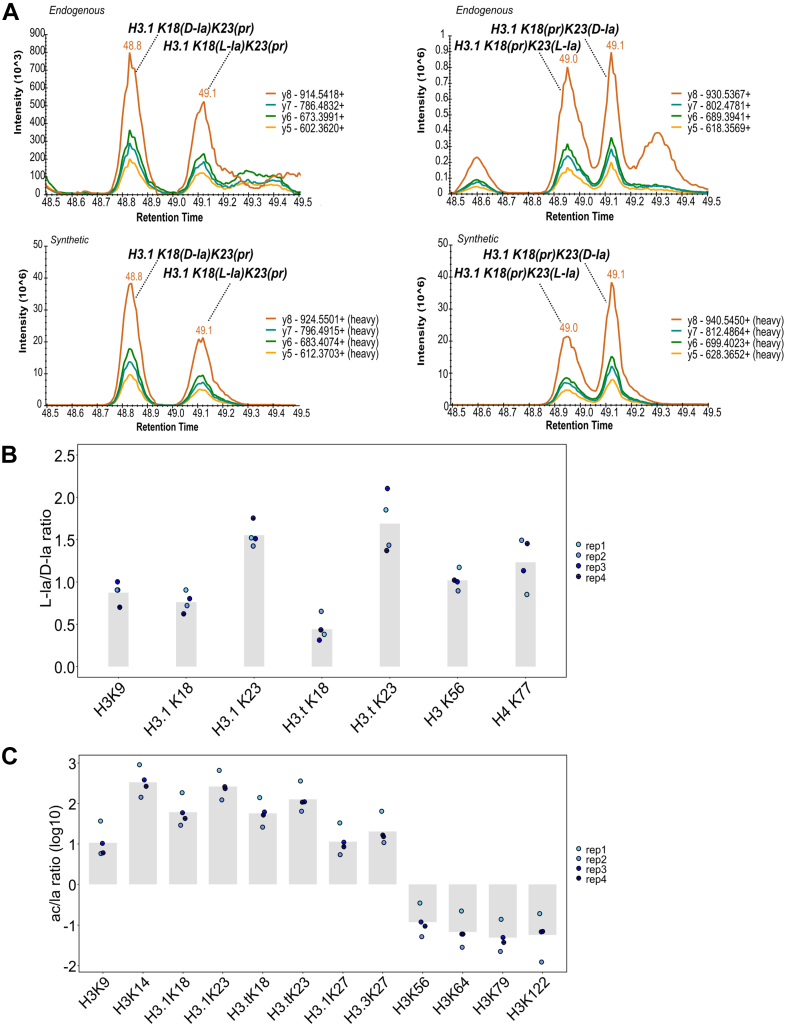
**Validation of the coexistence of L- and D-lactylation on lysine residues of mouse testis histones H3 and H4 for which the two enantiomers are chromatographically separated, and abundance ratios to one another and compared to acetylation.***A*, detection by selected y fragments of the presence of L- and D-lactylation on H3K18 (*left panels*) and H3K23 (*right panels*) in mouse testis histones. These fragments exhibit a perfect match between the endogenous (*light*) and heavy (*synthetic*) peptides. *B*, comparison of the abundances of L- and D-lactylation at the lysine sites for which the two enantiomers are chromatographically separated, in four biological replicates. *C*, abundance ratio of acetylation and lactylation at lysine residues of histone H3, in four biological replicates.

The LC-MS/MS analyses of the three mixtures of L- and D-lactylated peptides revealed that a series of enantiomeric peptides could be chromatographically separated on the C18 Aurora column: these were peptides bearing lactylation on H3K18 and H3K23 from both canonical H3 and testis-specific H3 (H3.t, sequence K_18_QLATK_23_VAR), and H3K56 (Supplemental Figs. section S3). The resolution of chromatographic separation for these positional isomers was estimated to lie between 1.2 and 2.1, by considering MS2-fragment-defined chromatographic peaks visualized in the program Skyline (Fig. 5 and Supplemental Table S9). Enantiomers modified at H3K9, H3K79 and H4K77 were partially separated. However, no chromatographic separation was obtained of peptides being L- or D-lactylated at H3K14, H3K27 (from both canonical H3 and variant H3.3), H3K64, H3K122, H4K5/K8/K12/K16, H4K31, H4K79, and H4K91. Of note, the peptide bearing the two latter lysines (*i.e*., K_79_TVTAMDVVYALK_91_R) also contains a methionine that was observed in several oxidation levels, which impacted the chromatographic behavior of D/L-lactylated peptides (Supplemental Fig. section S3). Due to the complexity of following this peptide distributed over variably oxidized forms, we did not retain it in the following parts of the study.

### Systematic Analysis of L- and D-Lactylation in Histones H3 and H4 from Mouse Testis and Comparison to Their Acetylated Counterparts

To assess the existence of L- and D-lactylation in mouse testis histones, four biological replicates were spiked with synthetic peptides bearing the two enantiomeric PTMs in a 1:1 ratio, and analyzed by targeted LC-MS/MS (Supplemental Table S3). The four peptidoforms of H3 K_18_QLATK_23_AAR could be reliably detected based on discriminative fragments shared between the endogenous and the synthetic peptides (Fig. 5*A*), which validated our original assumption that this sequence is modified by L- and D-lactyl on its two lysine residues. We determined that the enantiomers which could be chromatographically resolved had a relative L/D abundance lying between 0.4 and 1.6 (Fig. 5*B*). The lowest L/D ratios were measured on H3K18 and the highest on H3K23 (Supplemental Table S10). Statistical assessment of MS2 signals indicated a significant excess of D-lactylation at H3K18 and inversely an excess of L-lactylation at H3K23, both on canonical H3 and testis-specific H3 (H3.t) (Supplemental Fig. section S4). Besides, H3K9, H3K56, and H4K77 anchored balanced amounts of L- and D-lactylation. H3K31la could not be properly quantified, due to signal interferences coming from a highly abundant species (Supplemental Fig. section S5). Finally, intriguingly, the endogenous signal for H3K79la essentially matched the D-lactylated synthetic peptide, indicating that this specific residue bore mostly D-lactylation (Supplemental Fig. section S6). Because histone L-lactylation was previously described to correlate with the cellular concentration of L-lactate (36), we asked whether the balanced histone modification by L- and D-lactylation observed on mouse testis histones could be explained by similar concentrations of L- and D-lactate in this organ. Whereas the L-enantiomer could be reliably quantified at an estimated concentration of 1.4 ± 0.16 nmol per mg tissue, the signal detected for D-lactate by colorimetric assay was too close to the detection limit to be reproducibly quantified under our experimental conditions (see Material and Methods). We could estimate at least a ten-fold concentration ratio between both metabolites in favor of L-Lactate. These results indicate that the similar levels of L- and D-lactylation on mouse testis histones do not reflect the relative concentrations of L- and D-lactate in this tissue. They also imply that metabolites other than D-lactate contribute to histone D-lactylation.

Next, we aimed to compare the abundance ratio of lactylation to the canonical PTM acetylation at the same lysine sites. We performed a targeted analysis of the four biological samples of mouse testis histones spiked with the L- and D-lactylated, as well as the corresponding acetylated synthetic peptides. Of note, we observed that the retention times of lactylated and acetylated peptides were very close, whatever the peptide sequence (Supplemental Fig. section S7). The abundance of variably modified peptides was estimated by integrating the signals of a few y-type fragments, the same series being selected for acetylated and lactylated peptides (see Supplemental Table S3). Interestingly, acetylation appeared to be much more abundant at lysines of the N-terminal tail of both histones H3 and H4, whereas lactylation became more abundant in the histone fold domain (Fig. 5*C* and Supplemental Fig. section S8). Of note, acetylation stoichiometries in the C-terminal halves of H3 and H4 are very low.

Finally, we sought to get an estimation of the stoichiometry of lactylation at all investigated lysine residues. Having formerly determined the abundance ratio ac/la, we obtained the relative abundance of acetylation. Exploratory analyses by DDA provided this information for all lysines of the N-terminal tails of histones H3 and H4, which are of sufficient abundance to be automatically selected for fragmentation (Supplemental Table S4). Besides, to estimate the stoichiometry of acetylation at lysines of the histone fold domains, we designed PRM analyses targeting the acetylated peptide and all variably modified peptide forms of same sequence, whose summed MS signals represented more than 90% of the MS signal for this sequence (Supplemental Table S5). The quantitative results of these analyses are provided in Supplemental Table S11. Finally, the estimated stoichiometry of acetylation and lactylation at all lysine sites are shown in Table 1. The well-known discrepancies in acetylation levels between H4K5/K8/K12 and H4K16, or between H3K9 and H3K14, were observed in our analyses of mouse testis histones. By contrast, lactylation levels at these compared sites were of very similar relative abundances, lying around 0.1%.

**Table 1:** Estimated relative abundances of acetylation and lactylation at all investigated lysines from histones H3 and H4

Histone	Sites	Ac		L-la		D-la		Coeluted	
		Mean	SD	Mean	SD	Mean	SD	Mean	SD
H3	K9	0.87%	0.23%	0.04%	0.02%	0.05%	0.04%		
	K14	22%	3.24%					0.08%	0.05%
	H3.1 K18	10.5%	2.6%	0.08%	0.04%	0.11%	0.06%		
	H3.1 K23	28%	0.8%	0.08%	0.04%	0.05%	0.03%		
	H3.t K18	9.1%	2.7%	0.05%	0.02%	0.11%	0.04%		
	H3.t K23	25.6%	2.9%	0.14%	0.07%	0.09%	0.05%		
	H3.1 K27	0.08%	0.02%					0.01%	0.01%
	H3.3 K27	0.22%	0.01%					0.01%	0.01%
	K56	0.02%	0.002%	0.08%	0.04%	0.08%	0.05%		
	K64	0.002%	0.0002%					0.04%	0.03%
	K79	0.01%	0.001%			0.4%	0.21%		
	K122	0.02%	0.003%					0.44%	0.43%
H4	K5/8/12	3.8%	1.2%					0.16%	0.067%
	K16	34.5%	1.4%					0.07%	0.05%
	K77	0.01%	0.001%	0.04%	0.03%	0.03%	0.02%		

Rel. Ab.: relative abundance of a PTM at the considered lysine site. The indication of the respective amounts of L- and D-lactylation is provided when the peptidoforms bearing either enantiomer could be chromatographically separated. An average abundance is indicated for residues H4K5, H4K8, and H4K12, whose positional isomers (both the acetylated and the lactylated ones) are insufficiently separated by LC to get their individual quantification.

### SC and round Spermatids Also Exhibit Histones Modified by a Racemic Mixture of L- and D-Lactylation

To increase the resolution of our analyses, we next investigated the predominant cell types of the testis, SC and spermatids, which are known to heavily rely on the L-lactate provided by the surrounding Sertoli cells (18). We collected enriched fractions of primary SC and RS, and extracted their histones. Targeted PRM analyses of histone samples prepared in triplicate by in-gel biochemical processing allowed identifying all former lactylated peptides (see gel shown in Supplemental Fig. section S9). Yet, among the enantiomers exhibiting chromatographic separation, only those modified on H3K18 and H3K23 from canonical H3 and TSH3 gave reliably quantifiable MS2 signals (Supplemental Fig. section S9). This may be due to the loss of sensitivity stemming from the lower starting biological material, compared to when formerly working on histones extracted from testis nuclei. The four peptidoforms appeared to be modified by a racemic mixture of L- and D-lactylation. This observation would indicate that another pool of metabolite than L-lactate fueled into germ cells by Sertoli cells contributes to histone lactylation in SC and RS.

## Discussion

Our study pioneers in characterizing by proteomics the presence of L- and D-lactylation in a healthy mouse tissue, when the majority of reports describing histone lactylation worked on immortalized cells or on tumors and most often relied on antibody-based experiments. We determined that lactylation occurred on a large number of lysine residues from histones H3 and H4 at a very low and quite stable estimated stoichiometry of 0.01 to 0.44% in mouse testis. This observation is in stark contrast with relative abundances of acetylation, which are highly variable between lysine sites, including the neighboring H3K9 *versus* H3K14 and H4K5/K8/K12 *versus* H4K16, being of low (1–5%) or high (22–35%) stoichiometry, respectively. Interestingly, while acetylation dominates on the N-terminal tails of H3 and H4 which are well accessible to HATs, lactylation becomes comparatively more abundant than acetylation in the histone fold domains. In H3 and H4 core residues, where acetylation represents a 0.002 to 0.02% relative abundance, our quantitative proteomic analyses allowed estimating that lactylation could be about 8 times higher at H3K56 or H4K77, and could exist in a striking 40-times higher abundance at H3K79. Of note, given the very low stoichiometries of both acetylation and lactylation on the C-terminal part of H3 and H4, and the possibly different ionization efficiencies of peptides bearing either modification, the observed relative abundances may not be devoid of biases. Yet our data indicate that lactylation can exceed acetylation at certain low-acetylation C-terminal sites. Our work further shows for the first time that some histone peptides modified by either L- or D-lactyl can be separated on a C18 column, when performing *in vitro* propionylation of free lysines before and after trypsin digestion. We could thus distinguish modification by the two enantiomers on H3K9, H3K18 and H3K23 on both canonical H3 and testis-specific H3, as well as H3K56, H3K79 and H4K77. Some of these residues bore a balanced amount of D- and L-lactyl, with the exceptions of H3K18 and H3K79 which exhibited more or almost exclusive D-lactylation, whereas H3K23 appeared more modified by L-lactylation. We further refined our analysis on histones extracted from SC and RS, which exhibited a racemic mixture of L- and D-lactylation at H3K18 and H3K23. These results obtained from a healthy mouse tissue contrast sharply with previous reports (32, 36), which identified L-lactylation as the dominant enantiomer on histones in the breast cancer cell line MCF-7, a nonphysiological model. Importantly, consistent with these studies, we ruled out the presence of carboxyethyl (Kce) at H3K18 in our samples (Supplemental Fig. section S10).

To reliably identify and quantify lactylation in histones from mouse testis, we considered synthetic peptides singly modified with this acylation at a lysine residue. However, most of these proteolytic peptides contain two or more lysines, and the lactylation site may coexist with other modifications *in vivo*. For example, we detected H3K18la with H3K23ac in our testis histone samples. Besides, H3K9la was identified by proteomics in association with H3K14ac in HeLa cells (11). Of note, both H3K14 and H3K23 are acetylated at high stoichiometries in most cell types, so that the combination of these acetylation sites with a lactylation on the neighboring lysine may be quite abundant and thus detectable by proteomic analysis. A similar reasoning may apply to the stretch H3K27-R40, in which acetylation of H3K27 is more abundantly detected in association with H3K36me2 in various tissues (*e.g*. mouse testis and brain), especially on variant H3.3. It might thus be worth exploring the hypothesis of the combination of PTMs H3 K27la/K36me2, using doubly modified synthetic peptides.

Based on the herein presented results, future work should inquire (i) whether the modification by L- and D-lactyl is also observable on other lysines of histones H3 and H4 from mouse testis, and beyond, on histones H2A, H2B, and H1, possibly by implementing ion mobility (37) or a chiral reaction to increase the chromatographic separation of variably modified peptides (32, 38); (ii) whether the two enantiomers modify histone lysines in other mouse organs, but also in organs of other mammals including humans; (iii) and finally, what enantiomers of lactyl modify nonhistone proteins in the former tissues.

L-lactylation has been most often considered to be the enantiomer that modifies protein lysines (9, 10, 32), due to the much higher concentration of L-lactate in mammalian tissues and plasma compared to D-lactate (39). This assumption is either explicitly expressed or is occulted in studies describing enzymes endowed with lactyltransferase activity. Although we confirmed that L-lactate concentration largely surpasses that of D-lactate in mouse testis, our analyses of histone lactylations reveal a third scenario that differs from formerly described ones, which consisted of the exclusive lactylation of proteins by either L- or D-lactylation (6, 32, 36). Our findings suggest the need to explore alternative mechanisms to account for the coexistence of L- and D-lactylation. One hypothetical mechanism to consider in future studies involves the glyoxalase DJ-1 (also named PARK7) which would provide the necessary mixture of L- and D-lactate. Indeed, DJ-1 was originally characterized in 2012 as a glyoxalase that converts methylglyoxal to lactic acid (40). More recently, Gao *et al.* used mass spectrometry and Western Blot to demonstrate that DJ-1 is allosterically activated by GSH and that it produces a mixture of L- and D-lactate, whose relative amounts, while always in favor of L-lactate, depend on the presence of GSH or of a glycated peptide (41). In parallel, Zhou *et al.* described the production of a racemic mixture of L- and D-lactate from MGO by DJ-1 in the absence of GSH (42). In all, our observation of histone L- and D-lactylation in mouse testes combined with these former publications encourages exploring the hypothesis of a production of a mixture of D- and L-lactate from MGO by DJ-1 in the chromatin vicinity, especially since this protein was formerly described to be chromatin-linked (43, 44). In addition to DJ-1, other known MGO scavengers (*i.e*. GLO1 and GLO2 (6), but also aldo-keto reductases AKRs and aldehyde dehydrogenases ALDHs (45)) could be of interest. Notably, the genes coding for these enzymes exhibit expression levels in the top two quartiles in mouse SC and RS (46). Whether they contribute to the production of lactylation intermediates and subsequent histone modifications remains to be investigated.

Beyond the production of L- or D-lactate, the question whether lactylation occurs by spontaneous chemical reaction or is mediated by an enzyme, such as p300, AARS1/2, HBO1 and/or KAT2A remains open. Given that we showed lactylation to be present at a quite stable stoichiometry over all lysine sites of histones H3 and H4, in stark contrast to acetylation events, a chemical addition may be a valid hypothesis to explore. In all cases, the potential regulatory functions of lactylation on gene expression, at lysines located in the N-terminal tail but also in the histone fold domain, where we showed that lactylation can be more abundant than acetylation, should be explored. When pursuing this quest, the herein determined low stoichiometries of lactylation should prompt researchers to systematically acquire in parallel the whole genomic distributions of the lactylated and acetylated counterparts (*e.g*. H3K18la and H3K18ac) by chromatin immunoprecipitation followed by high-throughput sequencing or cleavage under targets and tagmentation technologies. This would allow verifying the occurrence of specific peaks for lactylation, which would not be due to cross-reactivity of the anti-Kla antibody with the possibly more abundant acetylated site. Such establishment of genome-wide distributions of lactyl/acetyl paired marks was performed in the original description of histone lactylation focusing on H3K18 (3) but was not systematically carried out in later studies.

## Data Availability

Mass spectrometry DDA and PRM RAW data and associated results files were deposited on the ProteomeXchange Consortium (http://www.proteomexchange.org) *via* PRroteomics IDEntification Database (PRIDE) (47, 48) and Panorama Public (49) repositories under the identifiers PXD070921 and PXD070274, respectively.

## Supplemental Data

This article contains supplemental data: an Excel file containing Supplementary Tables 1–11; a PDF file containing supplementary figures, organized in 10 sections.

## Conflict of Interest

The authors declare no competing interests.
