# Supplementary material for: Both L-Lactyl and D-Lactyl Enantiomers Modify Histones in Mouse Testis
Source: Mol Cell Proteomics. 2026 Jun 12;25(7):101601. doi: 10.1016/j.mcpro.2026.101601 (PMC13380202; doi:10.1016/j.mcpro.2026.101601)
Supplement: Supplemental Figures [file mmc1.pdf]

# Both L-lactyl and D-lactyl enantiomers modify histones in mouse testis

Julie Manessier, Hassan Hijazi, Lisa Vizzini, Sabine Brugière, Marie Courçon, Christophe Masselon, Alberto de la Iglesia, Julie Cocquet, Delphine Pflieger

Supplementary figures

Section 1: Identification by proteomics of lactylated sites on histones H3 and H4 from mouse testis.
Section 2: False identifications of presumably lactylated peptides correspond to propionylated and oxidized peptides, which can be of prevailing abundance.
Section 3: Determination of the chromatographic behavior of synthetic peptides from histones H3 and H4 bearing L- or D-lactylated lysines.
Section 4: Statistical assessment of the abundance difference between L- and D-lactylated peptides that are chromatographically separated.
Section 5: Reason why H4K31la could not be quantified in the four biological replicates of mouse testis histones.
Section 6: MS2 signals for endogenous and heavy H3K79la-containing peptides show that the endogenous peptide essentially matches the D-lactylated synthetic peptide.
Section 7: Comparison of the retention times of acetylated peptides and their lactylated counterparts.
Section 8: Comparison of the abundances of acetylated peptides and their lactylated counterparts on H4.
Section 9: Analysis of histones from spermatocytes and round spermatids.
Section 10: Verification of the absence of the isomeric structure with lactyl, N-ε-(carboxyethyl).

Section 1: Identification by proteomics of lactylated sites on histones H3 and H4 from mouse testis

Gel obtained on histones extracted from nuclei of mouse testis

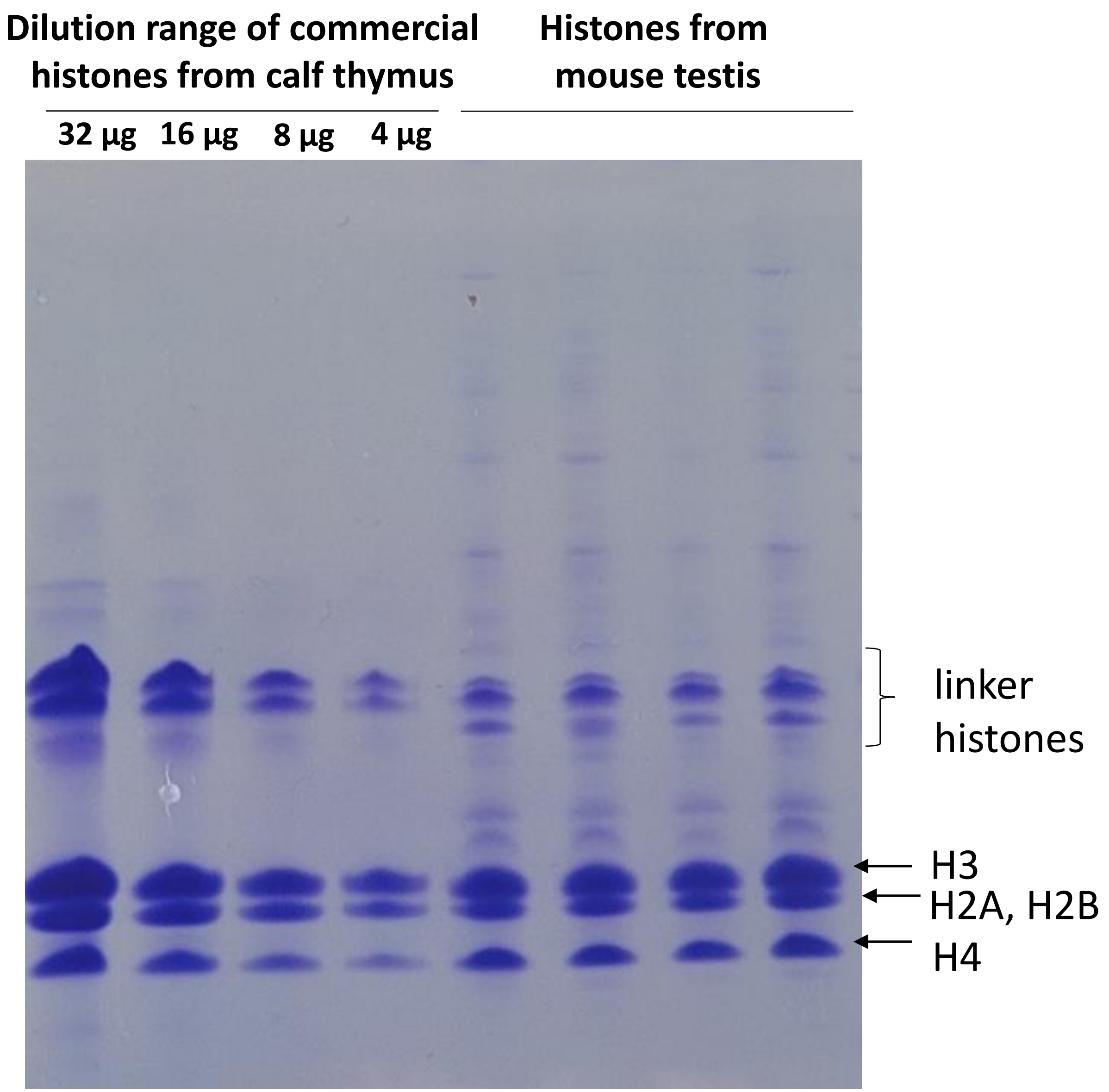

H3 K9STGGK14APR

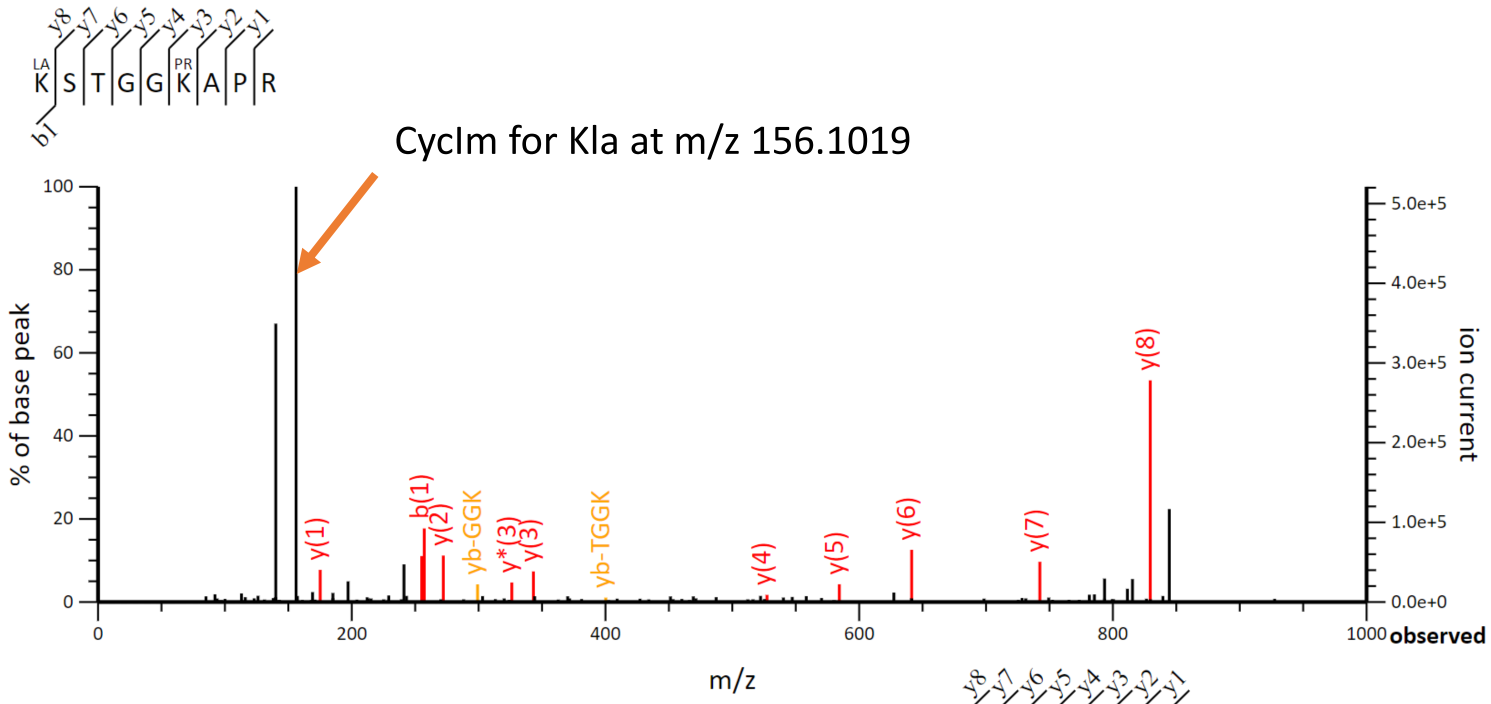

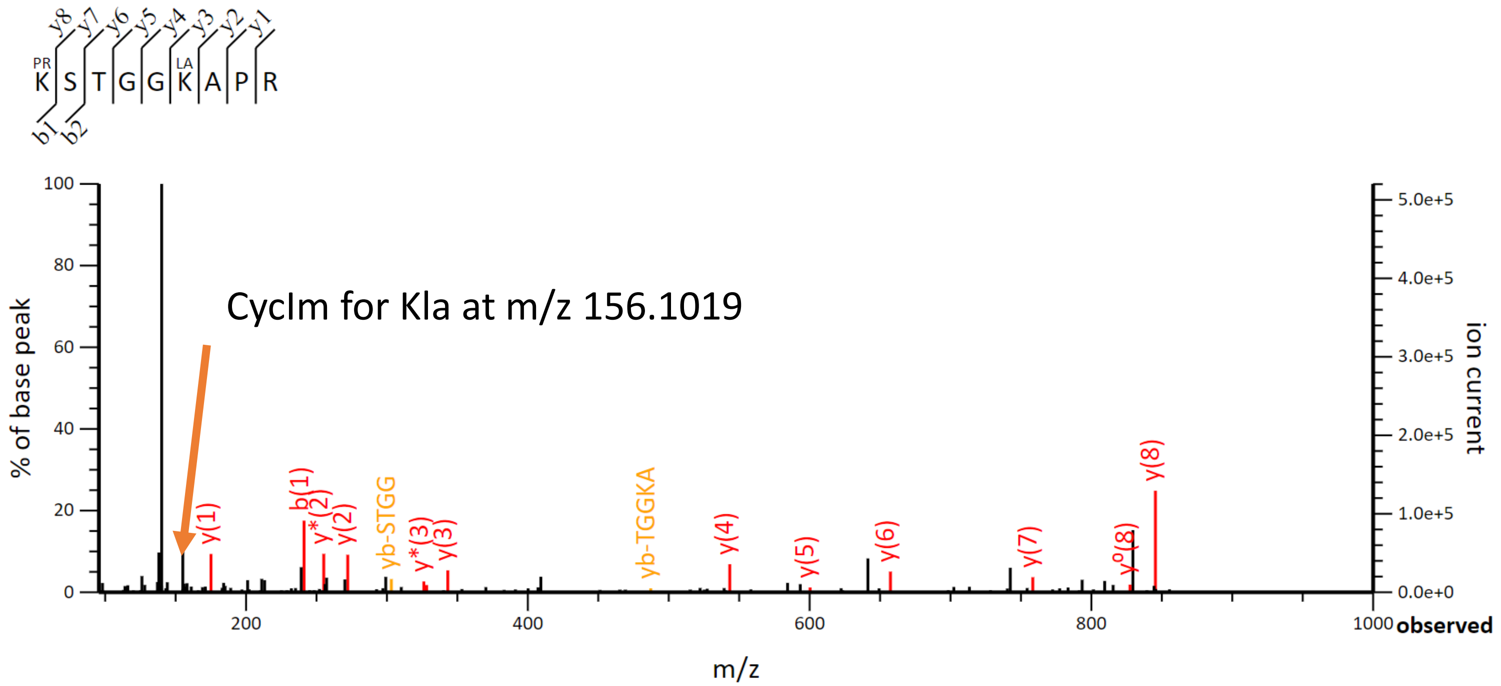

H3.1 K18QLATK23AAR

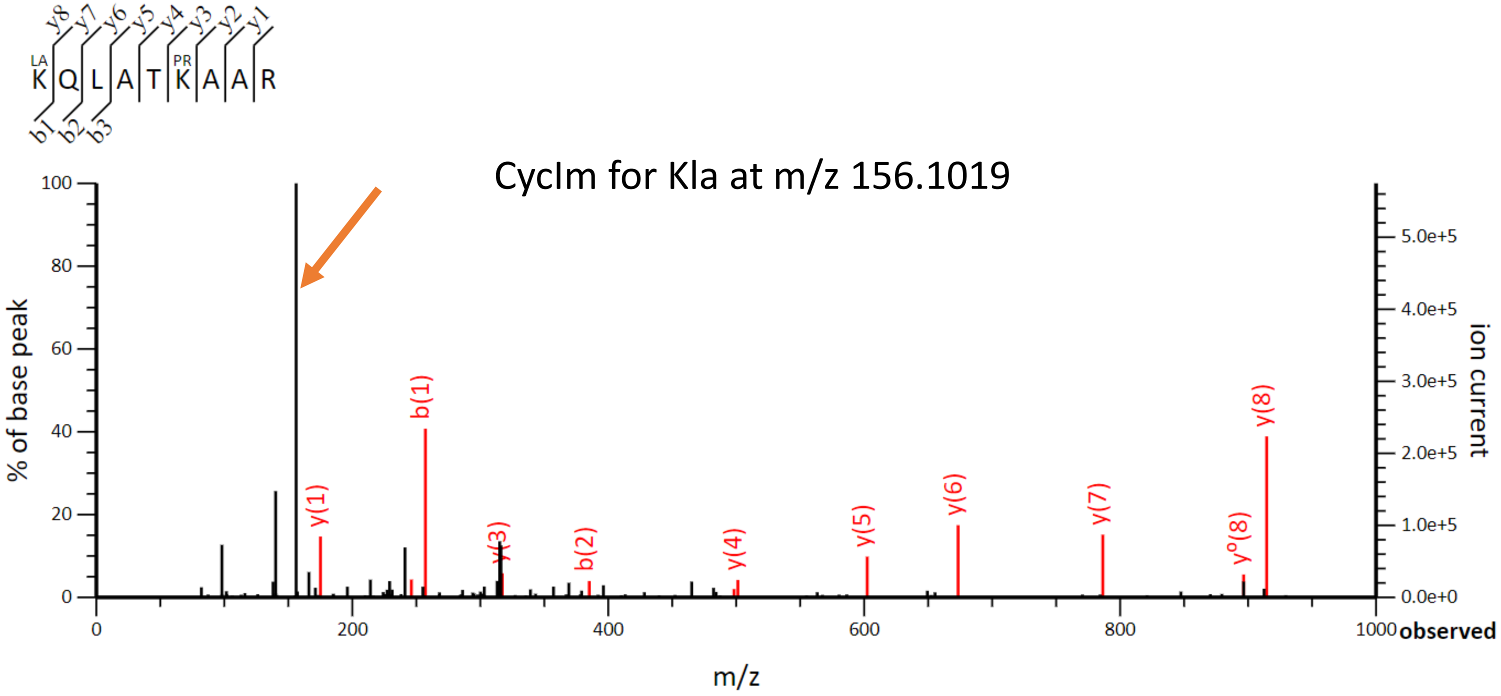

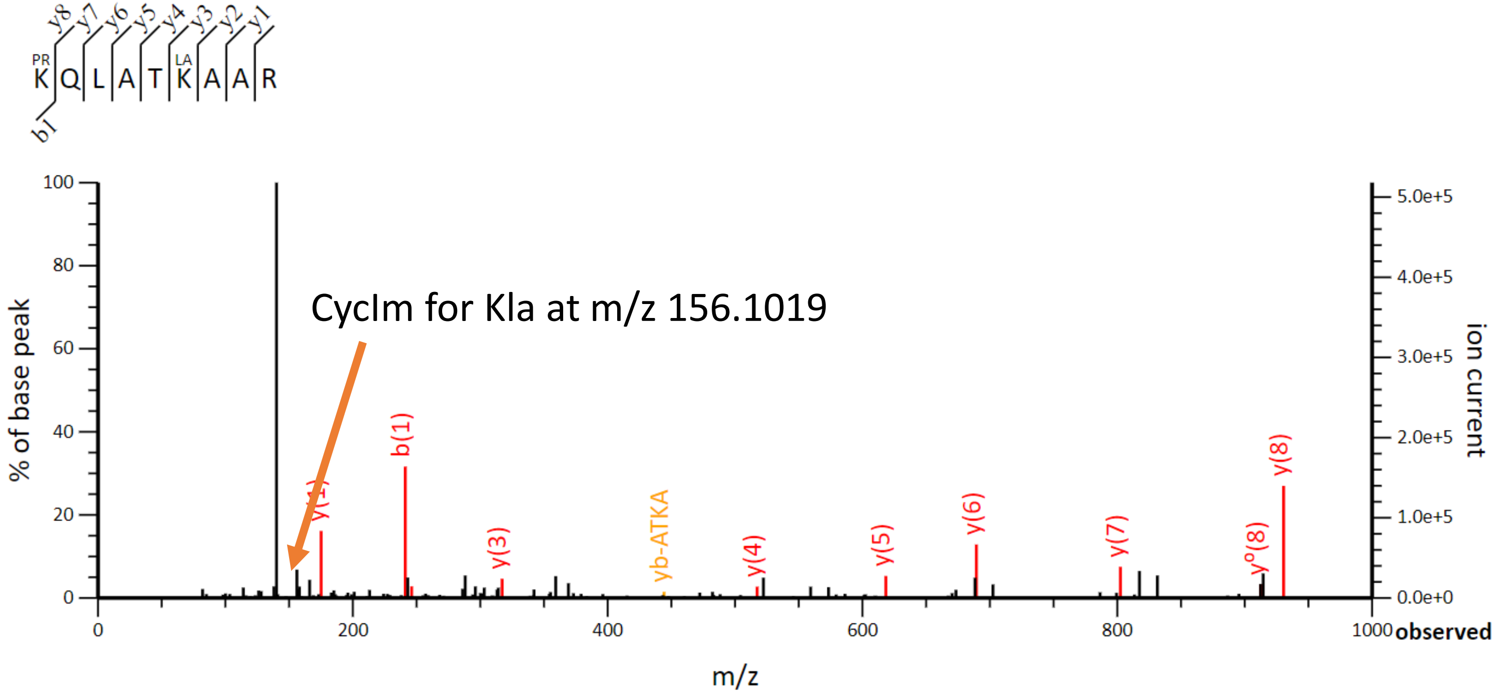

H3.T K18QLATK23VAR

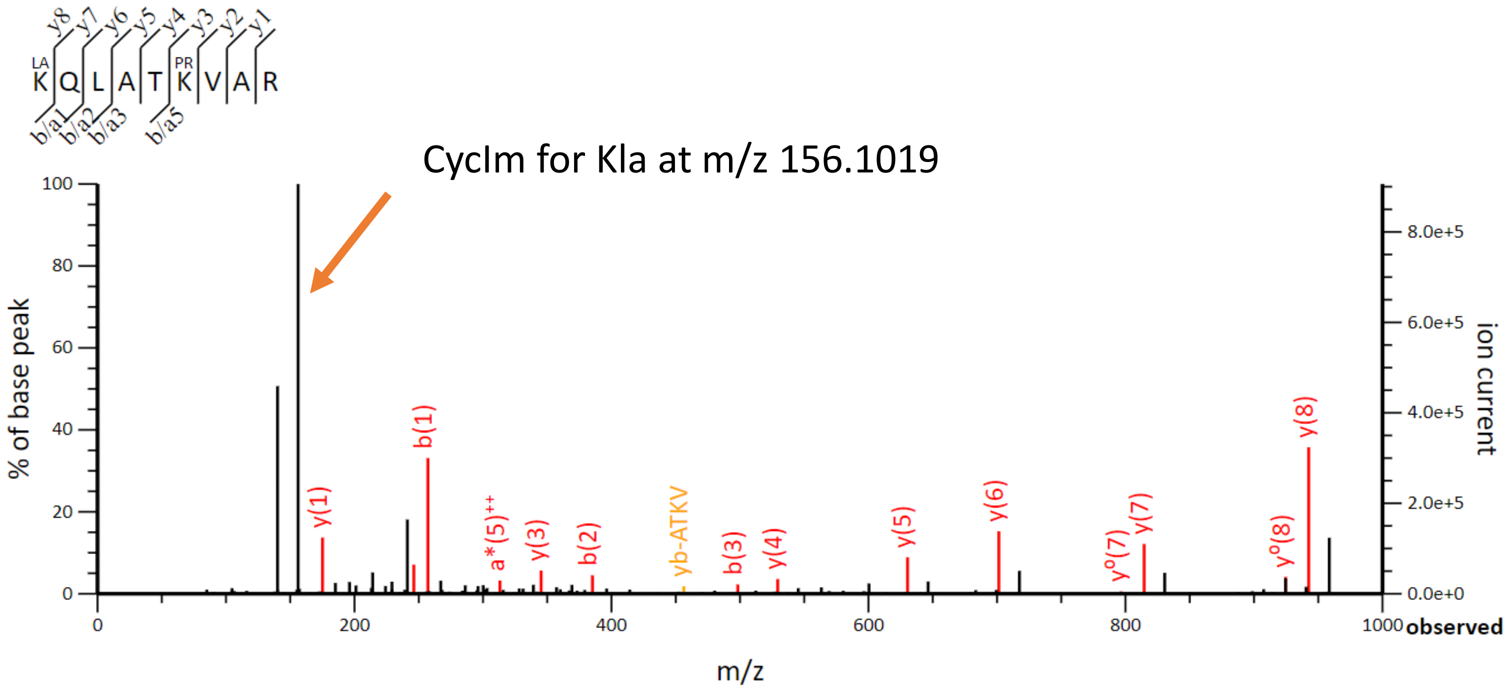

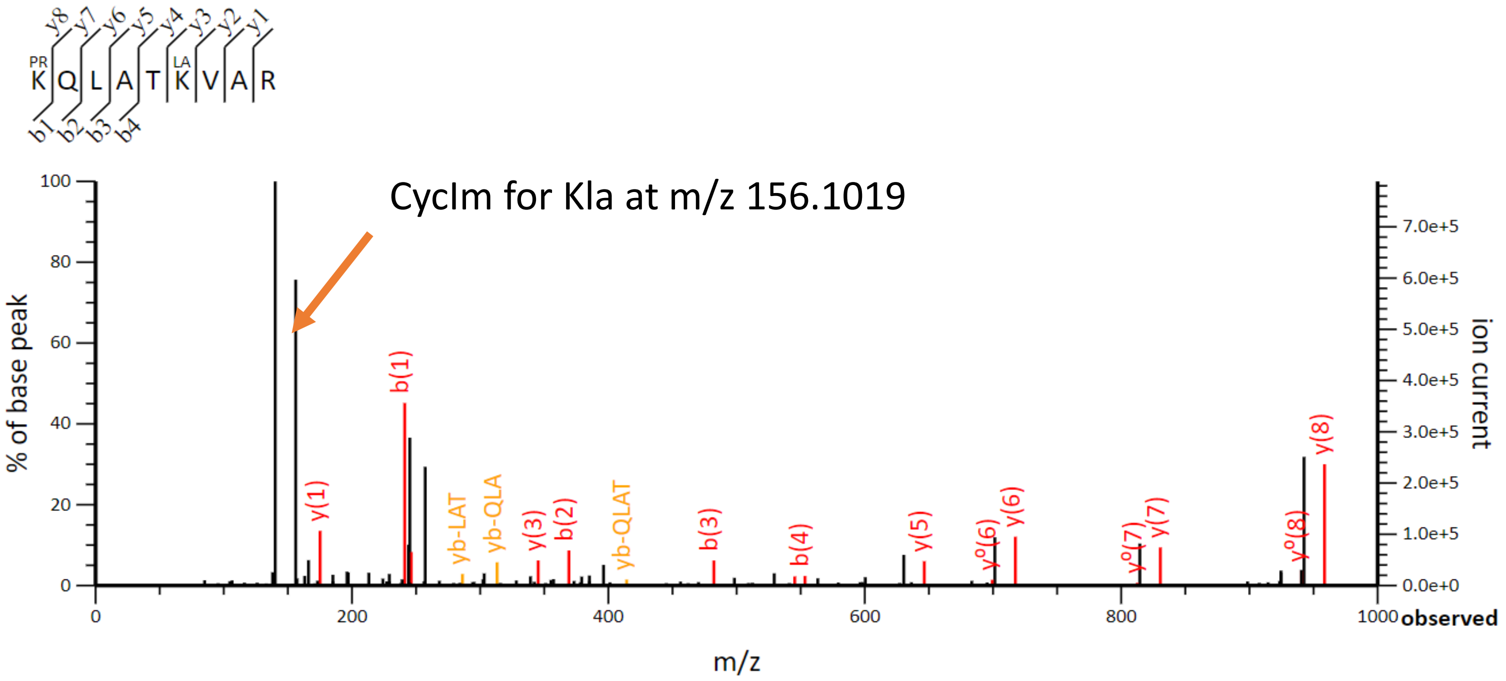

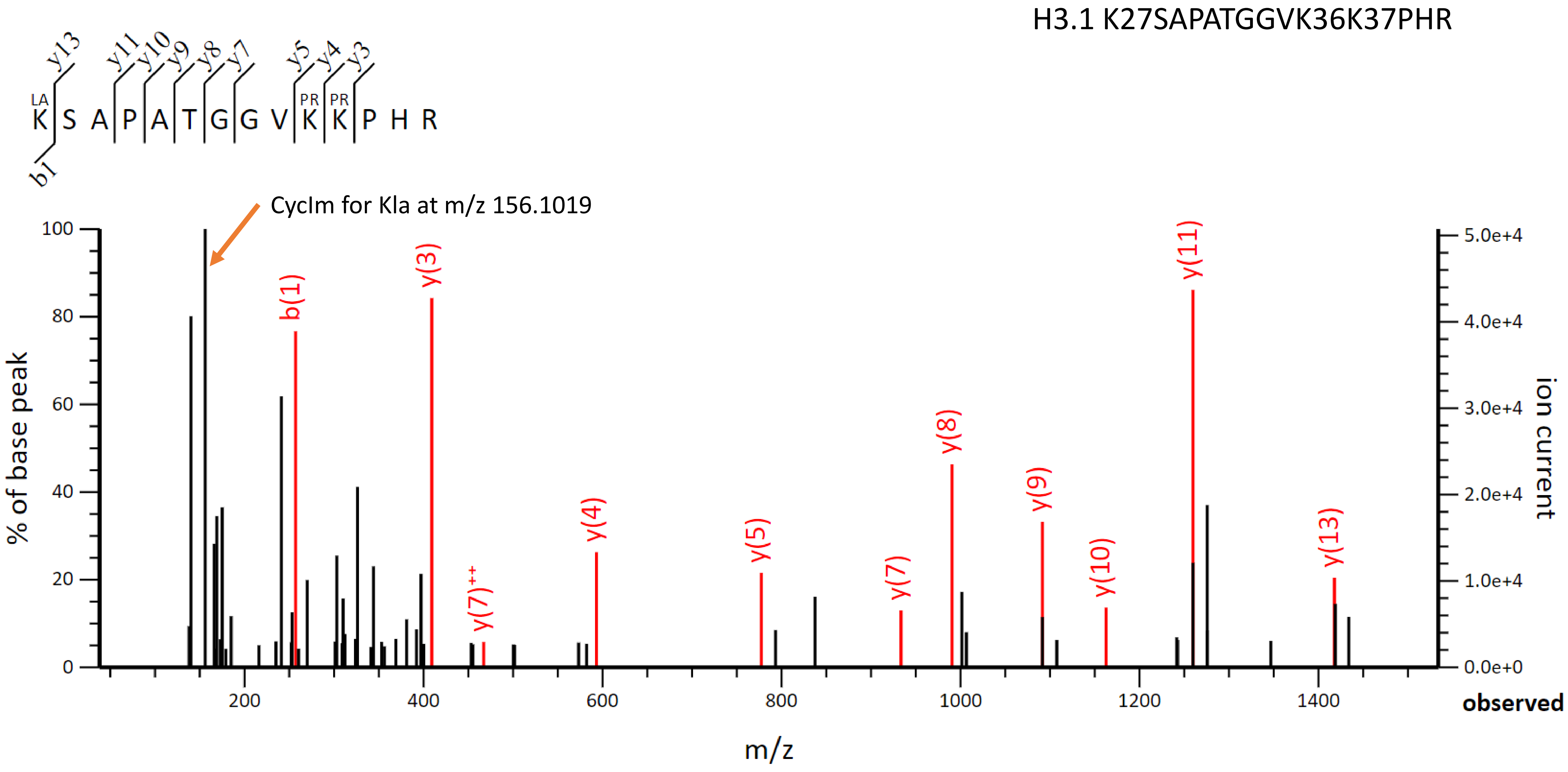

H3.1 K27SAPATGGVK36K37PHR
K S A P A T G G V K K P H R
LA
PR
PR
y13 y11 y10 y9 y8 y7 y5 y4 y3
b1
CycIm for Kla at m/z 156.1019
% of base peak
ion current
m/z
observed
b(1)
y(3)
y(7)++
y(4)
y(5)
y(7)
y(8)
y(9)
y(10)
y(11)
y(13)

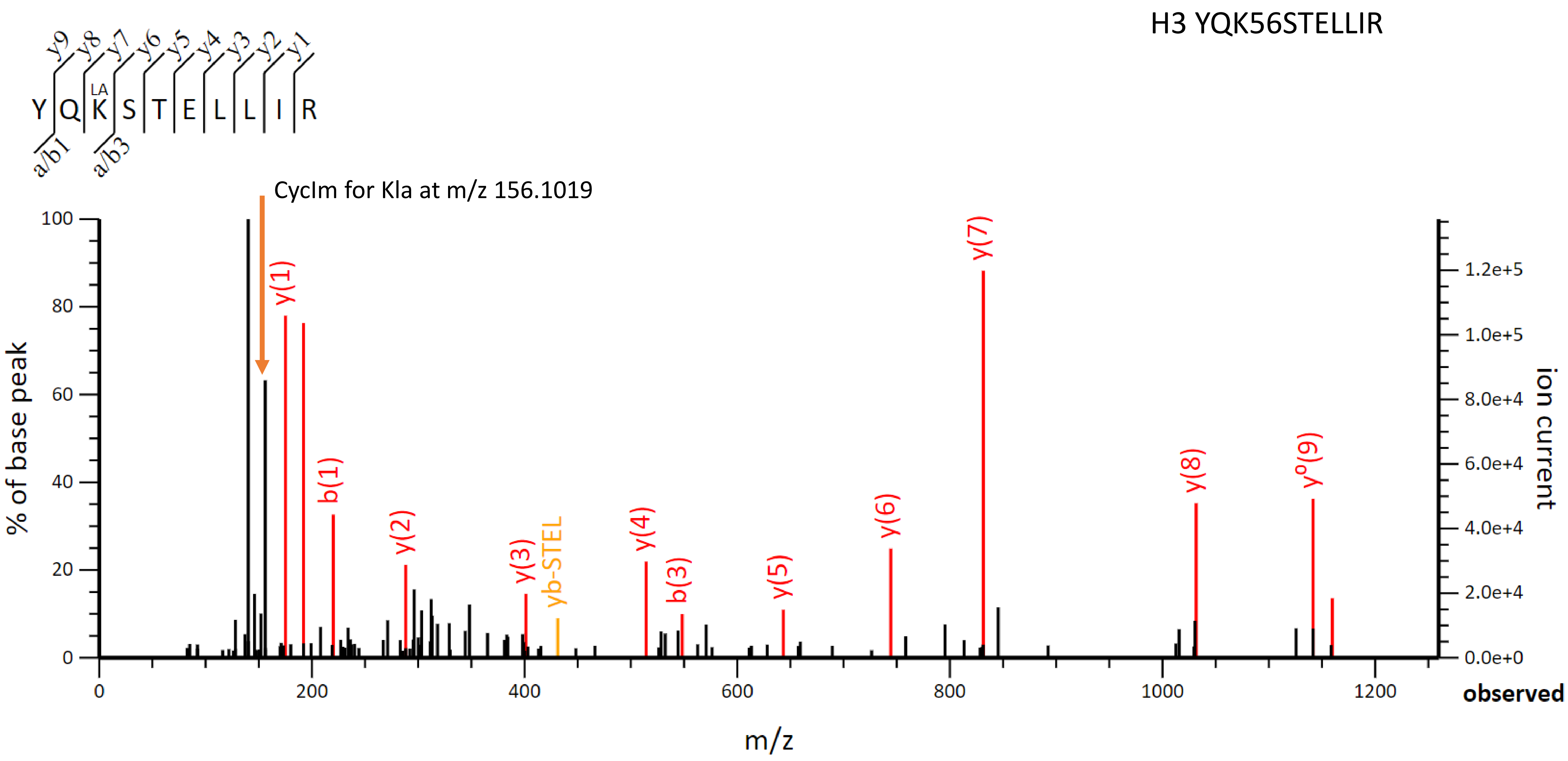

H3 YQK56STELLIR
y9 y8 y7 y6 y5 y4 y3 y2 y1
LA
Y Q K S T E L L I R
a/b1 a/b3
CycIm for Kla at m/z 156.1019
y(1)
b(1)
y(2)
y(3)
yb-STEL
y(4)
b(3)
y(5)
y(6)
y(7)
y(8)
y°(9)
% of base peak
ion current
m/z
observed
0
20
40
60
80
100
0
200
400
600
800
1000
1200
0.0e+0
2.0e+4
4.0e+4
6.0e+4
8.0e+4
1.0e+5
1.2e+5

H3 K64LPFQR

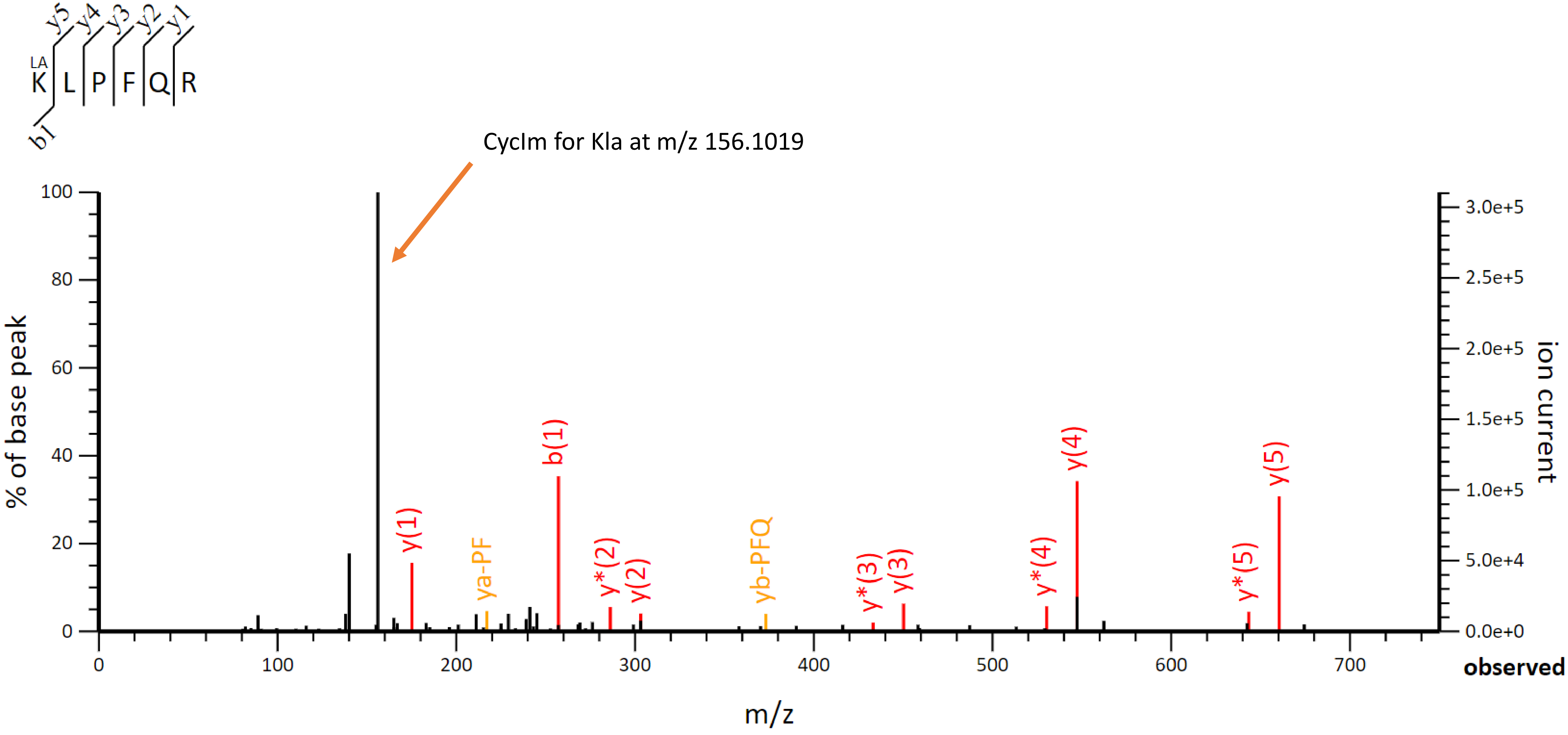

y5 y4 y3 y2 y1
LA
K L P F Q R
b1
CycIm for Kla at m/z 156.1019
% of base peak
ion current
m/z
observed
y(1)
ya-PF
b(1)
y*(2)
y(2)
yb-PFQ
y*(3)
y(3)
y*(4)
y(4)
y*(5)
y(5)

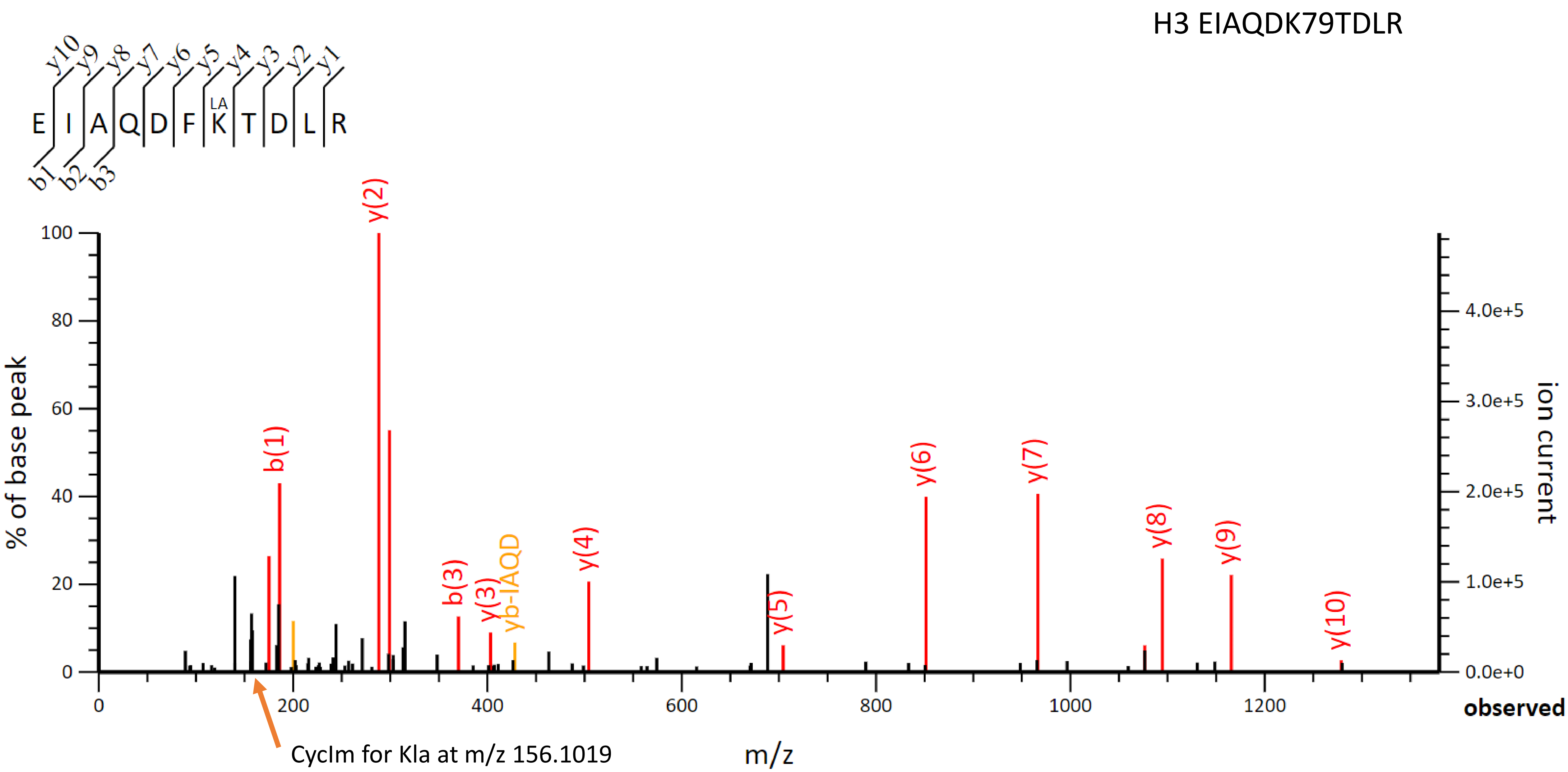

H3 EIAQDK79TDLR
E I A Q D F K T D L R
LA
y10 y9 y8 y7 y6 y5 y4 y3 y2 y1
b1 b2 b3
% of base peak
ion current
m/z
observed
y(2)
b(1)
b(3)
y(3)
yb-IAQD
y(4)
y(5)
y(6)
y(7)
y(8)
y(9)
y(10)
CycIm for Kla at m/z 156.1019

b1 ion for Valine is expected at mass 156.1019, that is the same m/z as for the CycIm of Klac.
We validated K122(lac) based on fragments y6 and y7.

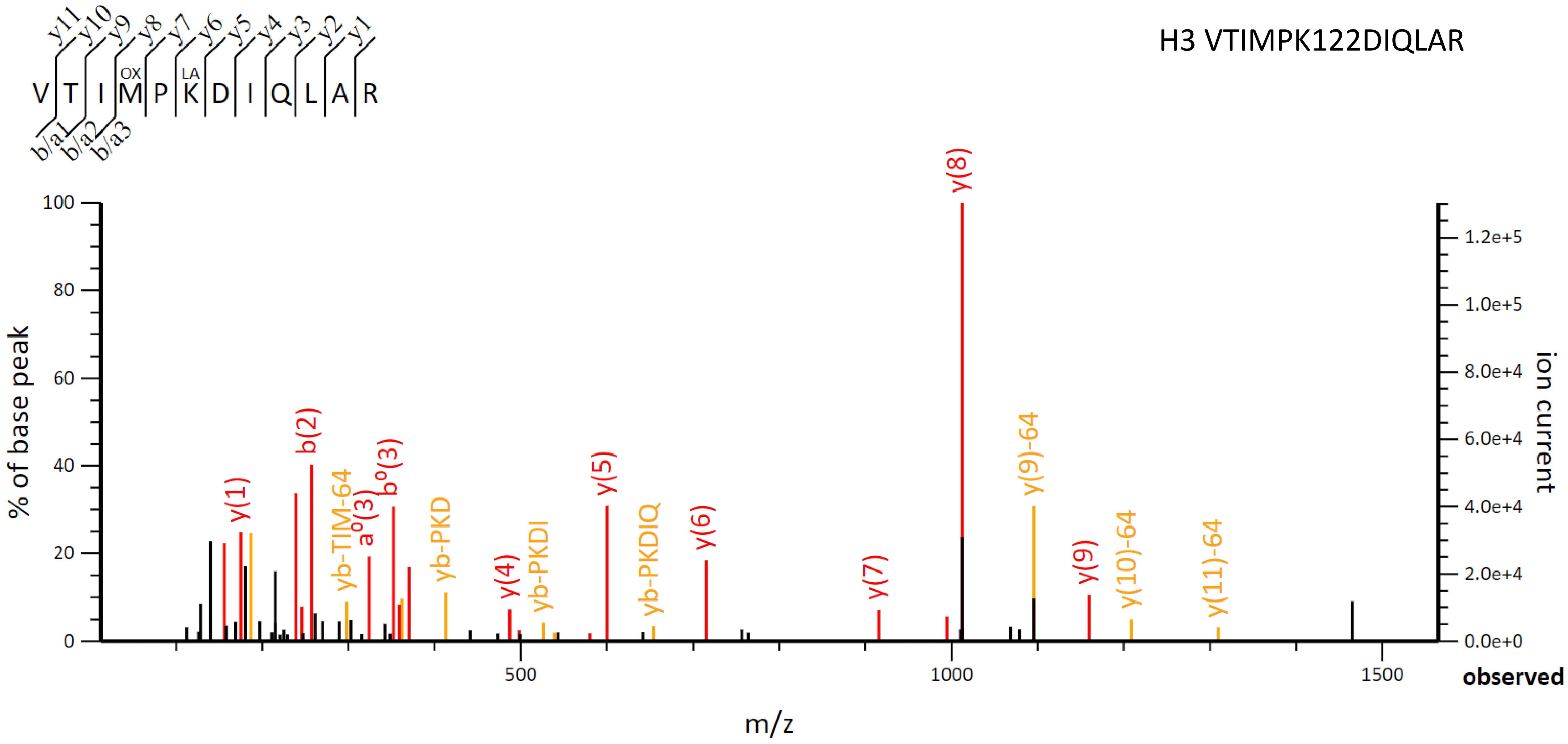

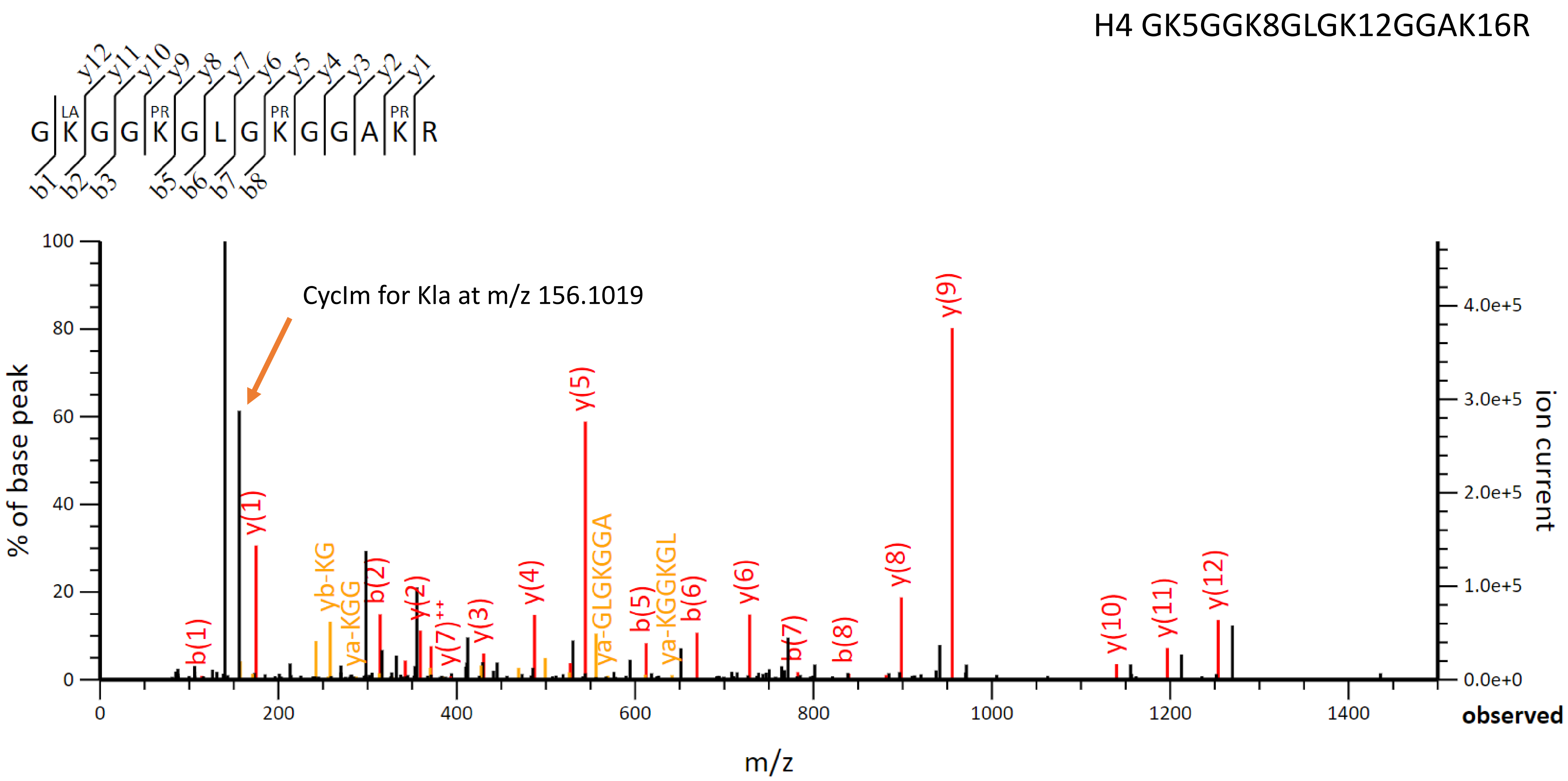

H4 GK5GGK8GLGK12GGAK16R
y12 y11 y10 y9 y8 y7 y6 y5 y4 y3 y2 y1
G K G G K G L G K G G A K R
LA PR PR PR
b1 b2 b3 b5 b6 b7 b8
CycIm for Kla at m/z 156.1019
% of base peak
ion current
m/z
observed
y(1) b(1) yb-KG ya-KGG b(2) y(2) y(7)++ y(3) y(4) y(5) ya-GLGKGGA b(5) ya-KGGKGL b(6) y(6) b(7) b(8) y(8) y(9) y(10) y(11) y(12)

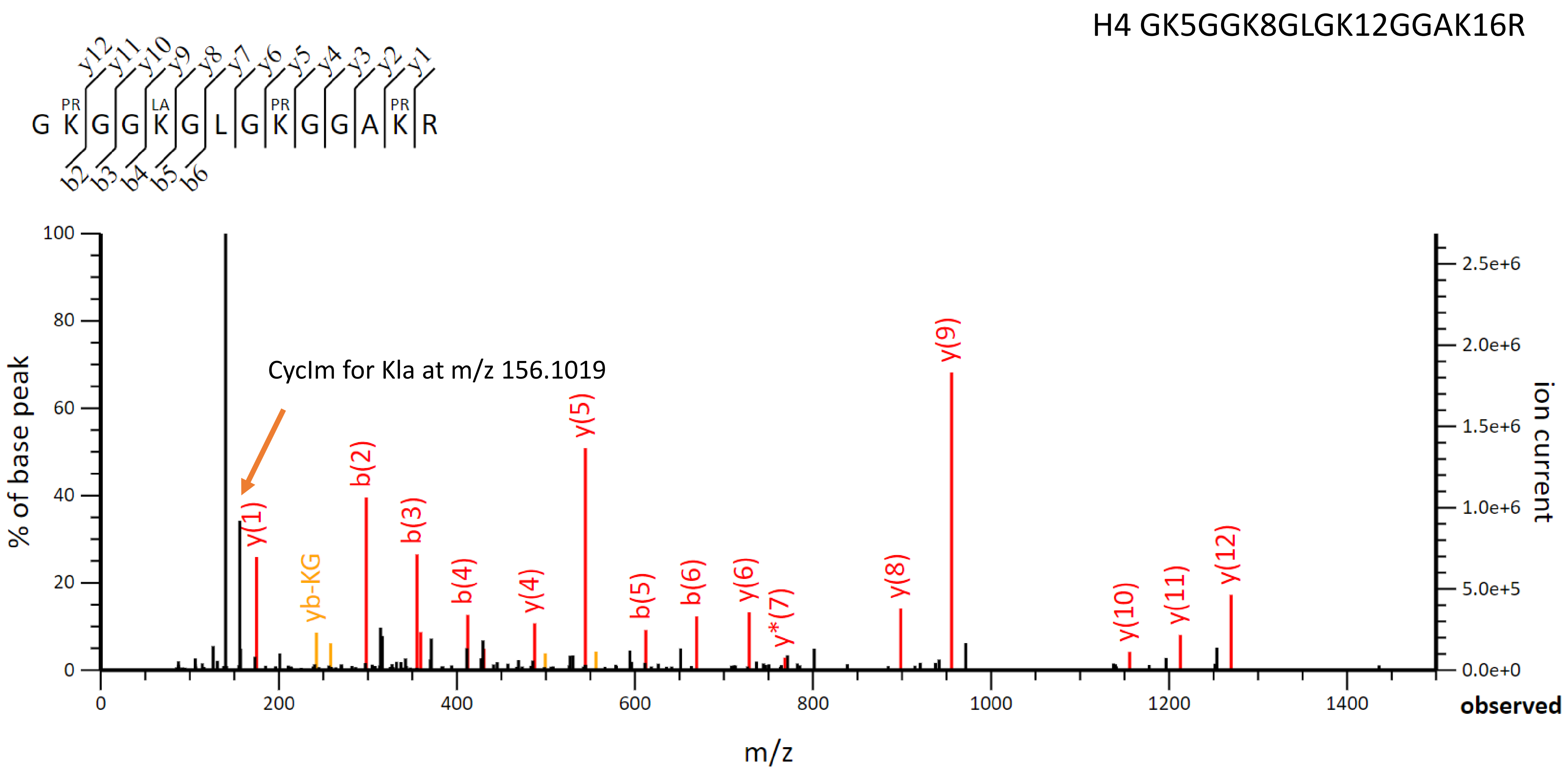
H4 GK5GGK8GLGK12GGAK16R
y12 y11 y10 y9 y8 y7 y6 y5 y4 y3 y2 y1
G K G G K G L G K G G A K R
PR LA PR PR
b2 b3 b4 b5 b6
Cyclm for Kla at m/z 156.1019
% of base peak
ion current
m/z
observed
y(1) yb-KG b(2) b(3) b(4) y(4) y(5) b(5) b(6) y(6) y*(7) y(8) y(9) y(10) y(11) y(12)

H4 GK5GGK8GLGK12GGAK16R

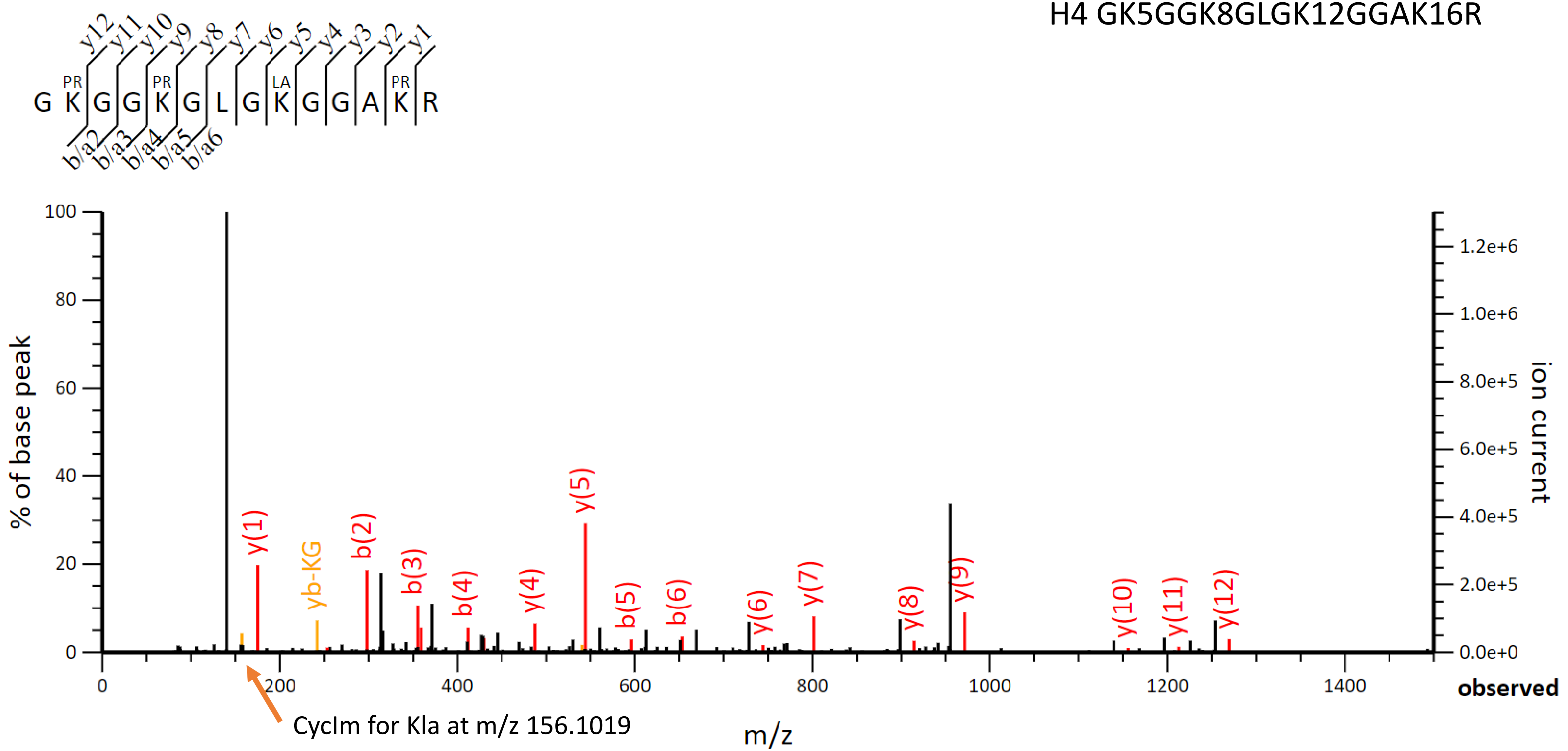

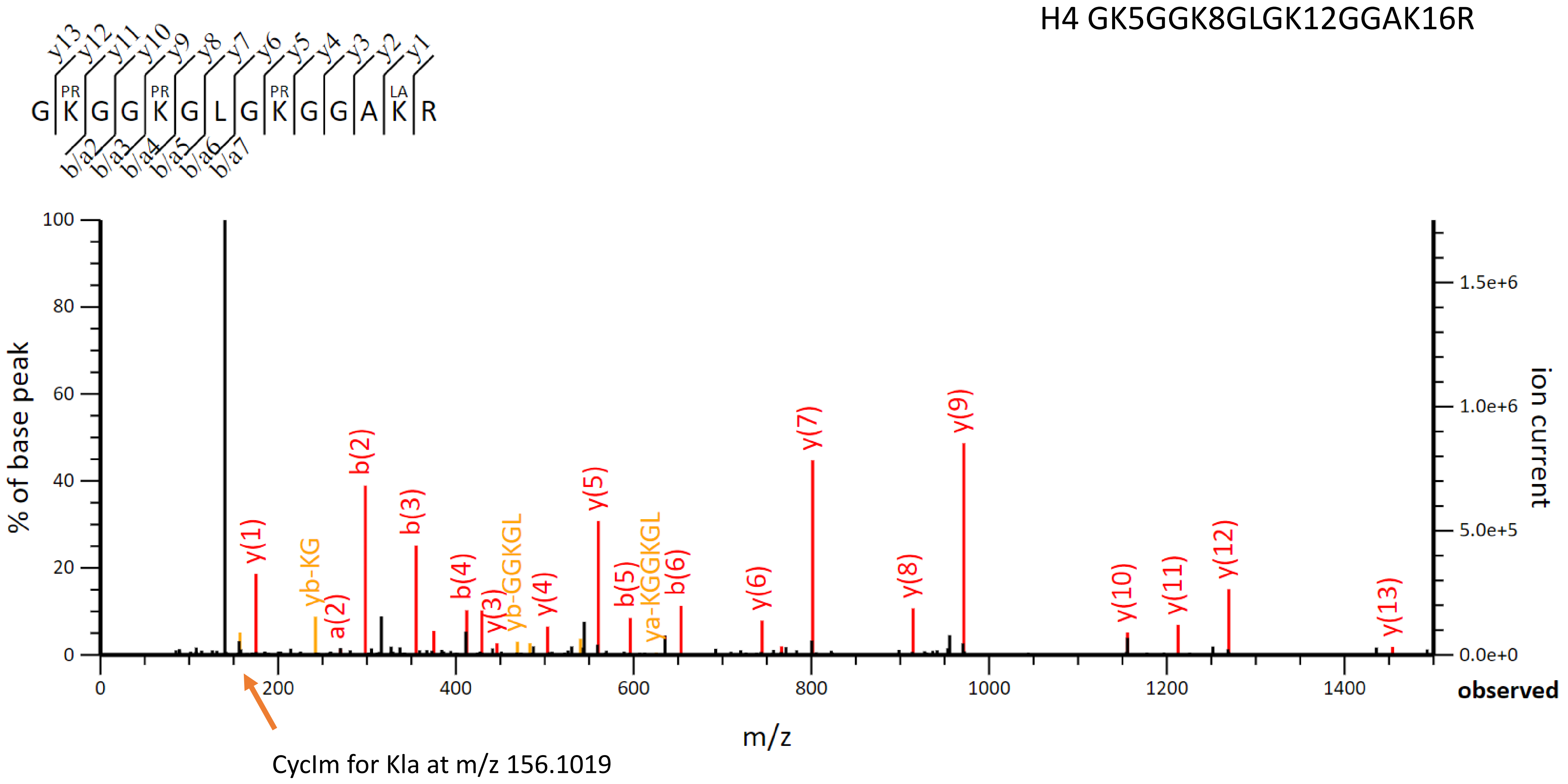

H4 GK5GGK8GLGK12GGAK16R
G K G G K G L G K G G A K R
PR
PR
PR
LA
y13 y12 y11 y10 y9 y8 y7 y6 y5 y4 y3 y2 y1
b/a2 b/a3 b/a4 b/a5 b/a6 b/a7
% of base peak
ion current
m/z
observed
y(1)
yb-KG
a(2)
b(2)
b(3)
b(4)
y(3)
yb-GGKGL
y(4)
y(5)
b(5)
ya-KGGKGL
b(6)
y(6)
y(7)
y(8)
y(9)
y(10)
y(11)
y(12)
y(13)
CycIm for Kla at m/z 156.1019

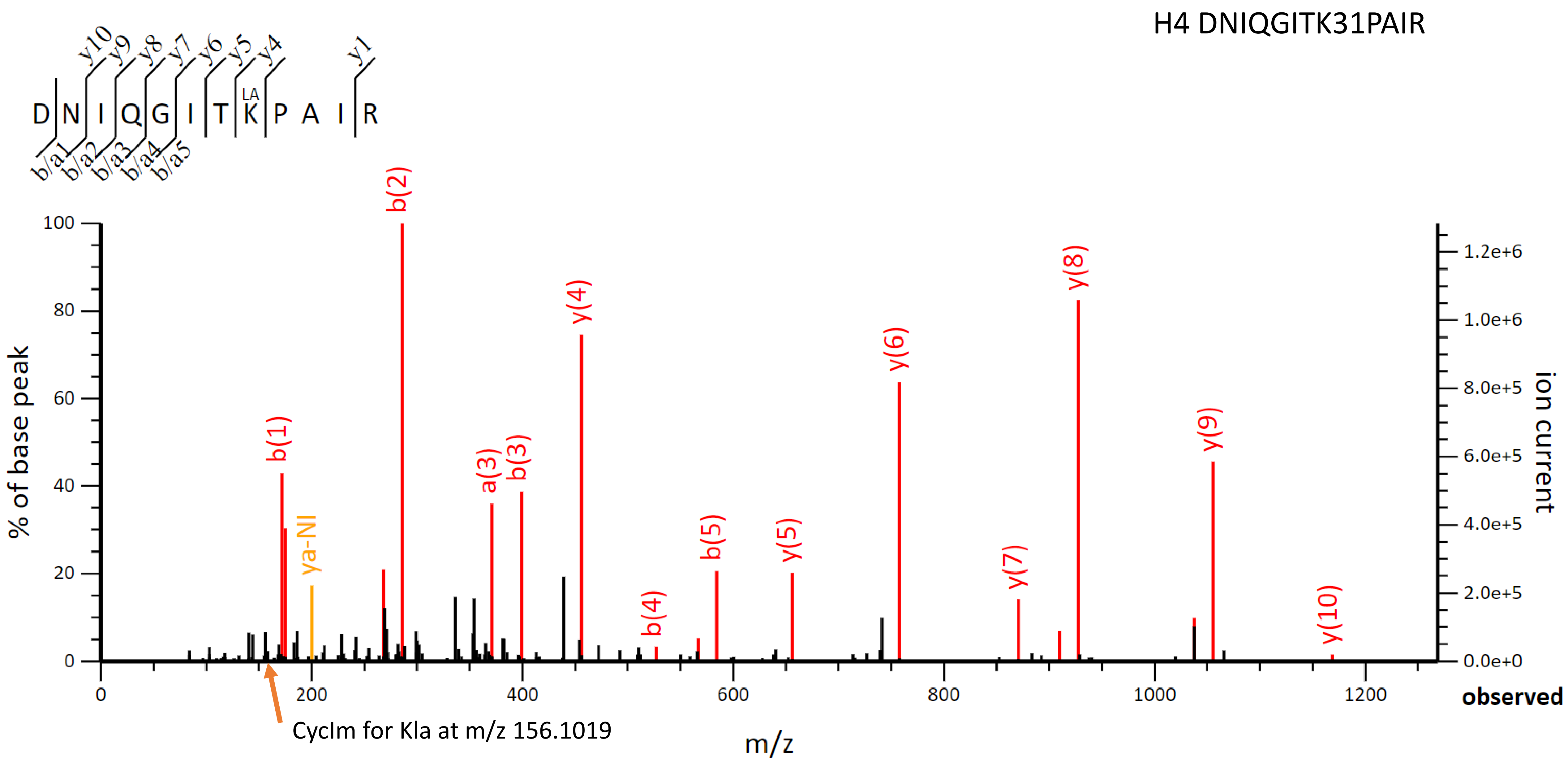
H4 DNIQGITK31PAIR
D N I Q G I T K P A I R
LA
y10 y9 y8 y7 y6 y5 y4 y1
b/a1 b/a2 b/a3 b/a4 b/a5
% of base peak
ion current
m/z
observed
b(1) b(2) a(3) b(3) y(4) b(4) b(5) y(5) y(6) y(7) y(8) y(9) y(10)
ya-NI
CycIm for Kla at m/z 156.1019

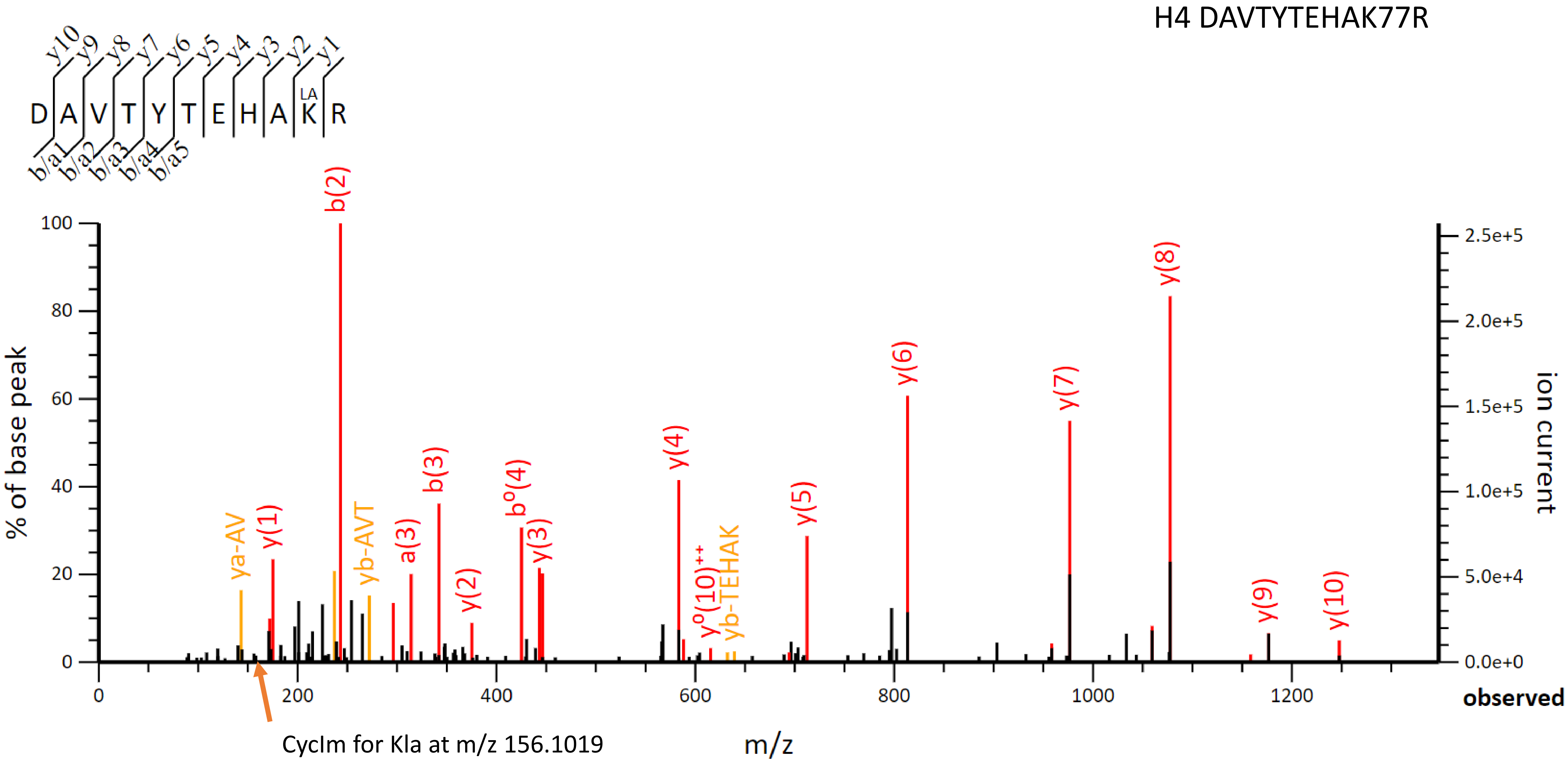
H4 DAVTYTEHAK77R
D A V T Y T E H A K R
LA
y10 y9 y8 y7 y6 y5 y4 y3 y2 y1
b/a1 b/a2 b/a3 b/a4 b/a5
b(2)
y(8)
y(6)
y(7)
y(4)
b(3)
bº(4)
y(5)
y(3)
a(3)
y(1)
ya-AV
yb-AVT
y(2)
yº(10)++
yb-TEHAK
y(9)
y(10)
% of base peak
ion current
100
80
60
40
20
0
2.5e+5
2.0e+5
1.5e+5
1.0e+5
5.0e+4
0.0e+0
0
200
400
600
800
1000
1200
observed
m/z
CycIm for Kla at m/z 156.1019

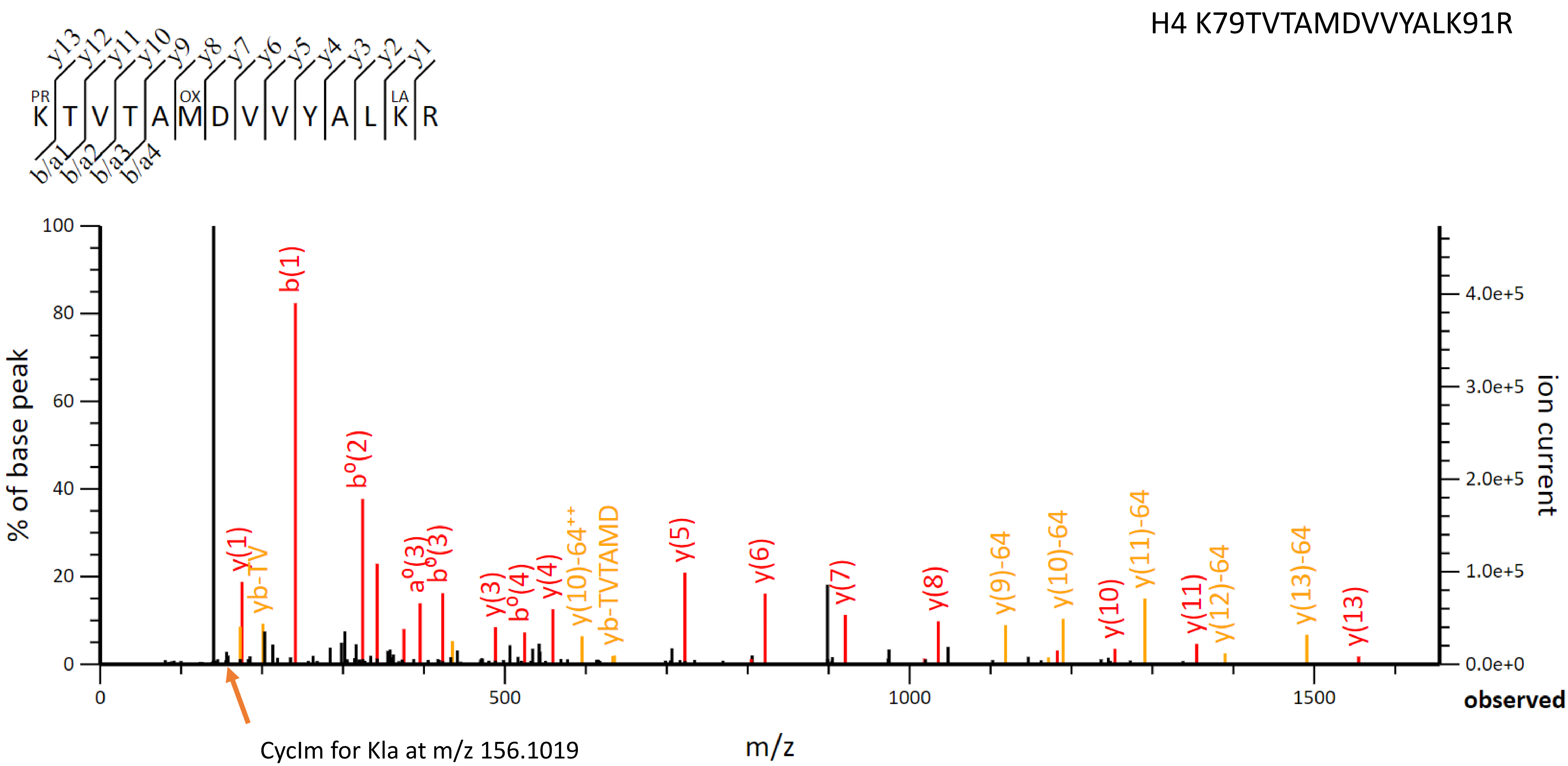

H4 K79TVTAMDVVYALK91R
PR
OX
LA
K T V T A M D V V Y A L K R
y13 y12 y11 y10 y9 y8 y7 y6 y5 y4 y3 y2 y1
b/a1 b/a2 b/a3 b/a4
% of base peak
ion current
m/z
observed
CycIm for Kla at m/z 156.1019

Section 2:

False identifications of presumably lactylated peptides correspond to propionylated and oxidized peptides, which can be of prevailing abundance

Oxidation events on histone H4 DNIQGITK$_{31}$PAIR peptide, m/z =727.4041,2+

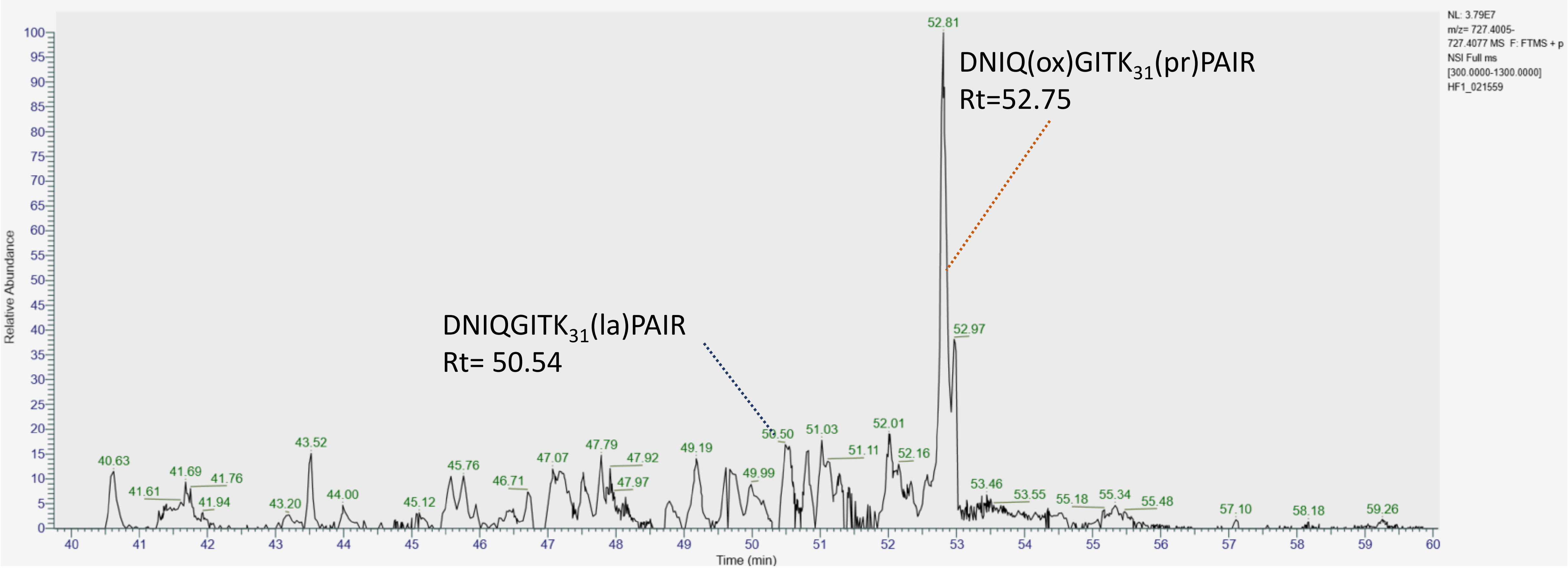

Scan 24196 , rt=52.751, HF1_021559.raw, 171924.dat

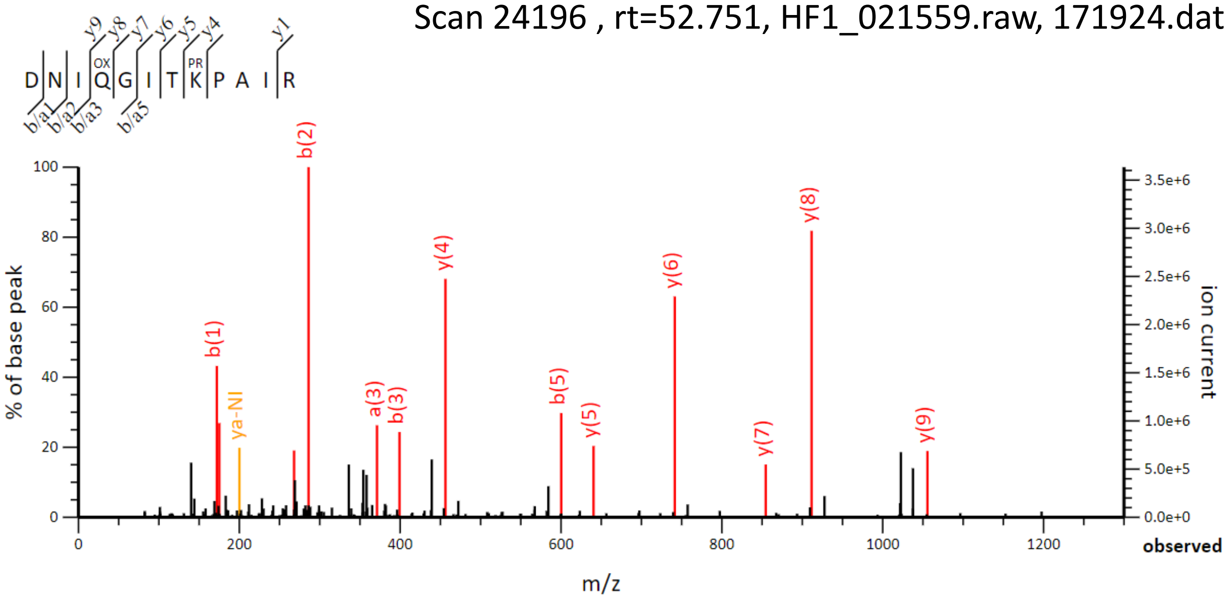

Scan 23089 , rt=50.537, HF1_021559.raw, 171924.dat

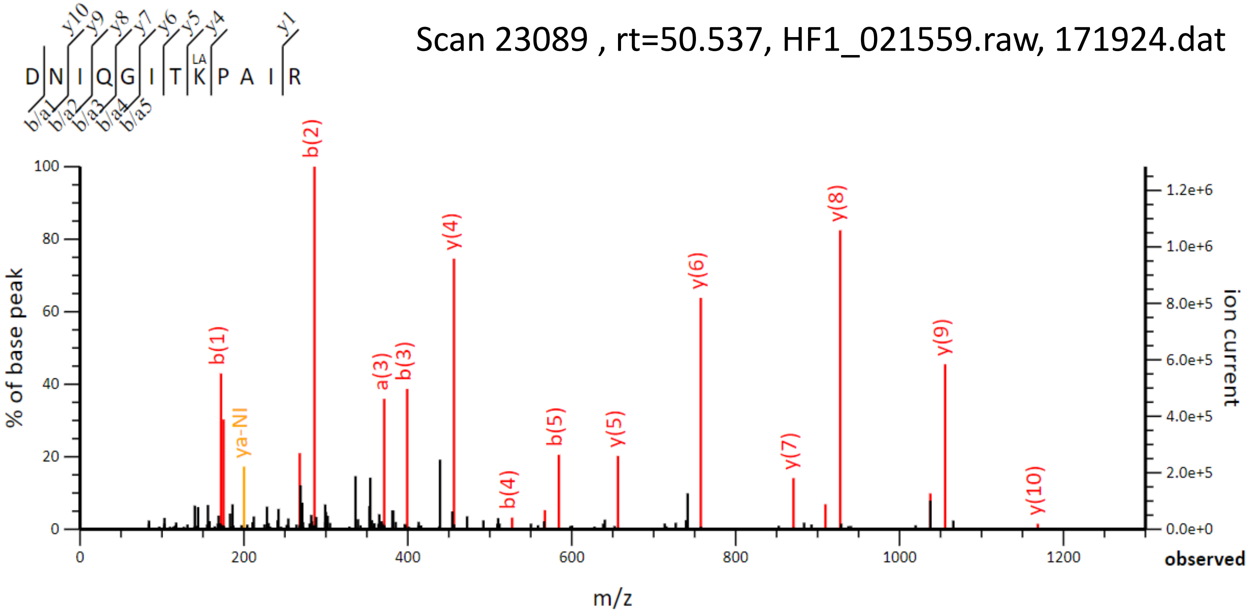

Oxidation events on histone H4 DAVTYTEHK$_{77}$R peptide, m/z = 709.8491 (2+)

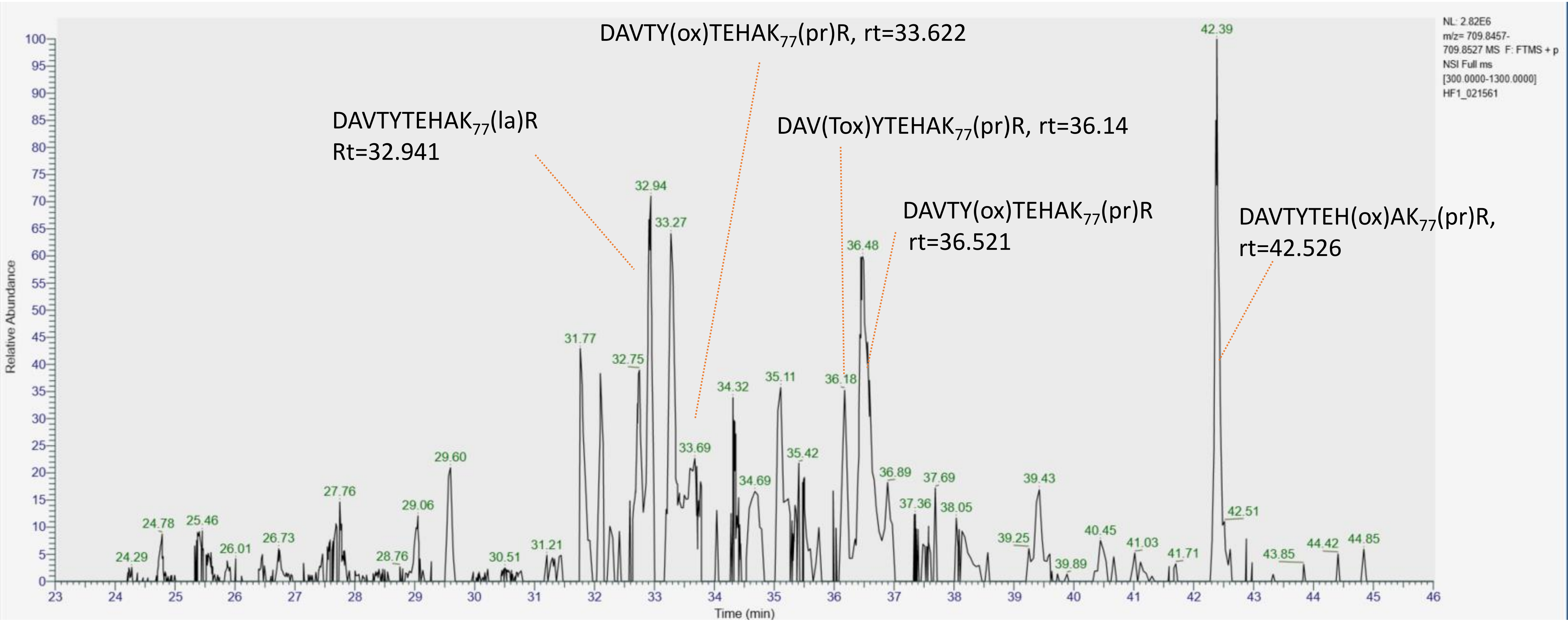

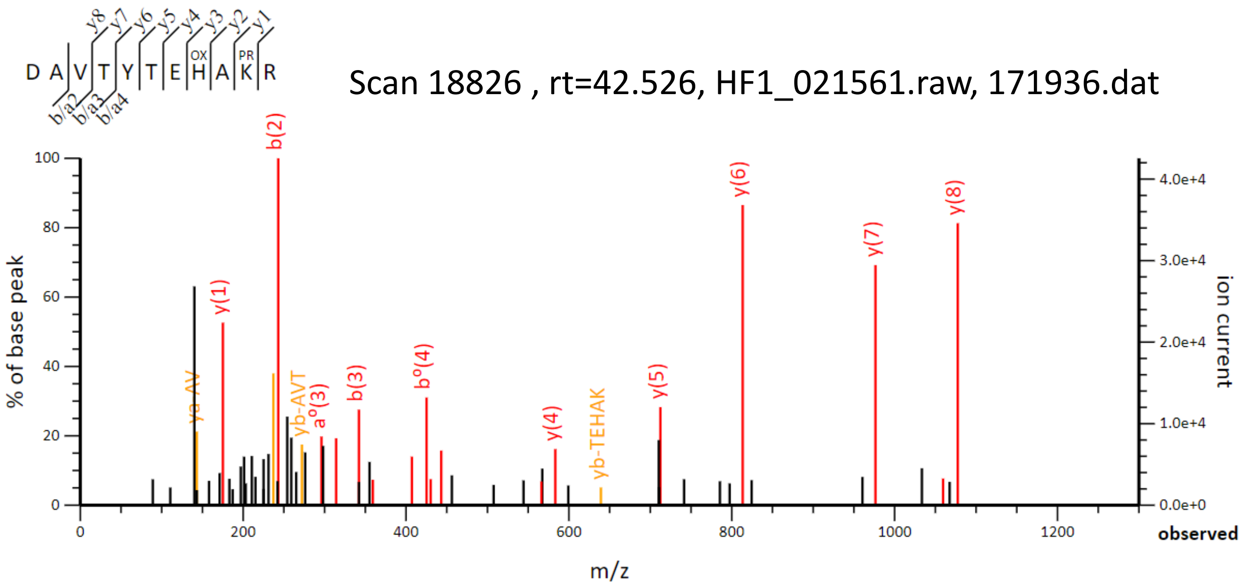
Scan 18826 , rt=42.526, HF1_021561.raw, 171936.dat
D A V T Y T E H A K R
% of base peak
ion current
m/z
observed

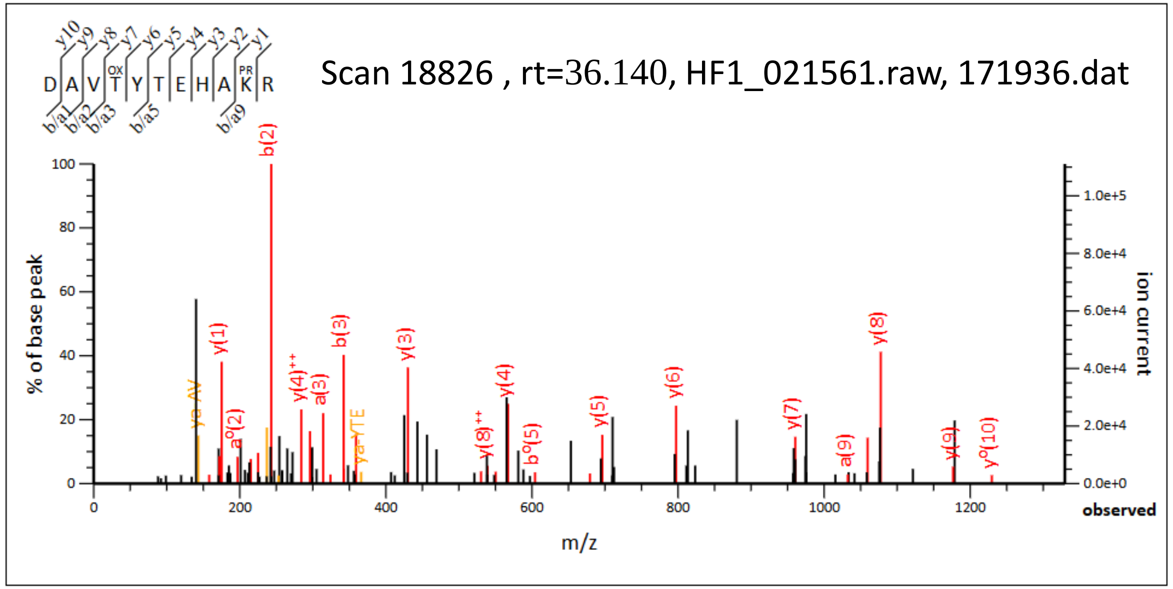
Scan 18826 , rt=36.140, HF1_021561.raw, 171936.dat
D A V T Y T E H A K R
% of base peak
ion current
m/z
observed

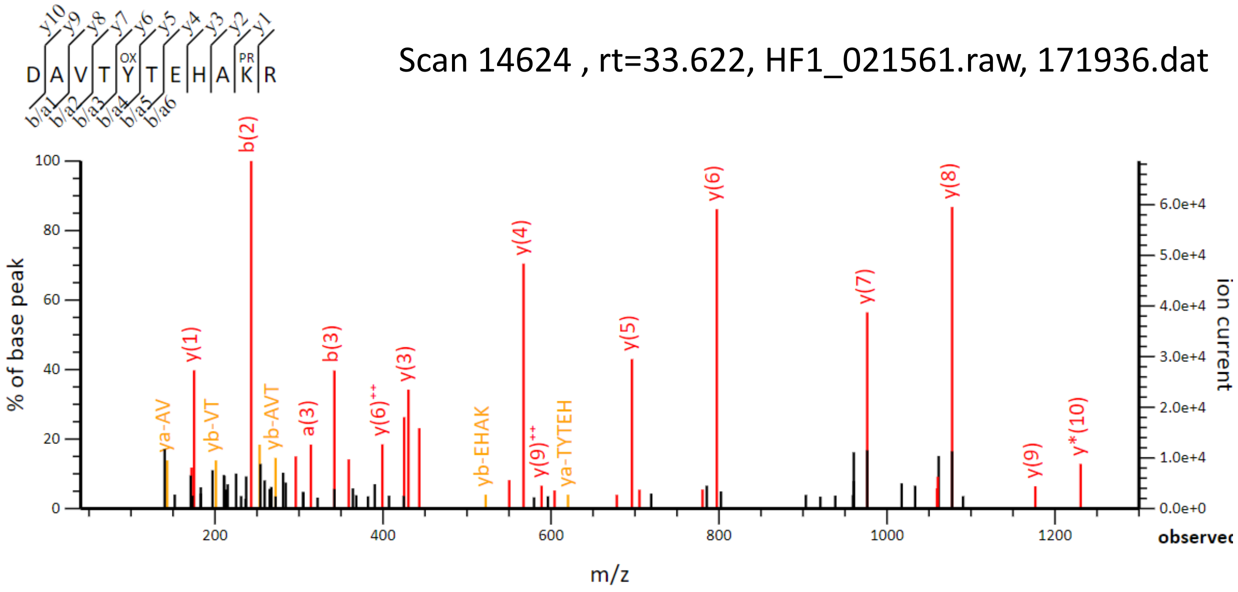
Scan 14624 , rt=33.622, HF1_021561.raw, 171936.dat
D A V T Y T E H A K R
% of base peak
ion current
m/z
observed

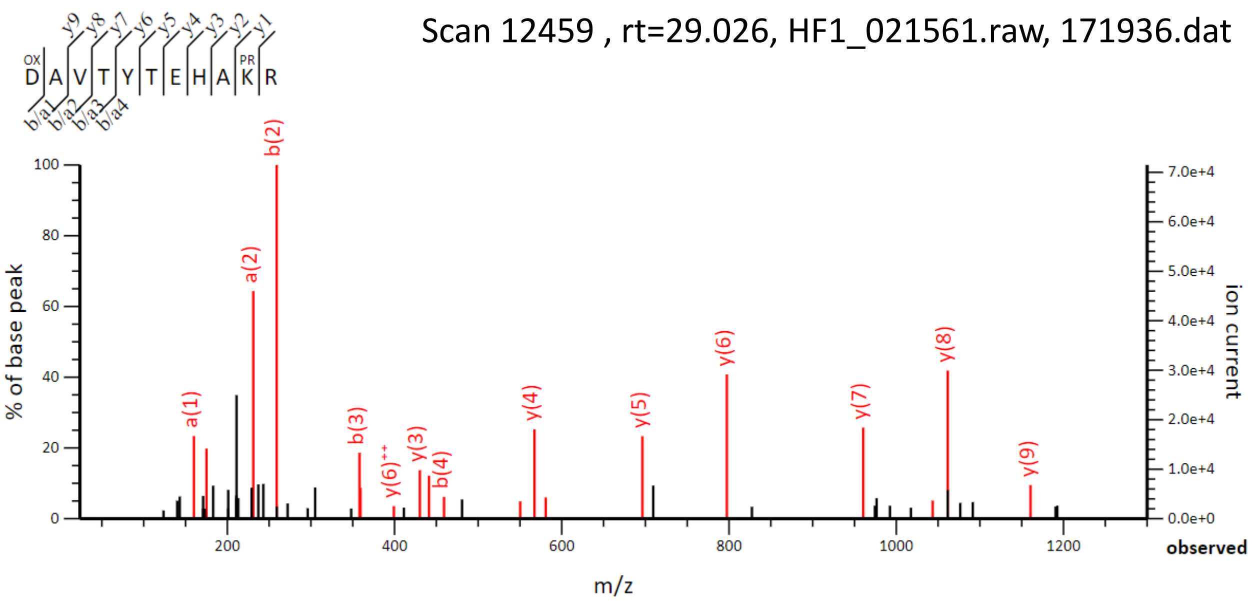
Scan 12459 , rt=29.026, HF1_021561.raw, 171936.dat
D A V T Y T E H A K R
% of base peak
ion current
m/z
observed

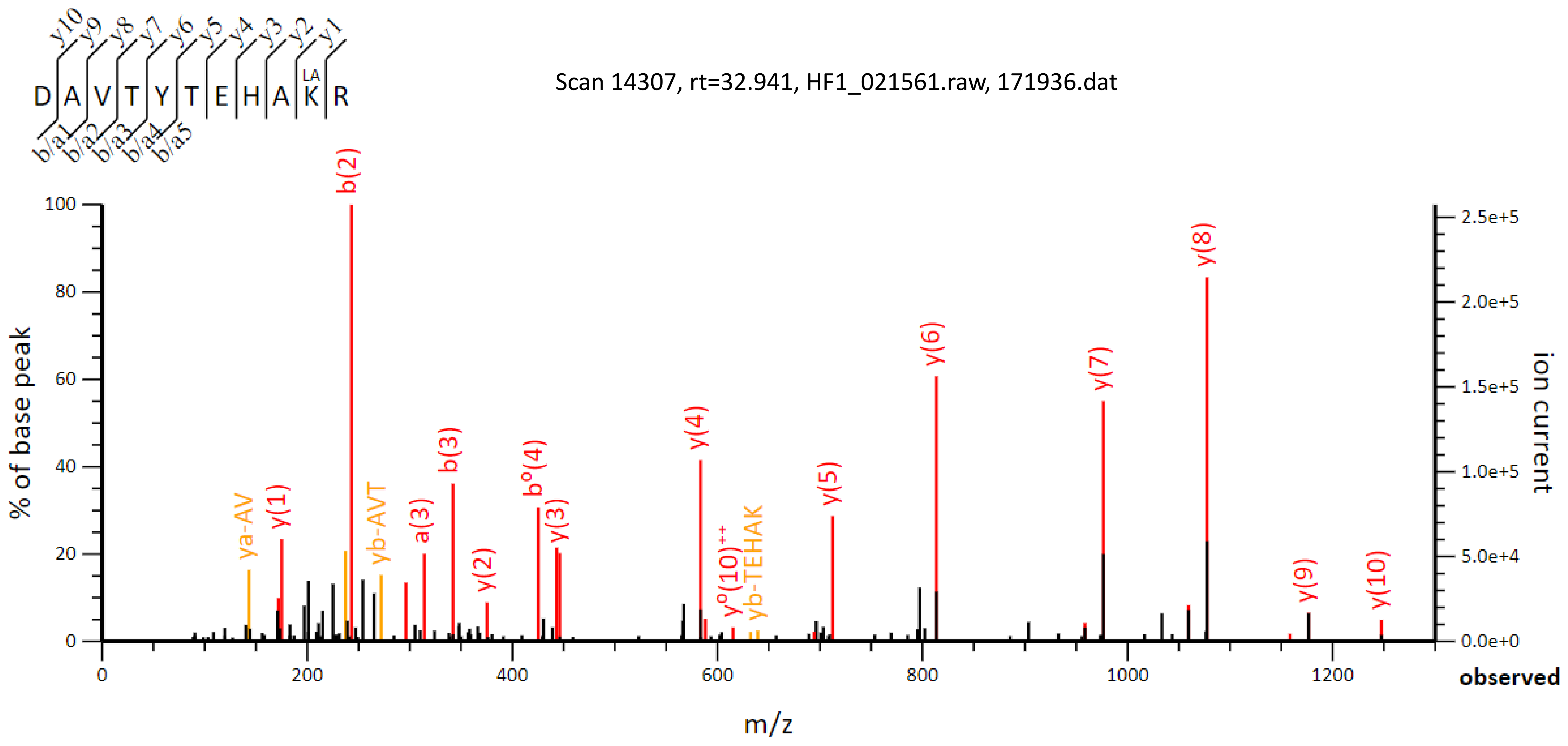

Scan 14307, rt=32.941, HF1_021561.raw, 171936.dat
D A V T Y T E H A K R
LA
y10 y9 y8 y7 y6 y5 y4 y3 y2 y1
b/a1 b/a2 b/a3 b/a4 b/a5
% of base peak
ion current
m/z
observed
b(2)
y(8)
y(6)
y(7)
y(4)
b(3)
b°(4)
y(5)
y(1)
ya-AV
yb-AVT
a(3)
y(3)
y(2)
y°(10)++
yb-TEHAK
y(9)
y(10)

Oxidation events on histone H4 K_{79}TVTAMDVVYALK_{91}R peptide, m/z=897.9871 (2+)

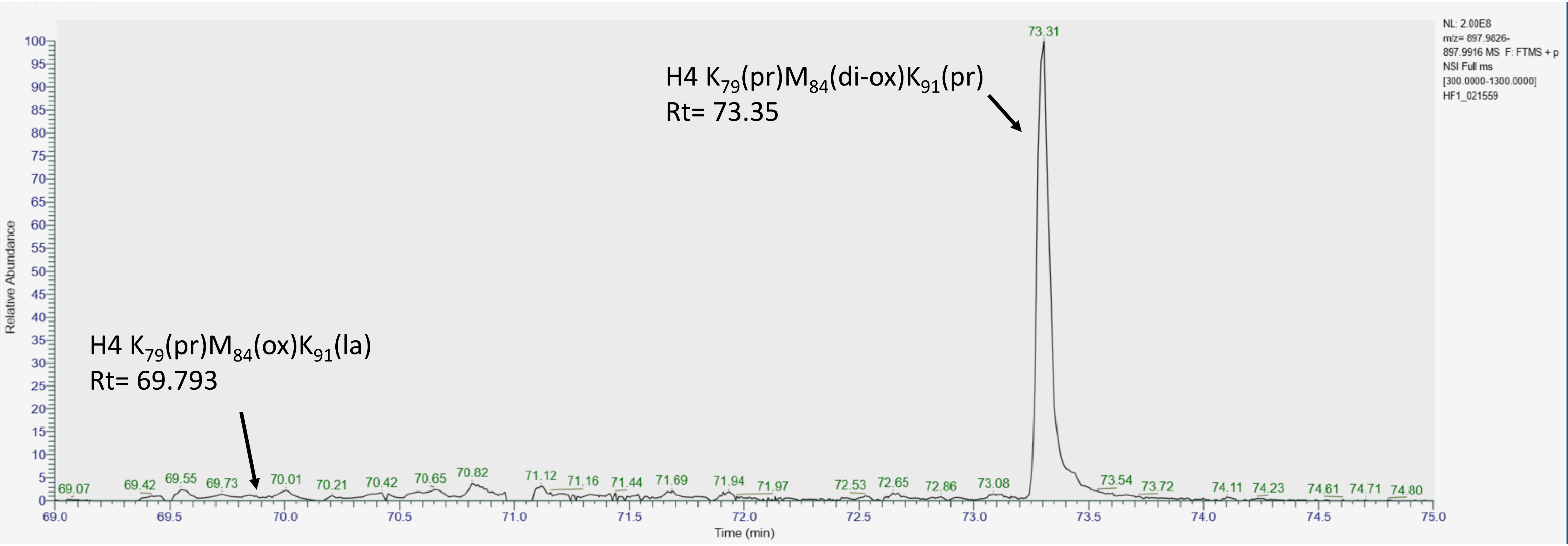

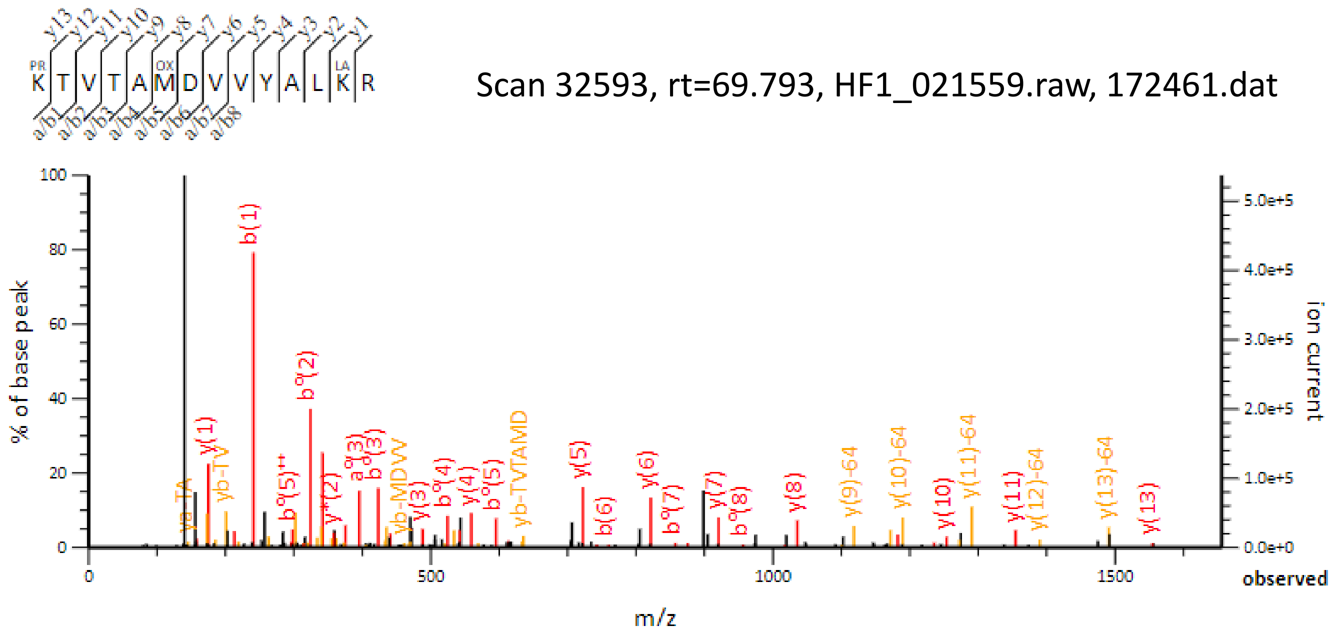

Scan 32593, rt=69.793, HF1_021559.raw, 172461.dat

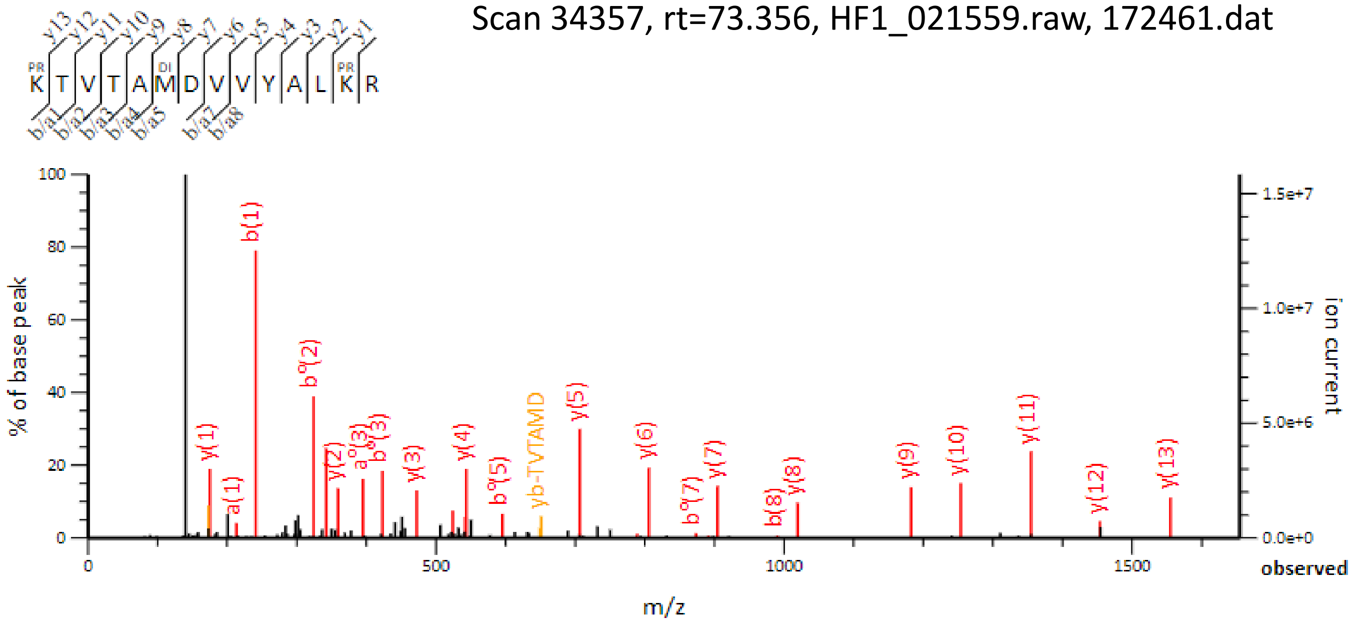

Scan 34357, rt=73.356, HF1_021559.raw, 172461.dat

Oxidation events on histone H3 YQK$_{56}$STELLIR peptide, m/z=689.8825 (2+)

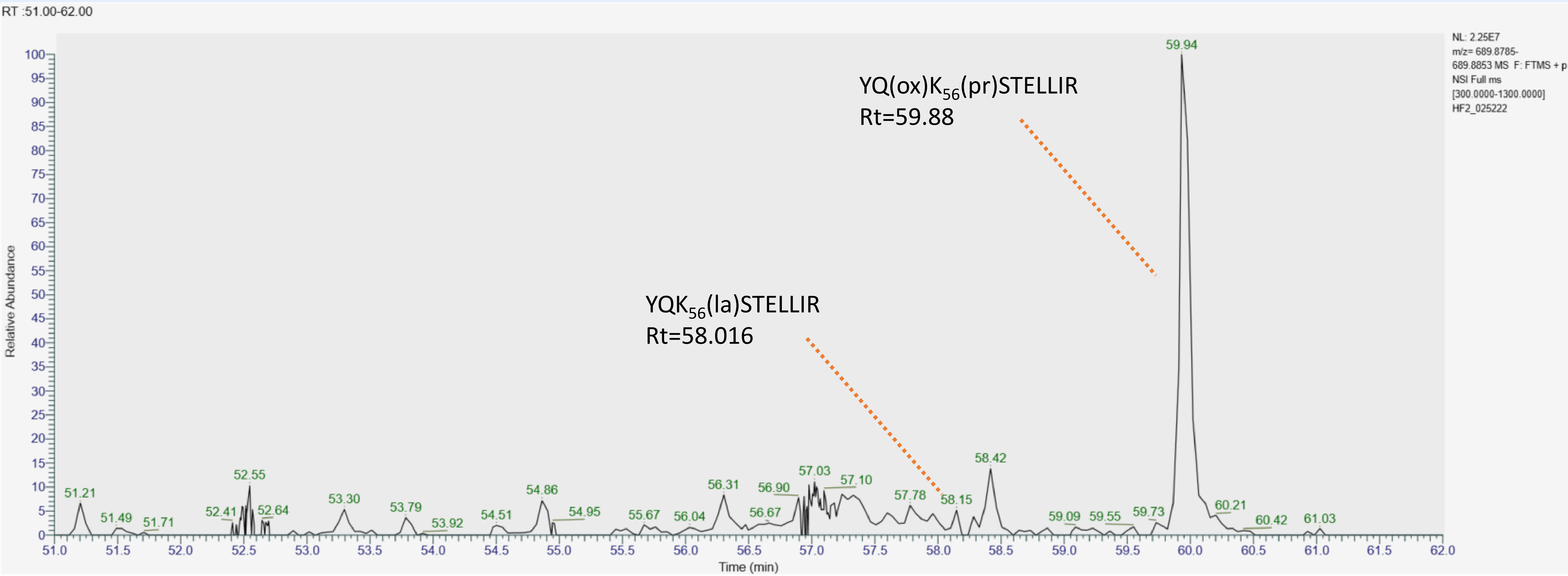

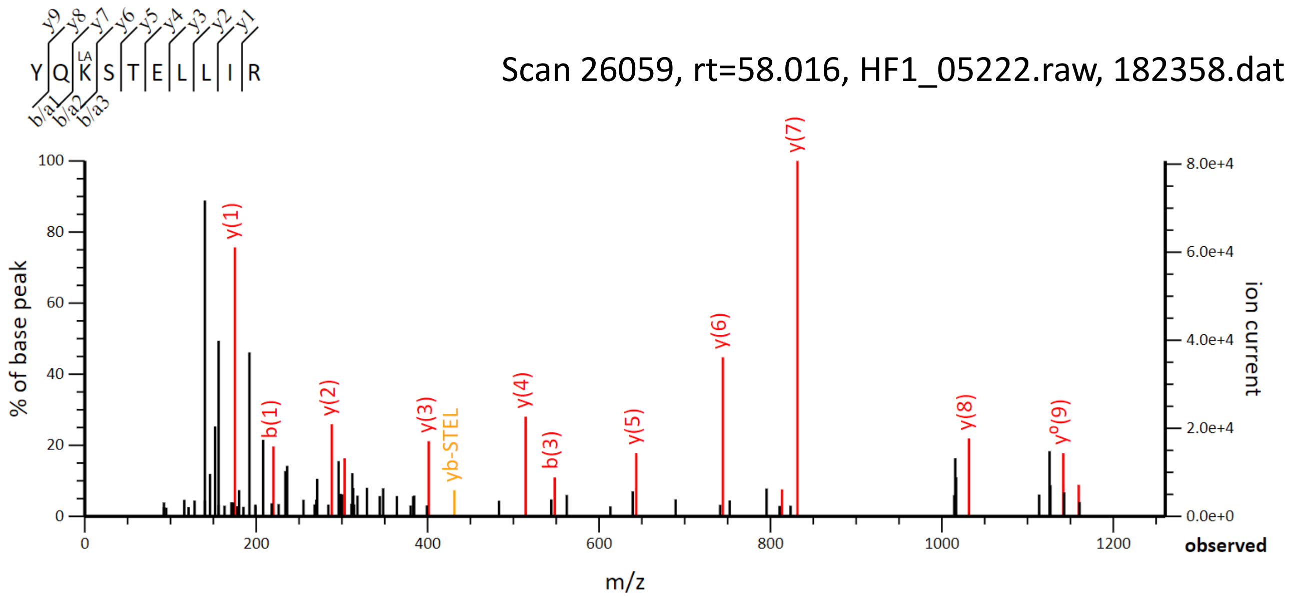
Scan 26059, rt=58.016, HF1_05222.raw, 182358.dat
Y Q K S T E L L I R
LA
y9 y8 y7 y6 y5 y4 y3 y2 y1
b/a1 b/a2 b/a3
% of base peak
ion current
m/z
observed
y(1)
b(1)
y(2)
y(3)
yb-STEL
y(4)
b(3)
y(5)
y(6)
y(7)
y(8)
y°(9)

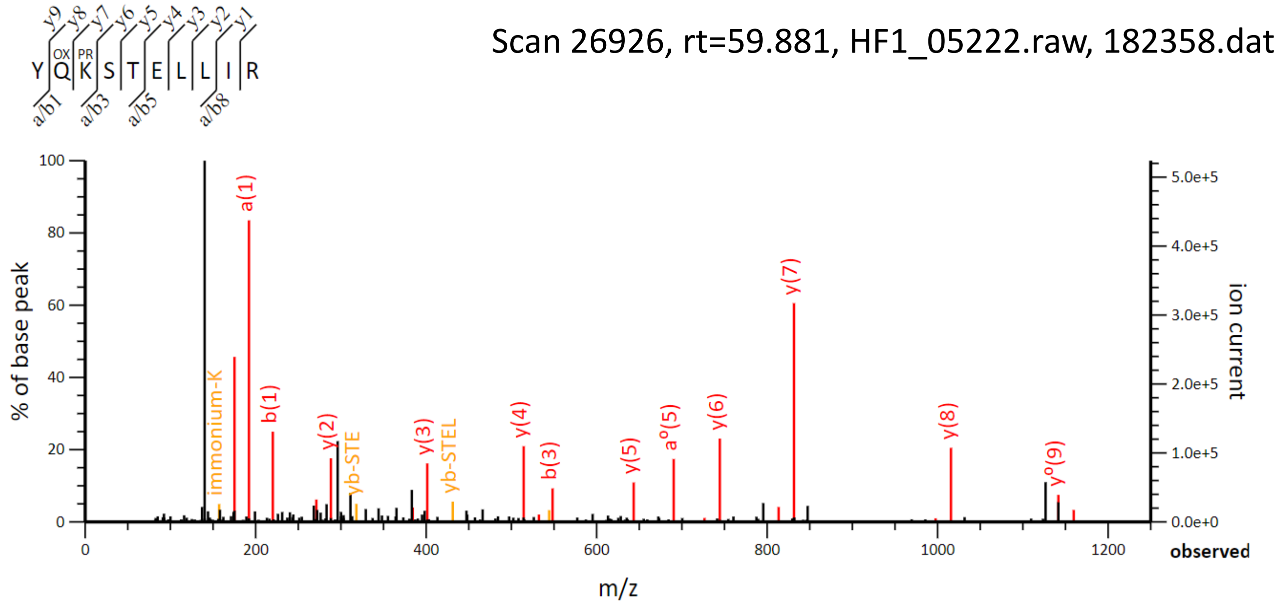
Scan 26926, rt=59.881, HF1_05222.raw, 182358.dat
Y Q K S T E L L I R
OX PR
y9 y8 y7 y6 y5 y4 y3 y2 y1
a/b1 a/b3 a/b5 a/b8
% of base peak
ion current
m/z
observed
immonium-K
a(1)
b(1)
y(2)
yb-STE
y(3)
yb-STEL
y(4)
b(3)
y(5)
a°(5)
y(6)
y(7)
y(8)
y°(9)

Section 3:

Determination of the chromatographic behavior of synthetic peptides from histones H3 and H4 bearing L- or D-lactylated lysines

Extracted ion chromatogram of H3.1 L- and D-lactyl $K_9STGGK_{14}APR$ (m/z=548.3052,2+)

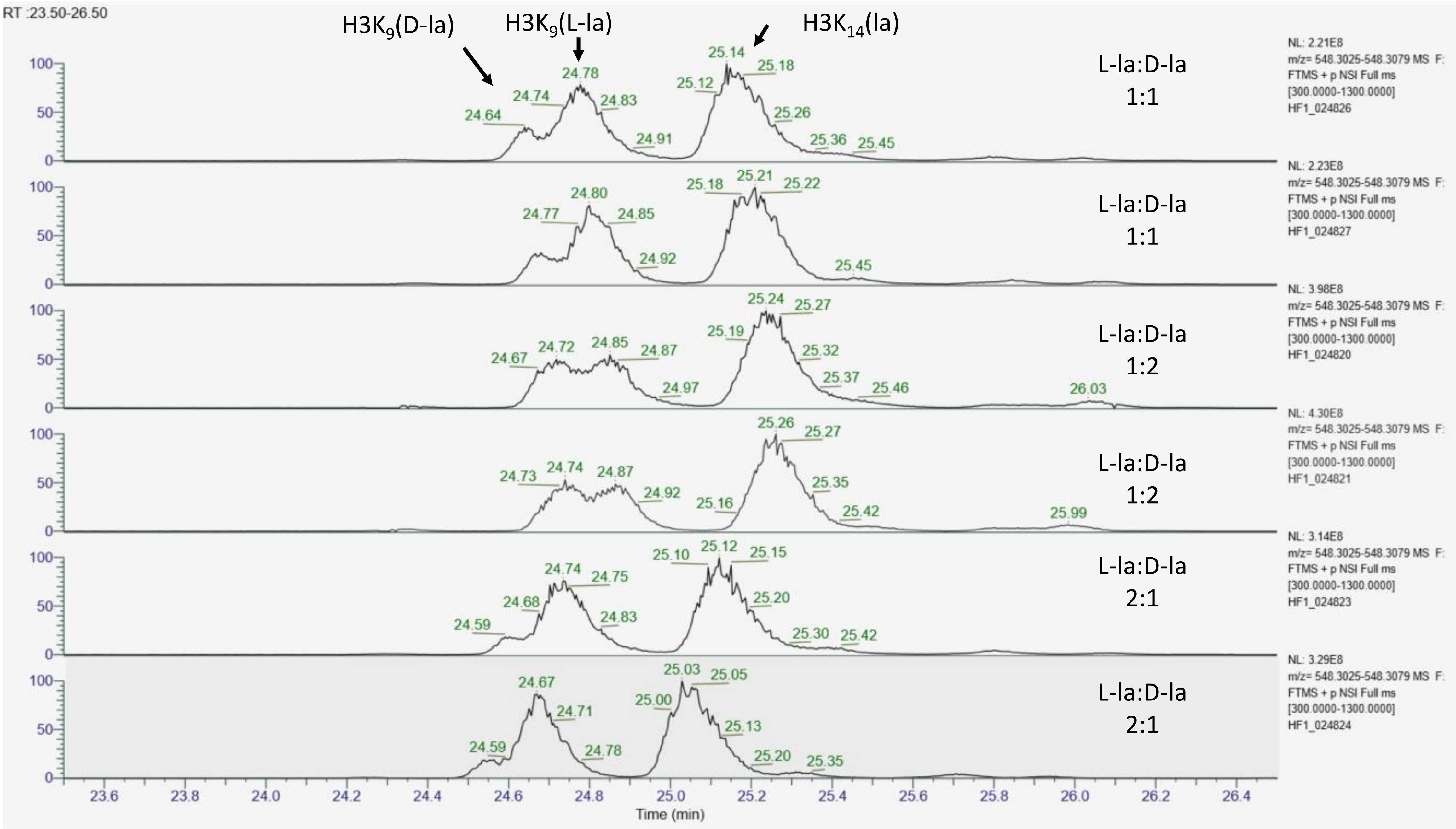

MS/MS spectra for H3 L- and D-lactyl $K_9STGGK_{14}APR$ in three different ratios

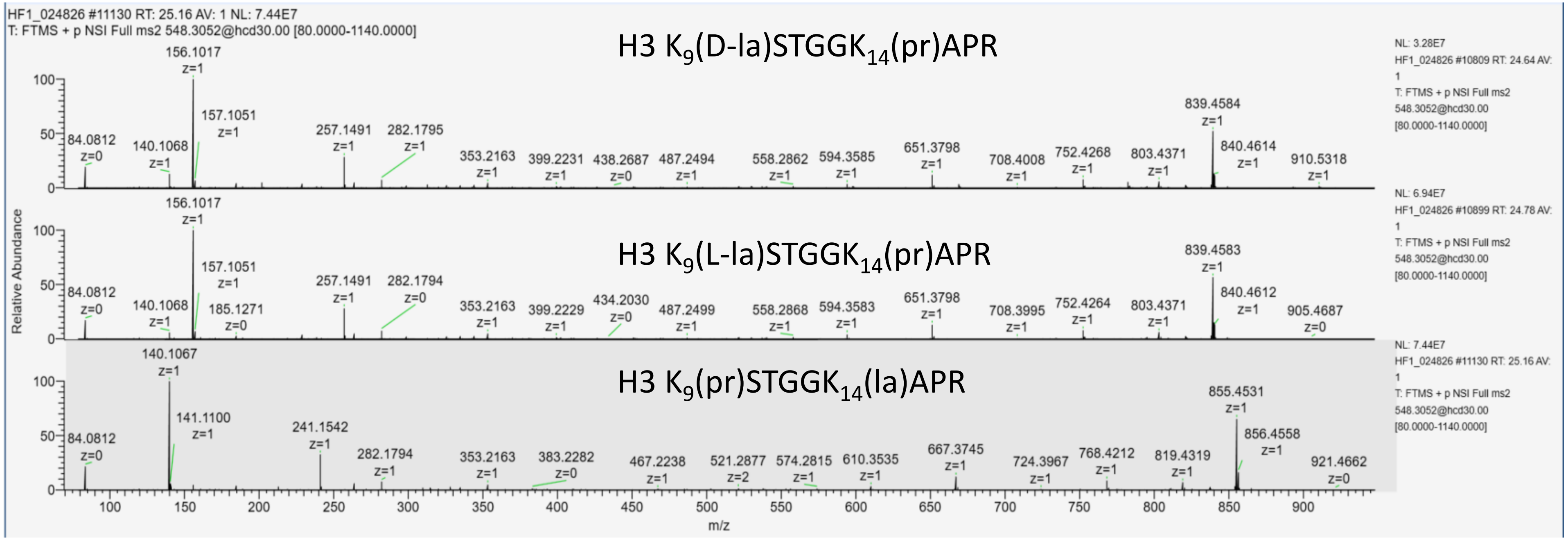

L-la:D-la
1:1

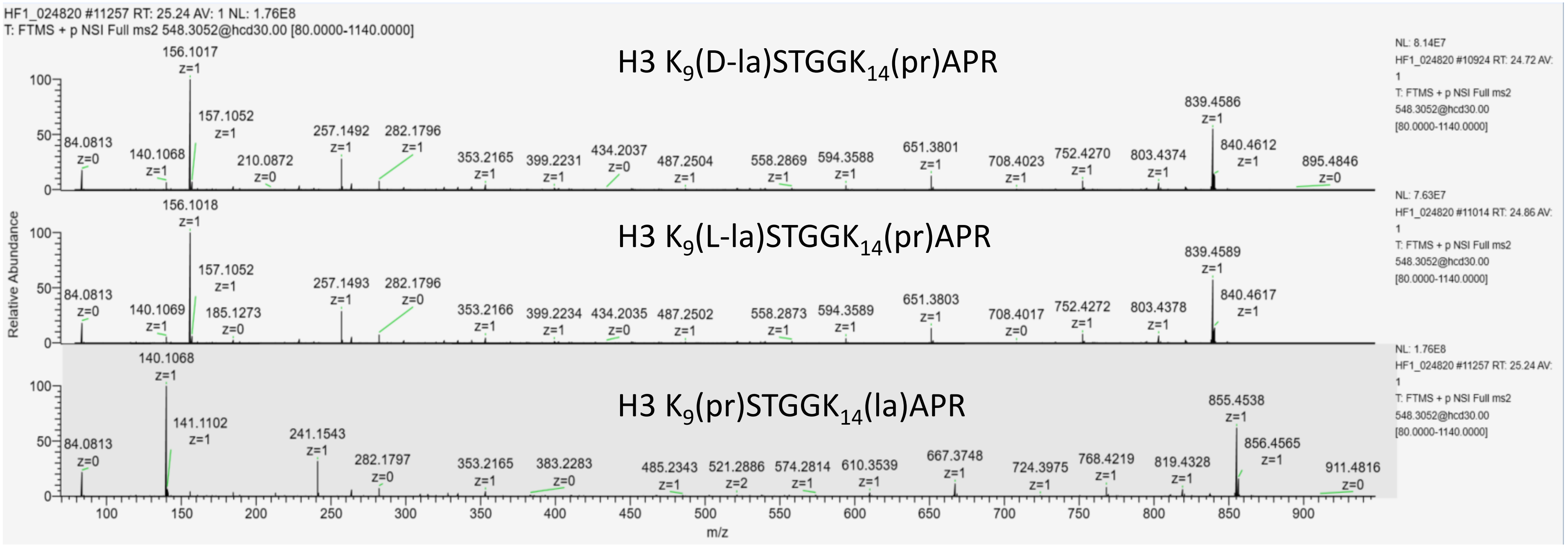

L-la:D-la
1:2

MS/MS spectra for H3 L- and D-lactyl $K_9STGGK_{14}APR$ in three different ratios

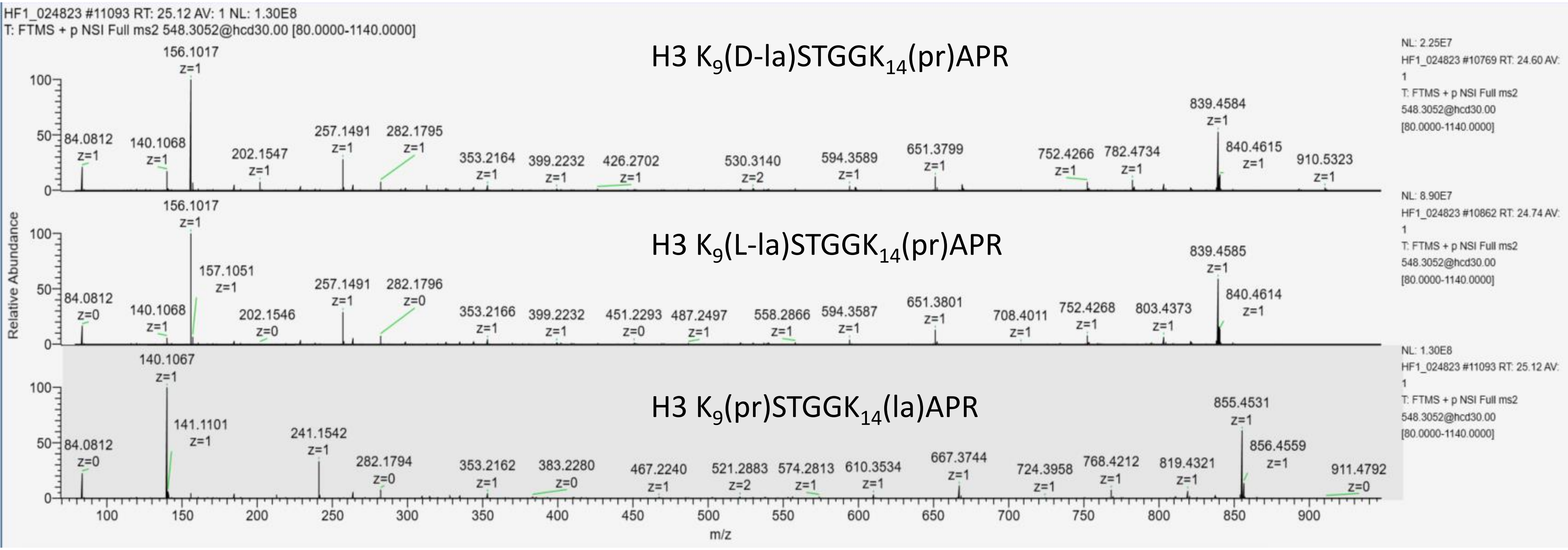

L-la:D-la
2:1

Extracted ion chromatogram of H3.1 L- and D-lactyl $K_{18}QLATK_{23}AAR$ (m/z=590.8498,2+)

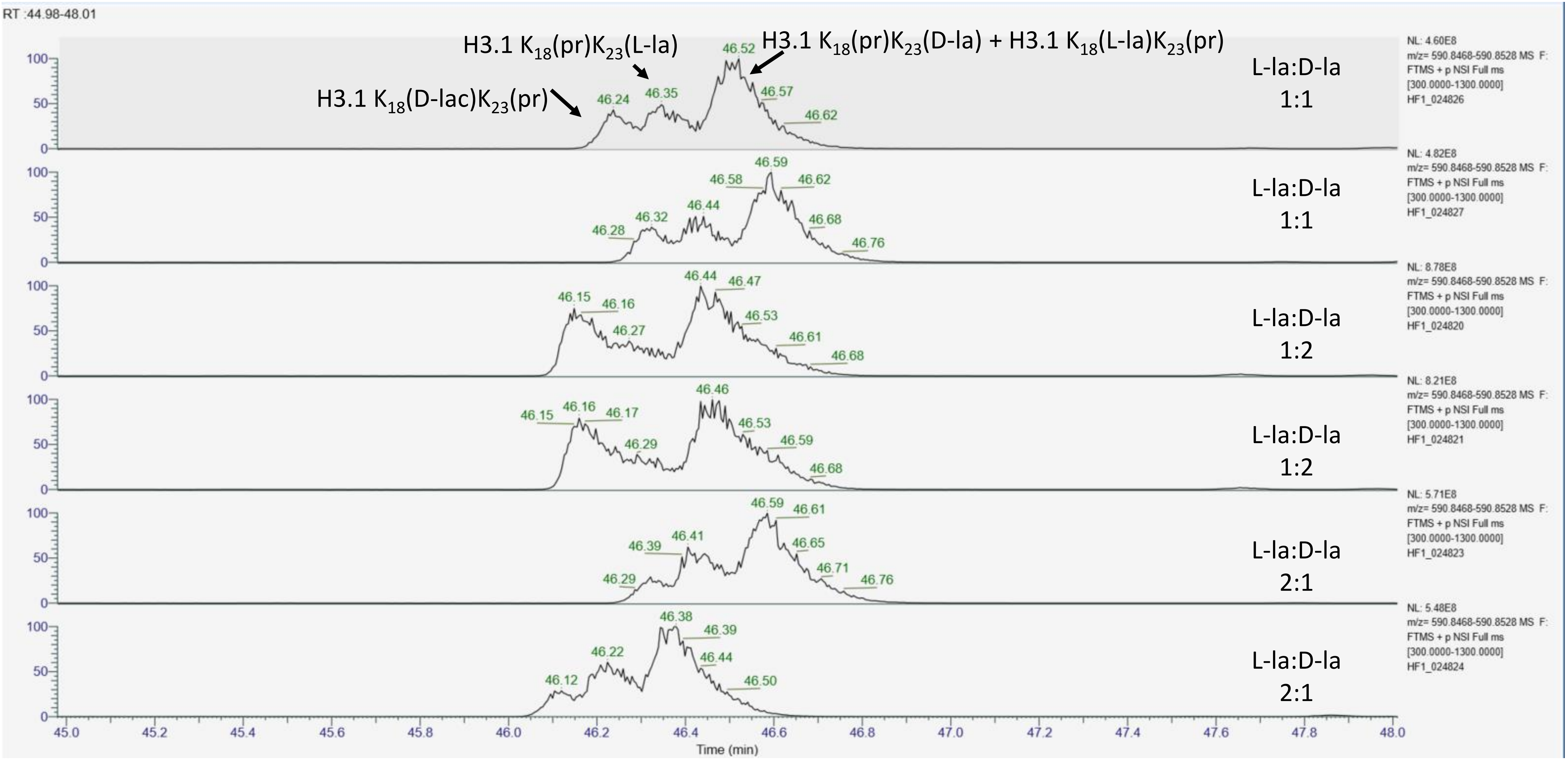

MS/MS spectra acquired at apex of each chromatographic peak for H3.1 L- and D-lactyl $K_{18}QLATK_{23}AAR$ in three injections

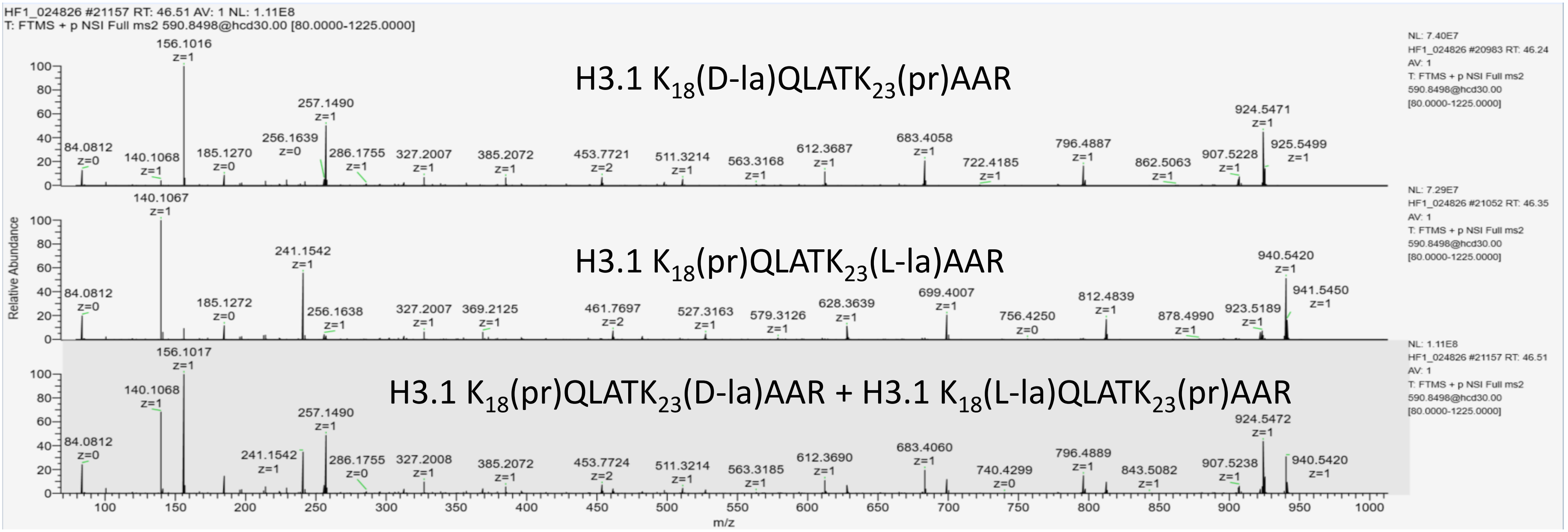

L-la:D-la
1:1

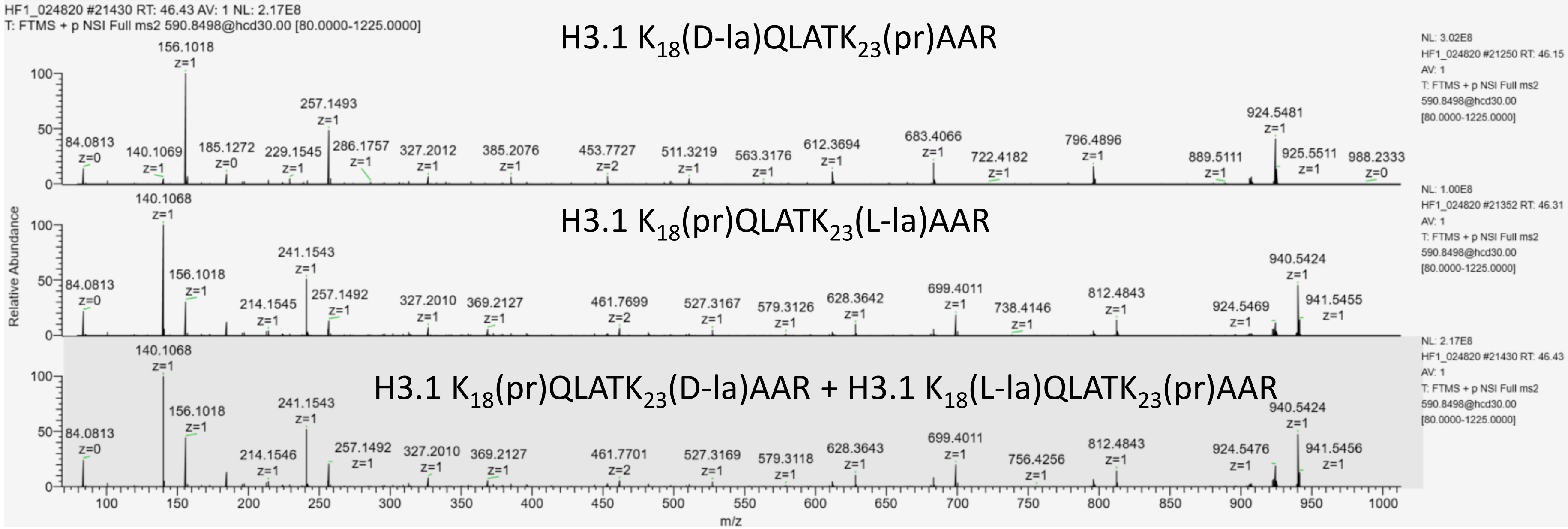

L-la:D-la
1:2

MS/MS spectra acquired at apex of each chromatographic peak for H3.1 L- and D-lactyl $K_{18}QLATK_{23}AAR$

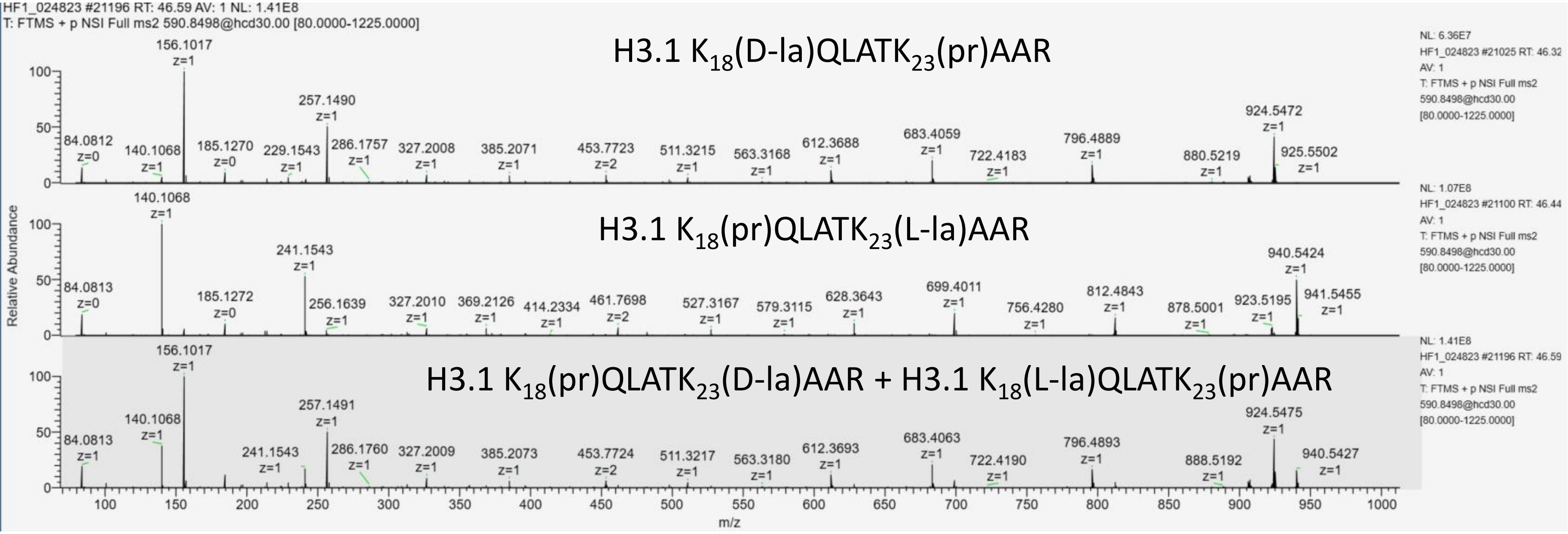

L-la:D-la
2:1

Extracted ion chromatogram of H3.t L- and D-lactyl $K_{18}QLATK_{23}VAR$ (m/z=604.8655,2+)

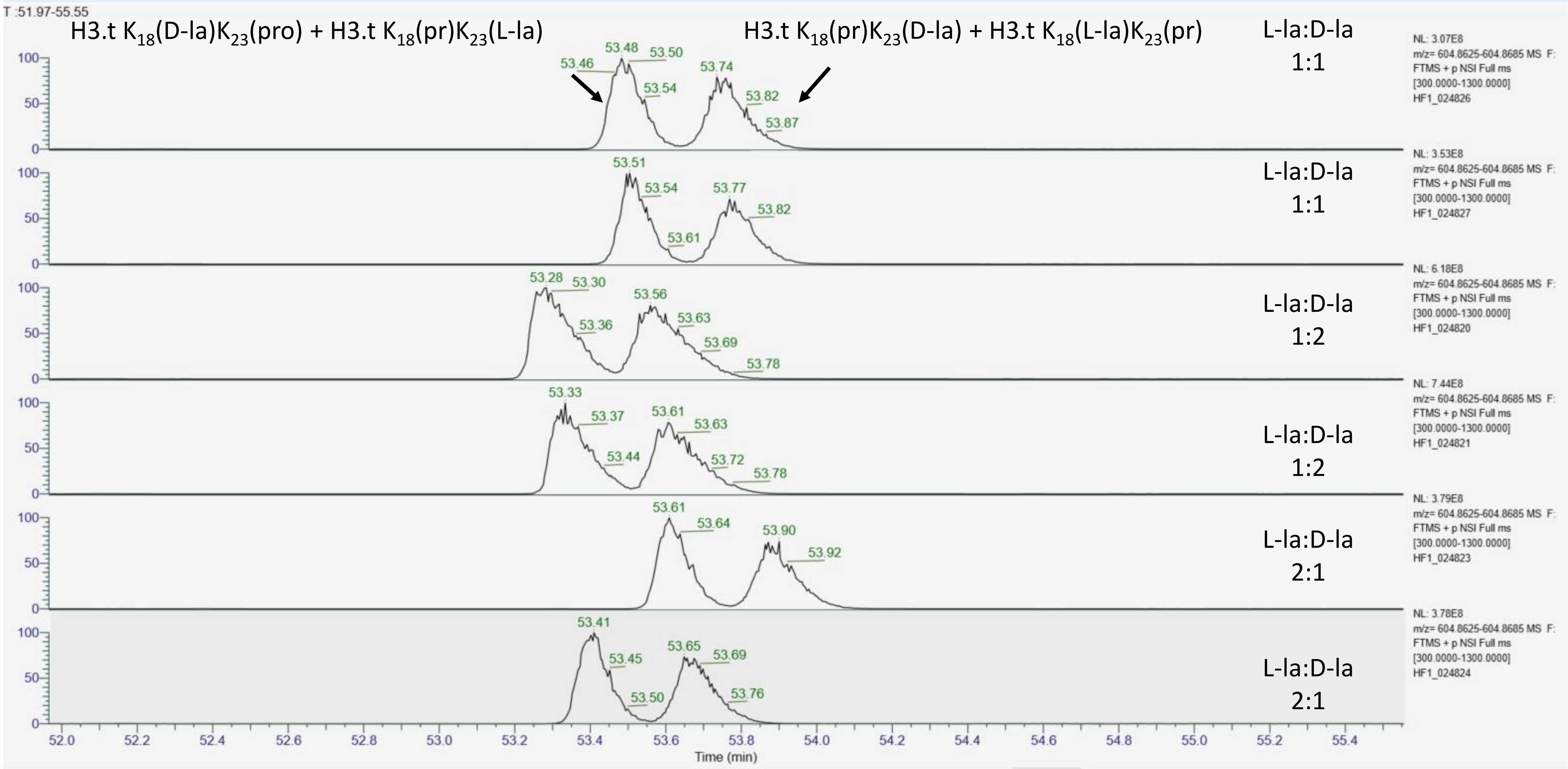

MS/MS spectra acquired at apex of each chromatographic peak for H3.t L- and D-lactyl $K_{18}QLATK_{23}VAR$ in three injections

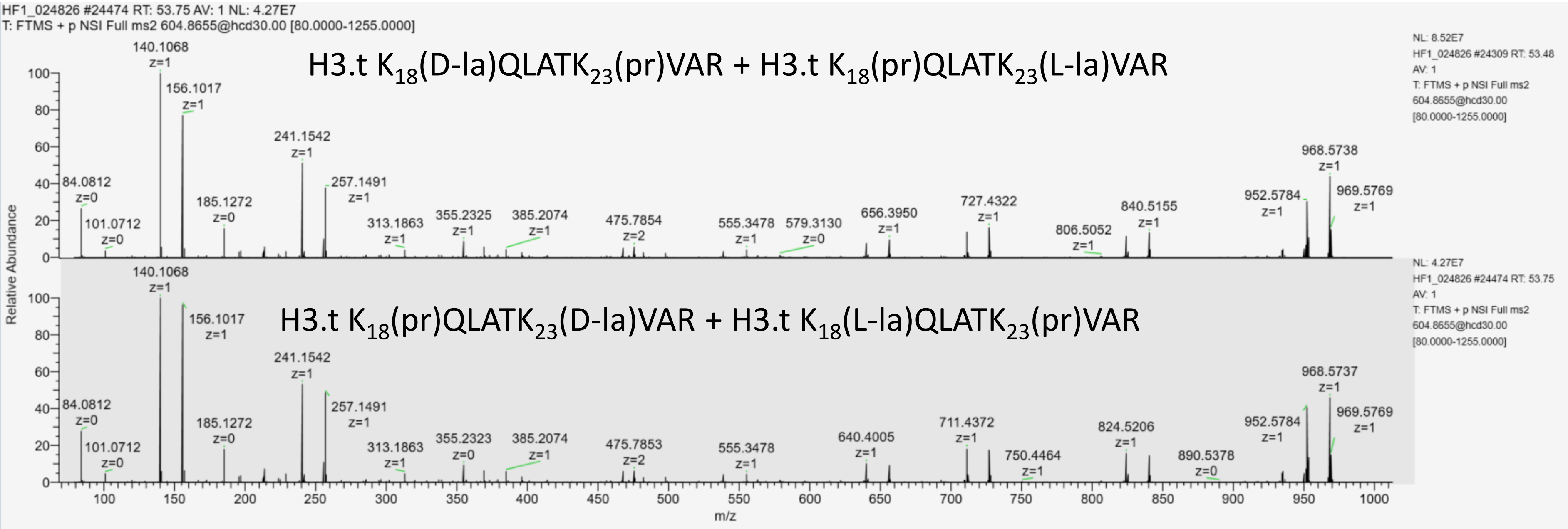

L-la:D-la
1:1

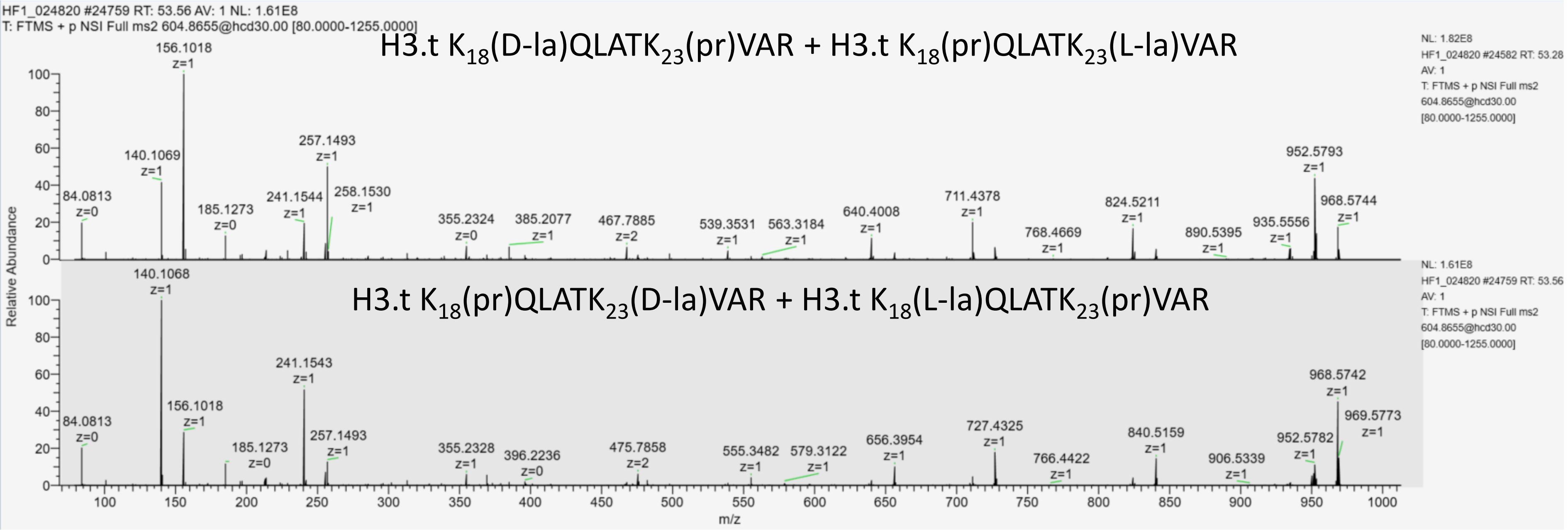

L-la:D-la
1:2

MS/MS spectra acquired at apex of each chromatographic peak for H3.t L- and D-lactyl $K_{18}QLATK_{23}VAR$

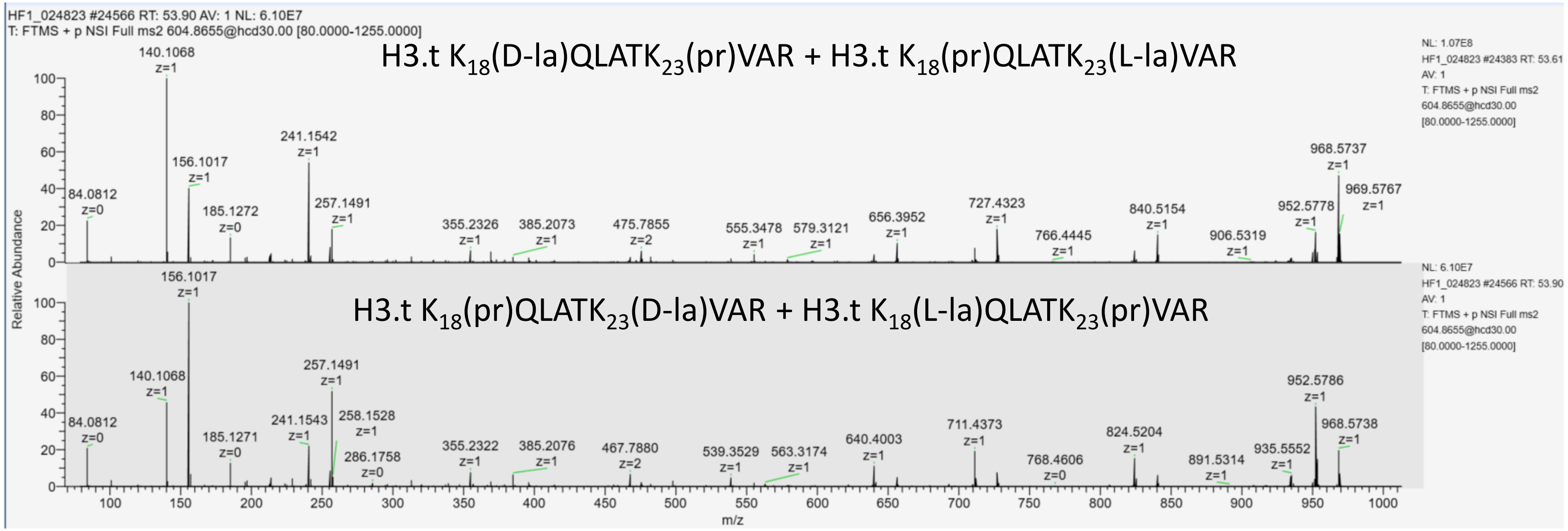

L-la:D-la
2:1

Extracted ion chromatogram of H3.1 K_{27}(la)SAPATGGVK_{36}(pr)K_{37}(pro)PHR (m/z=842.4744,2+)

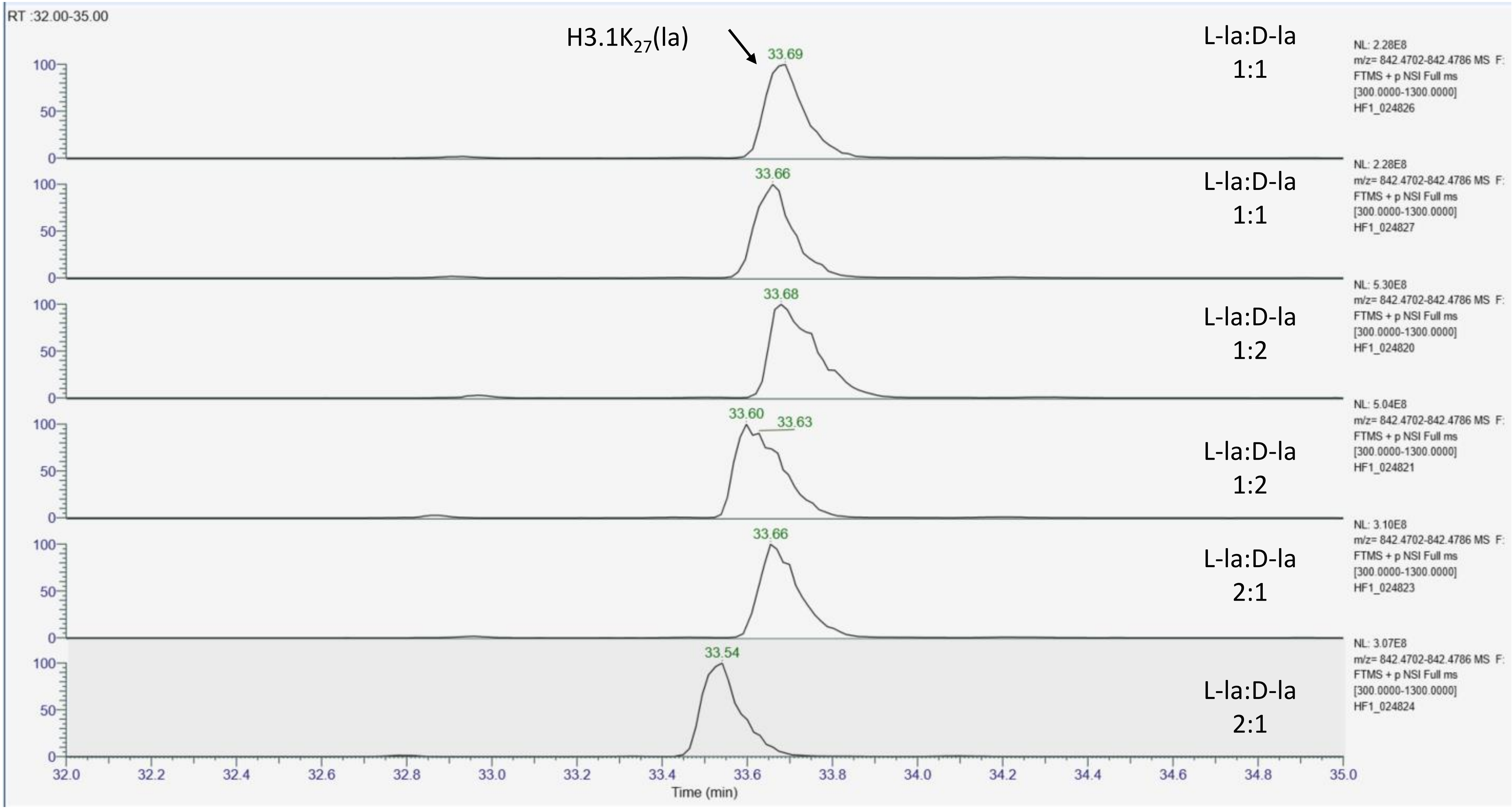

MS/MS spectra acquired at the apex of each chromatographic peak for H3.1 K_{27}(la)SAPATGGVK$_{36}$(pr)K_{37}(pr)PHR in three injections

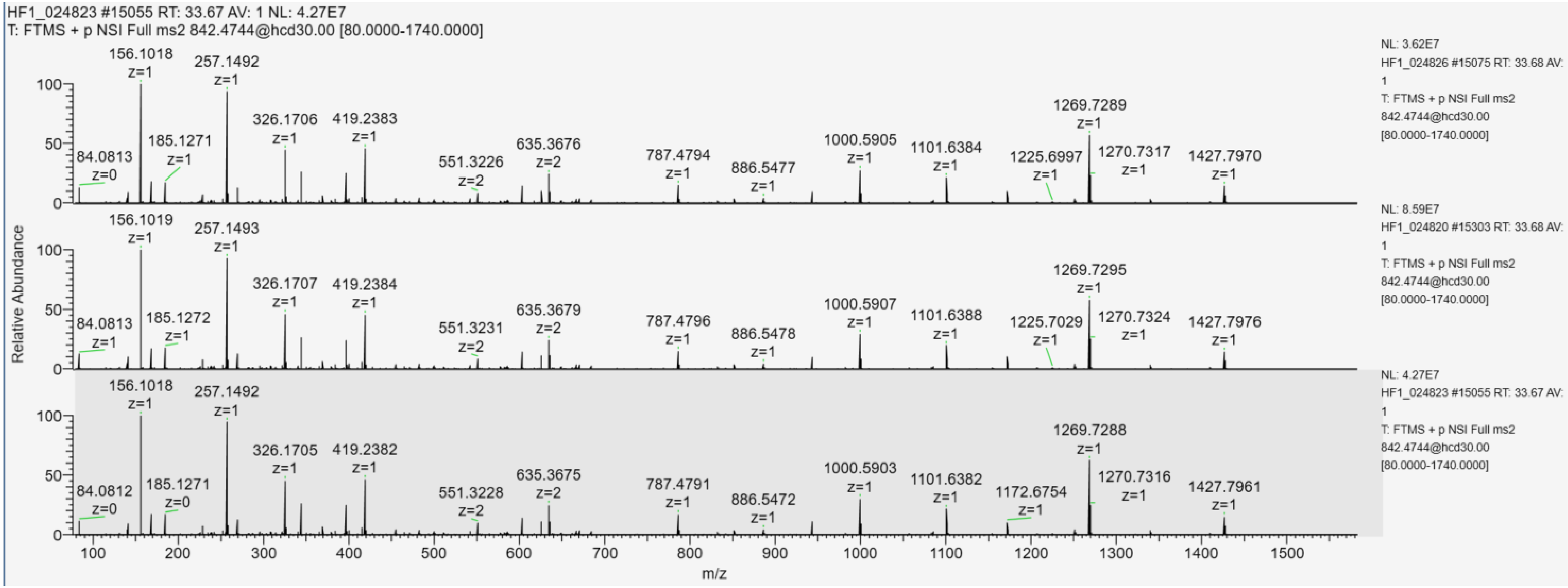

L-la:D-la 1:1

L-la:D-la 1:2

L-la:D-la 2:1

Extracted ion chromatogram of H3.3 K_{27}(la)SAPSTGVK$_{37}$(pr)K_{37}(pr)PHR (m/z=850.4719,2+)

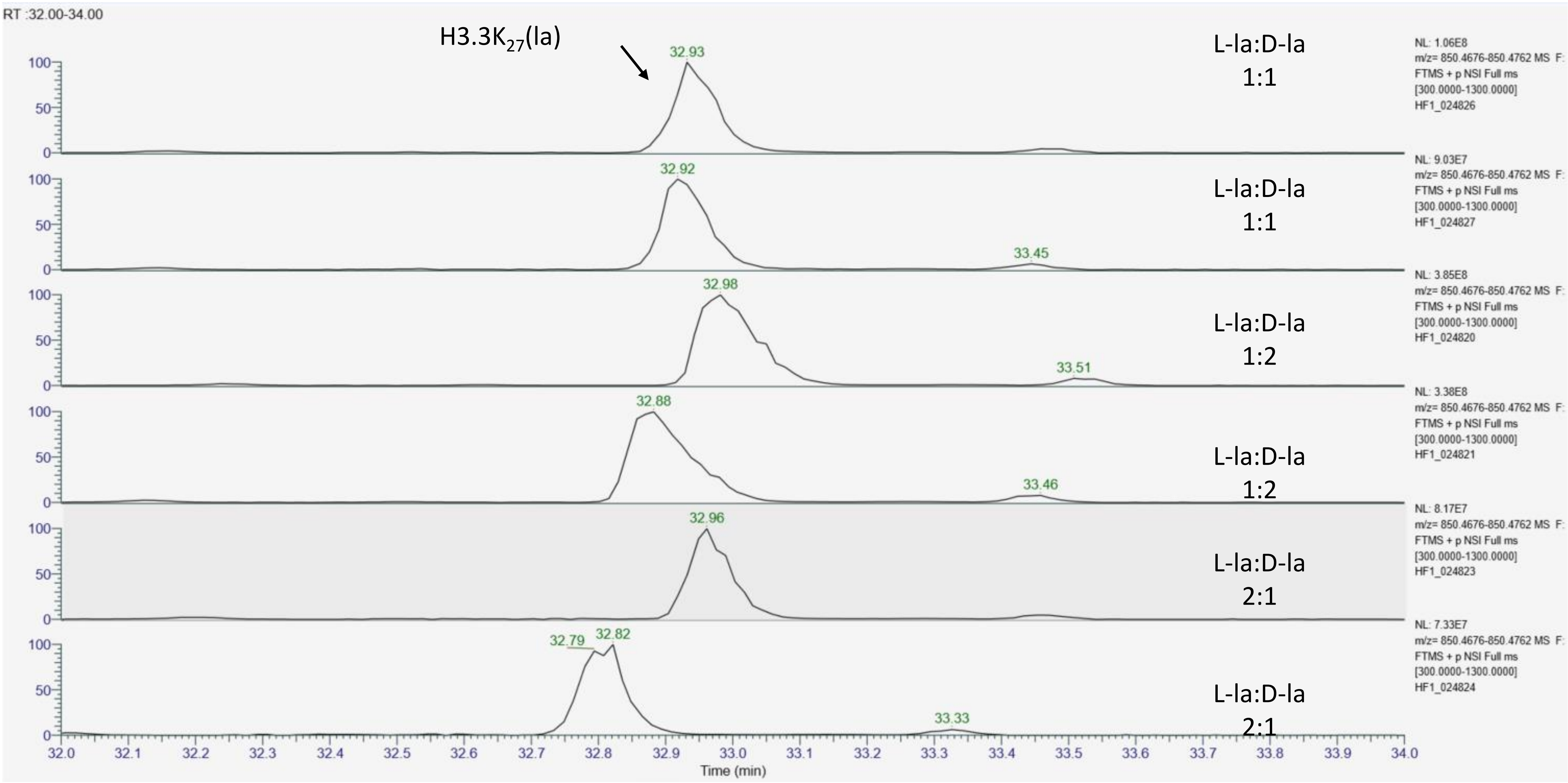

MS/MS spectra acquired at the apex of chromatographic peaks for H3.3 K_{27}(la)SAPSTGVK$_{37}$(pr)K$_{37}$(pr)PHR in three injections

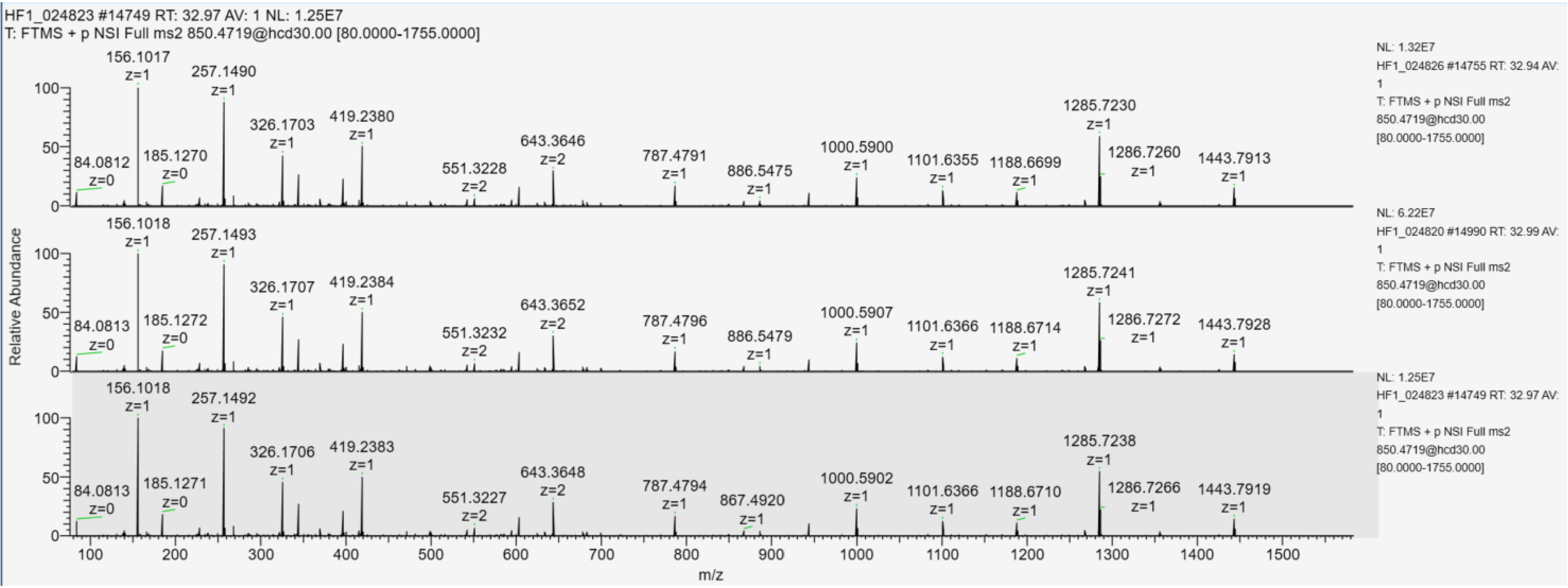

L-la:D-la 1:1

L-la:D-la 1:2

L-la:D-la 2:1

Extracted ion chromatogram of L- and D-lactylated H3 YQK$_{56}$STELLIR (m/z=694.8866,2+)

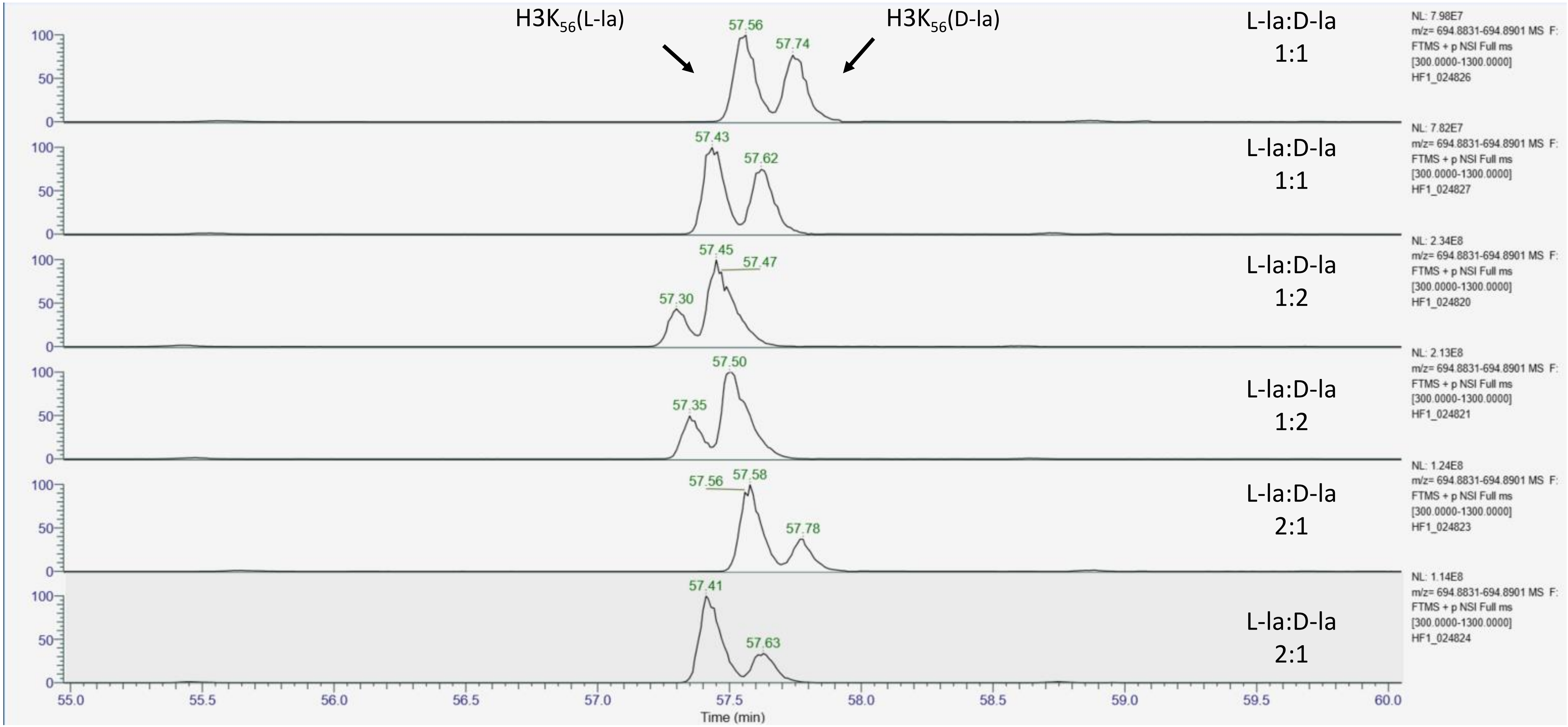

MS/MS spectra acquired at apex of each chromatographic peak for L- and D-lactylated H3 YQK$_{56}$STELLIR

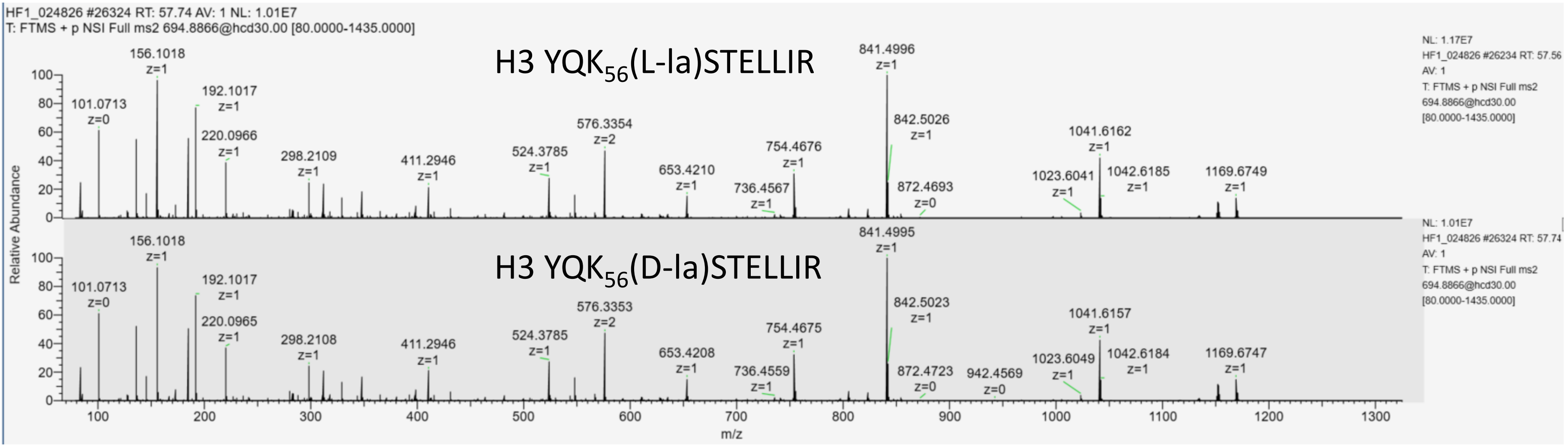

L-la:D-la
1:1

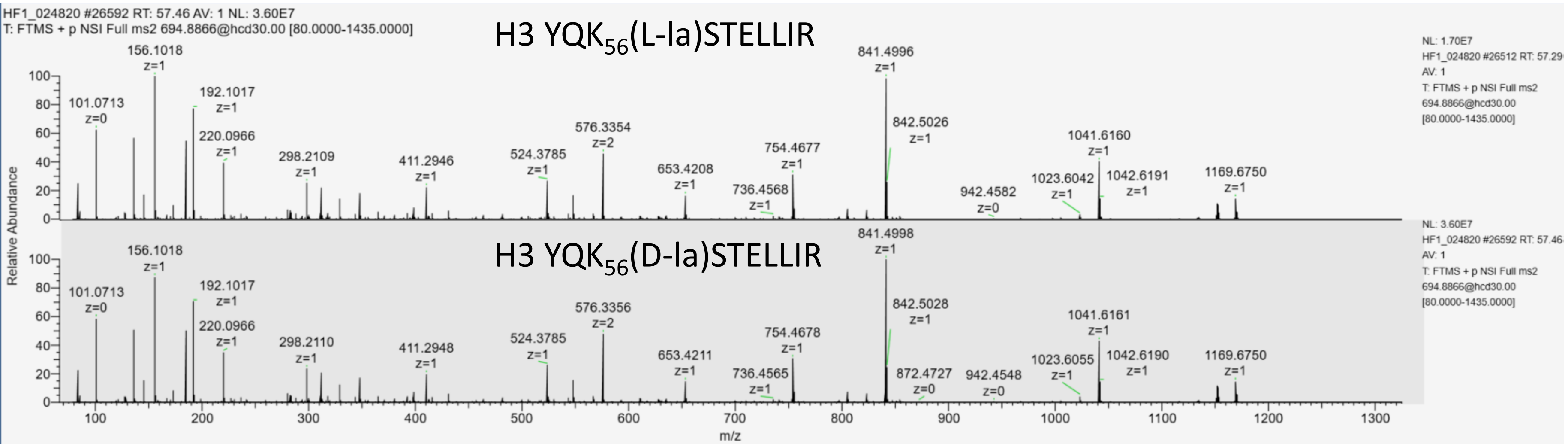

L-la:D-la
1:2

MS/MS spectra acquired at apex of each chromatographic peak for L- and D-lactylated H3 YQK$_{56}$STELLIR

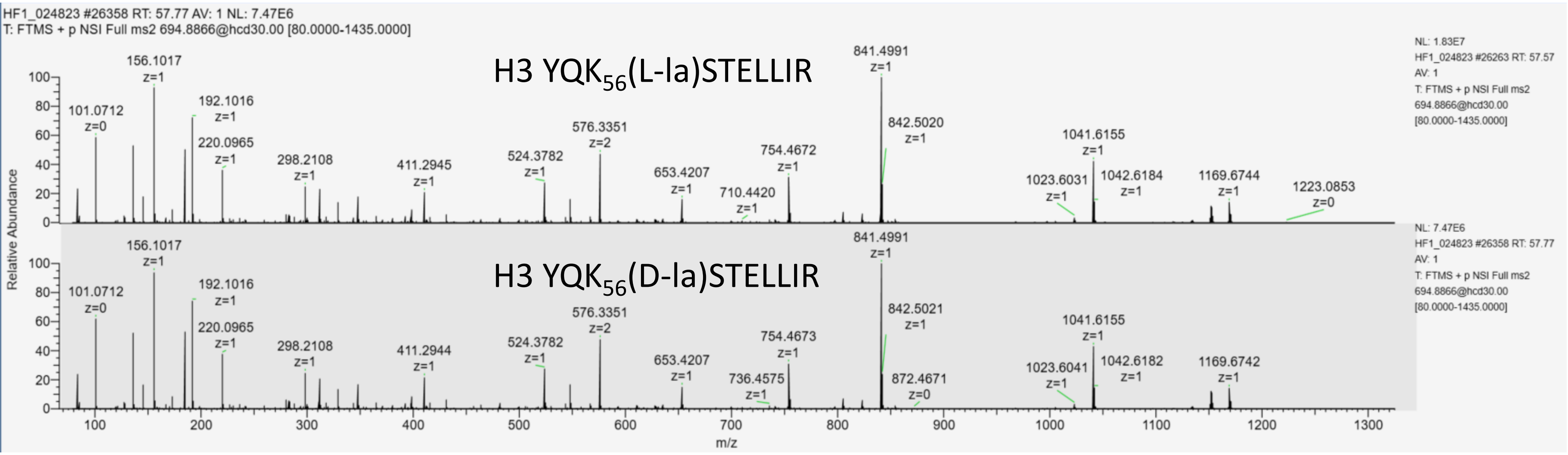

L-la:D-la
2:1

Extracted ion chromatogram of H3 K_{64}(la)LPFQR (m/z=463.7703,2+)

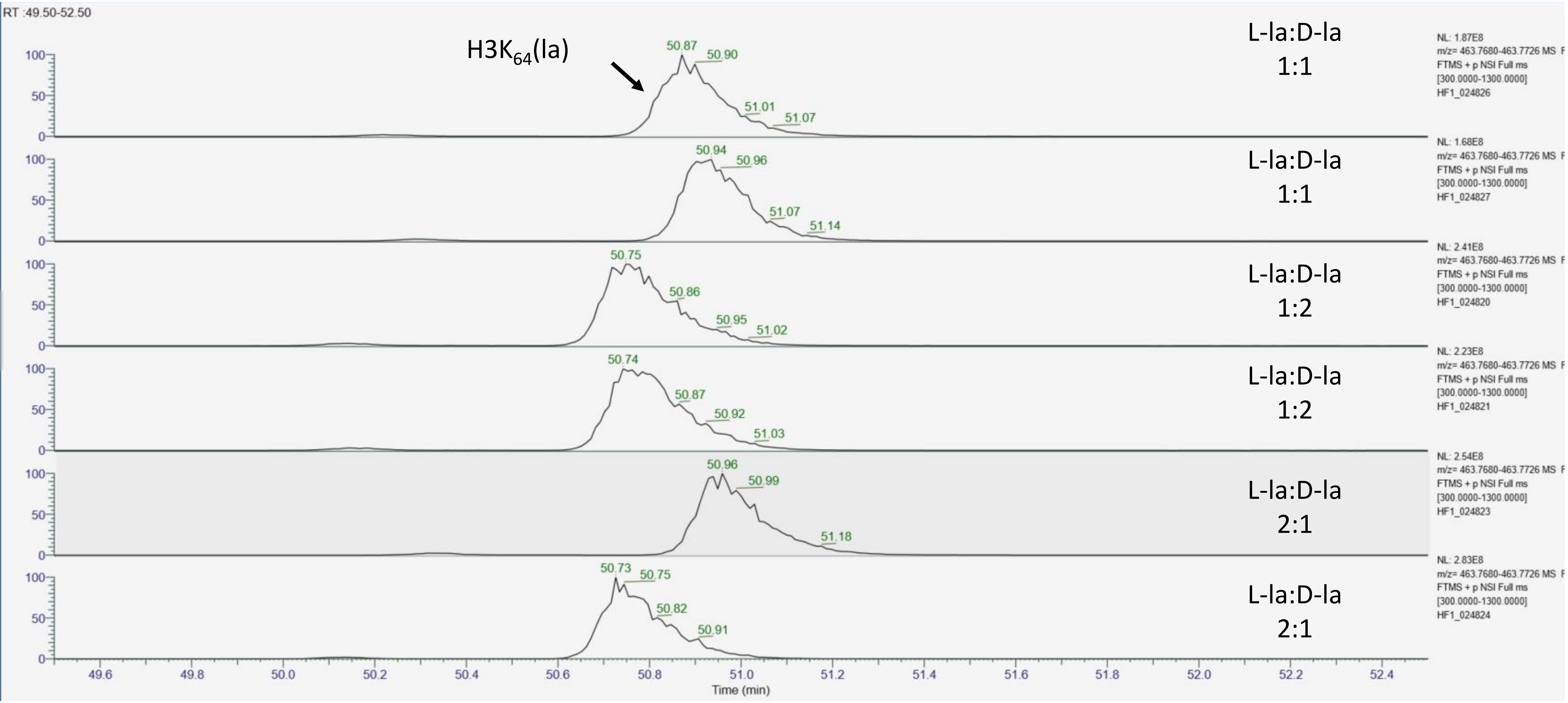

MS/MS spectra acquired at apex of each chromatographic peak for H3 K_{64}(la)LPFQR

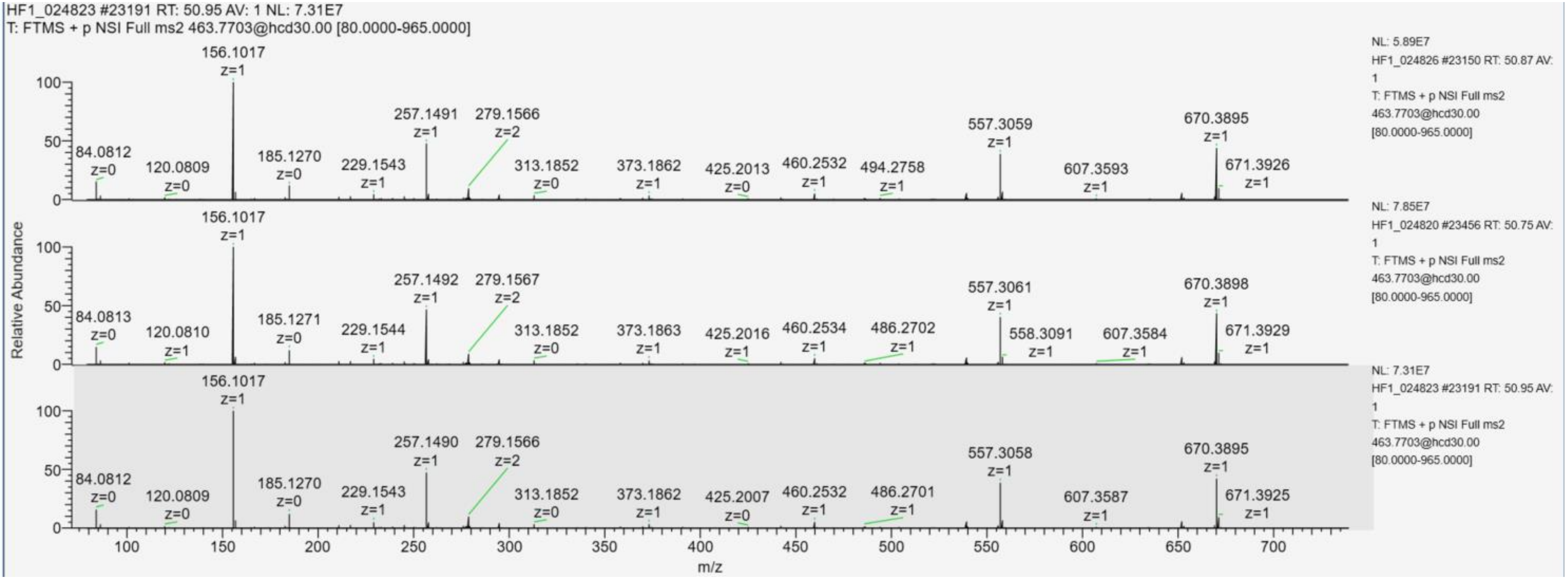

L-la:D-la 1:1

L-la:D-la 1:2

L-la:D-la 2:1

Extracted ion chromatogram of L- and D-lactylated H3 EIAQDFK$_{79}$TDLR (m/z=737.3766,2+)

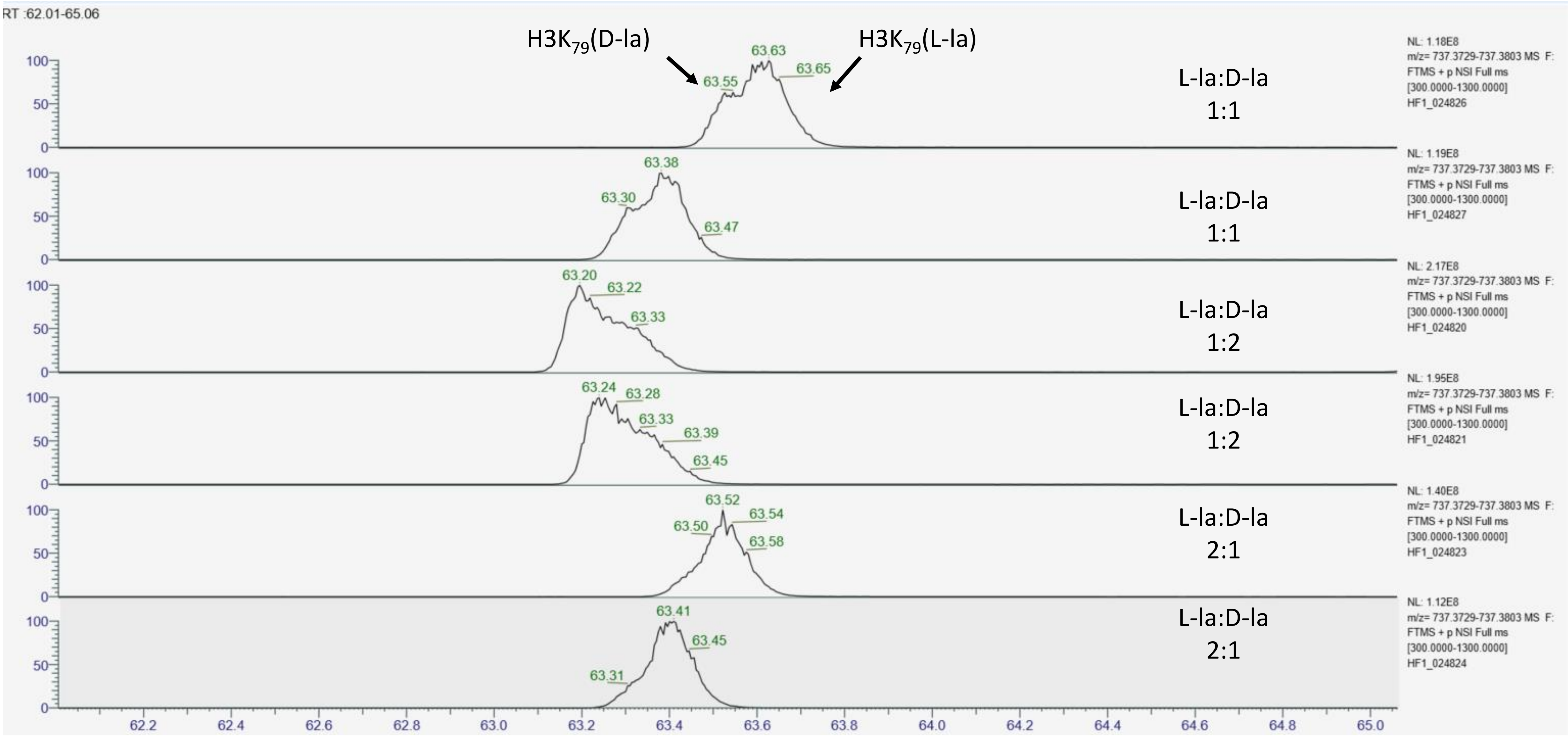

MS/MS spectra acquired at apex of each chromatographic peak for L- and D-lactylated H3 EIAQDFK$_{79}$TDLR

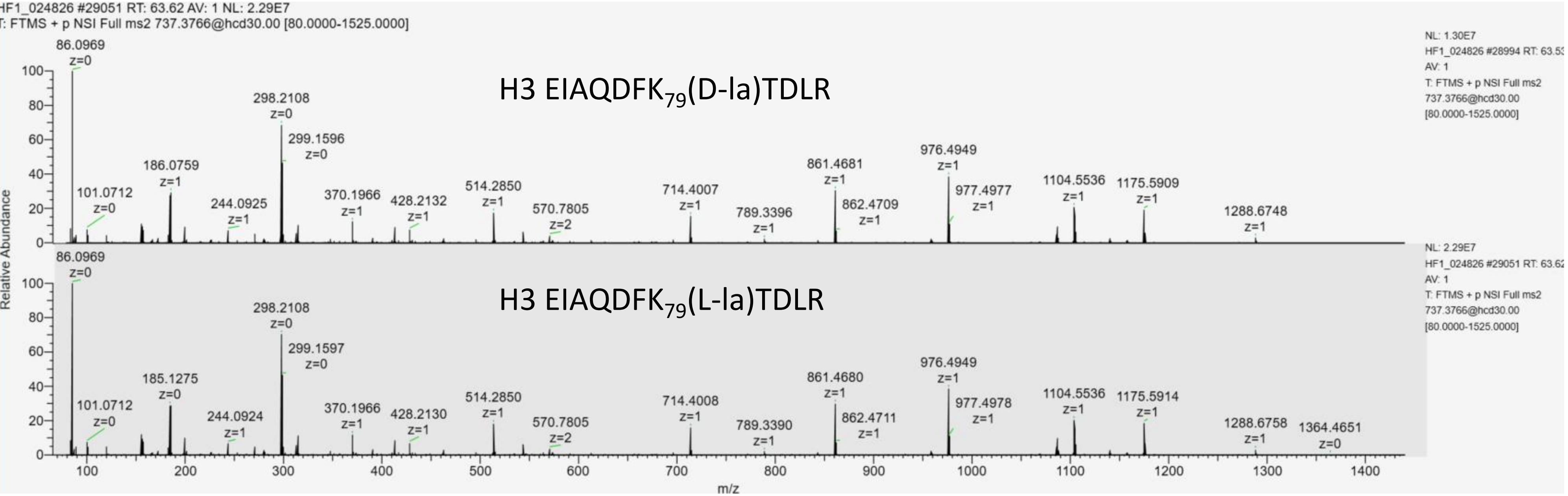

L-la:D-la
1:1

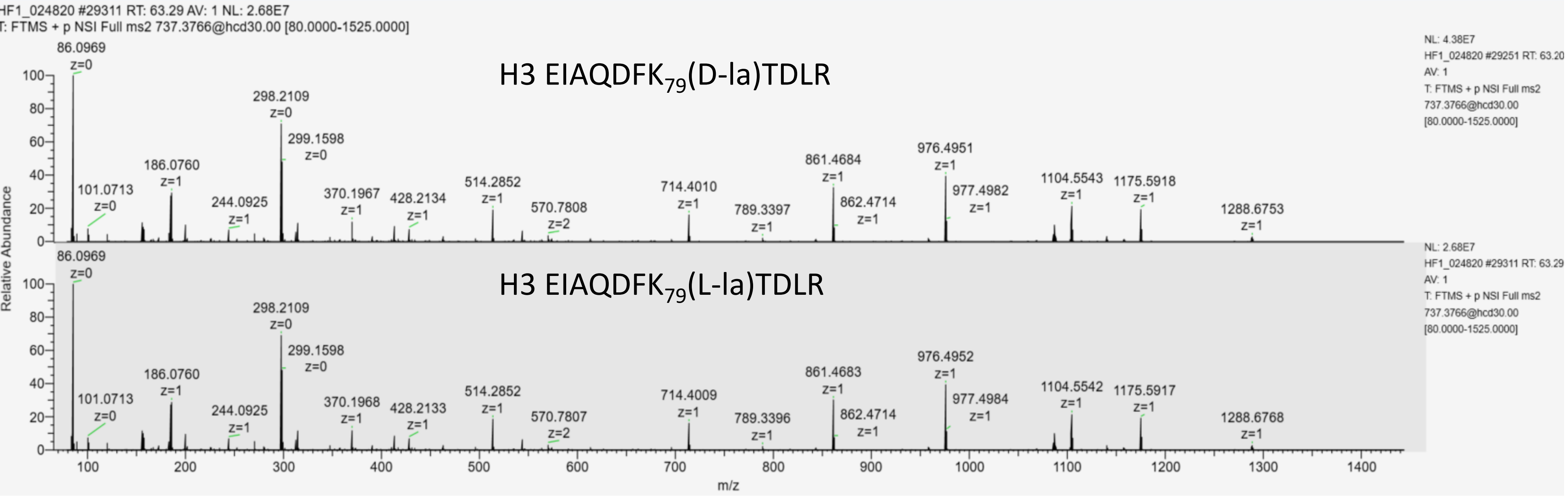

L-la:D-la
1:2

MS/MS spectra acquired at apex of each chromatographic peak for L- and D-lactylated H3 EIAQDFK$_{79}$TDLR

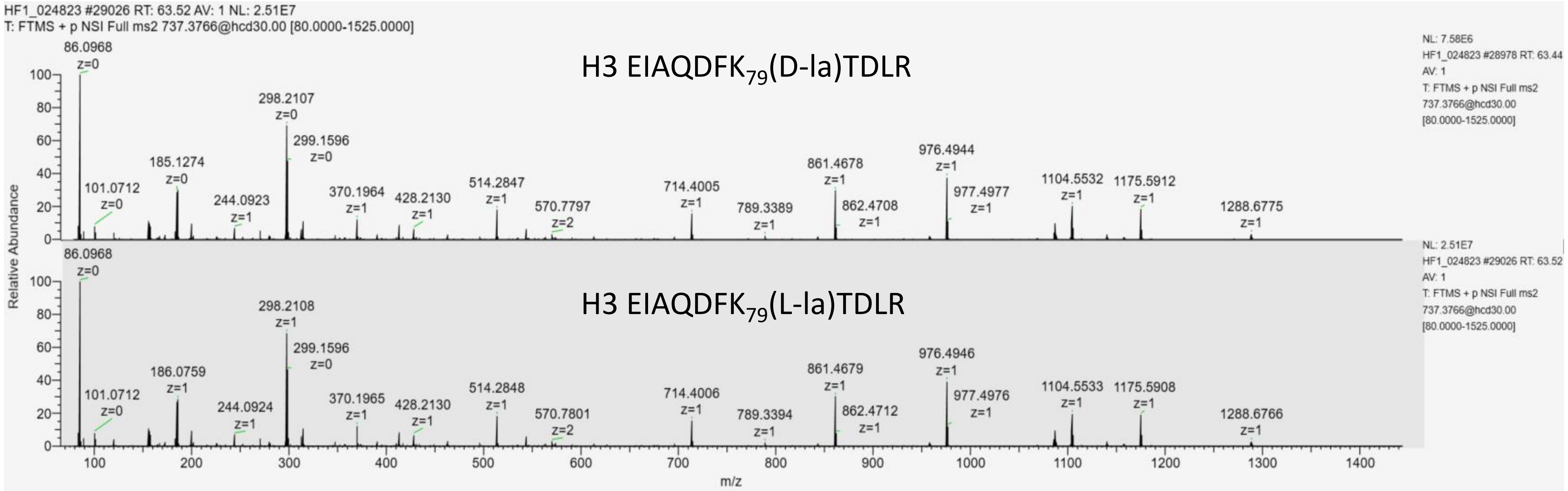

L-la:D-la
1:2

Extracted ion chromatogram of H3 VTIMPK$_{122}$(la)DIQLAR (761.9305,2+)

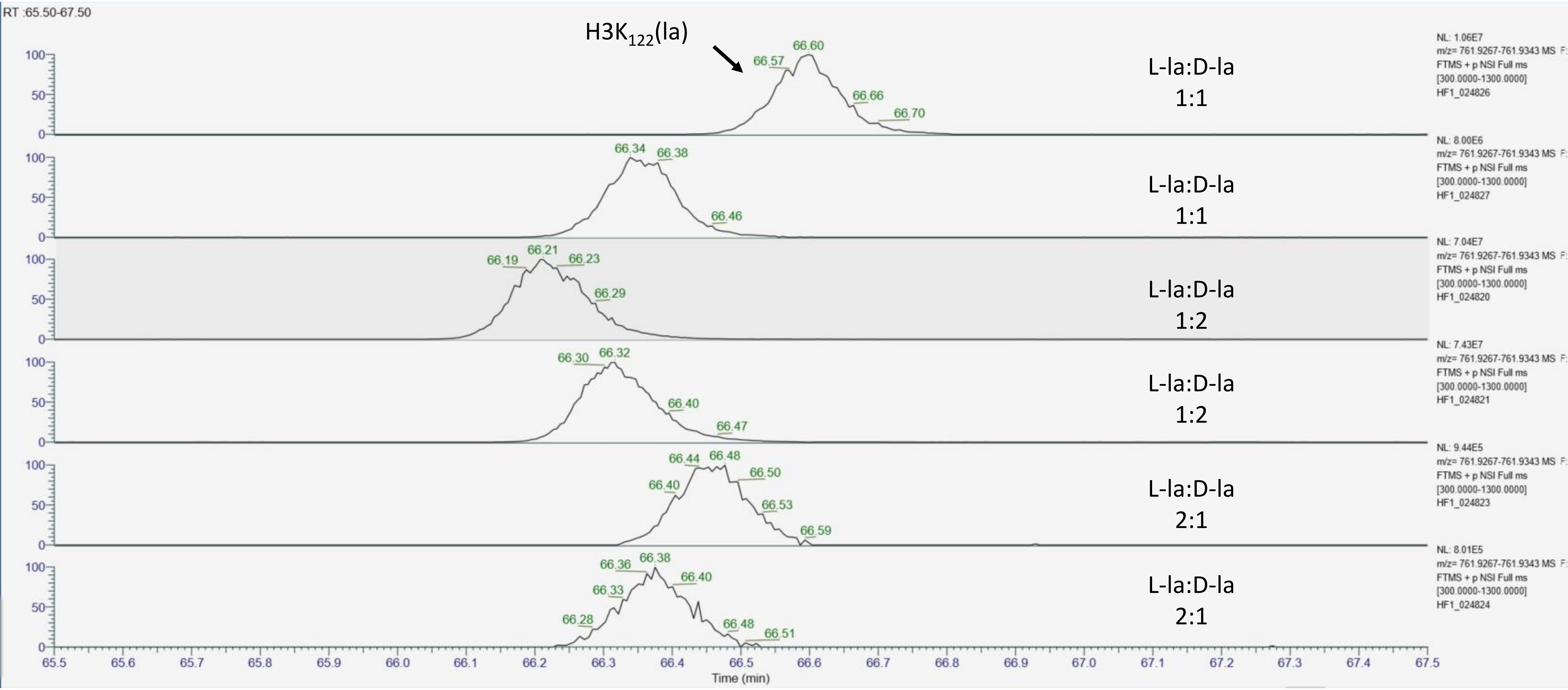

MS/MS spectra acquired at apex of each chromatographic peak for H3 VTIMPK$_{122}$(la)DIQLAR in three injections

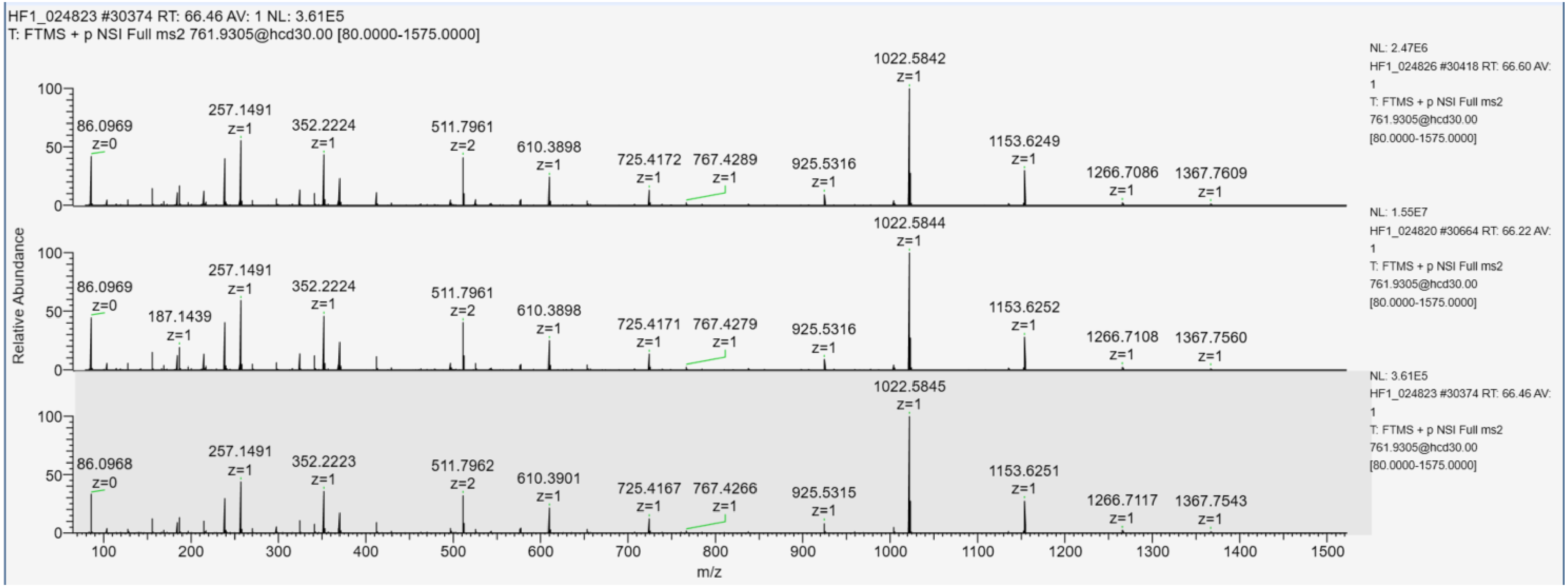

L-la:D-la 1:1

L-la:D-la 1:2

L-la:D-la 2:1

Extracted ion chromatogram of H3 VTIM(ox)PK$_{122}$(la)DIQLAR (m/z=769.9279,2+)

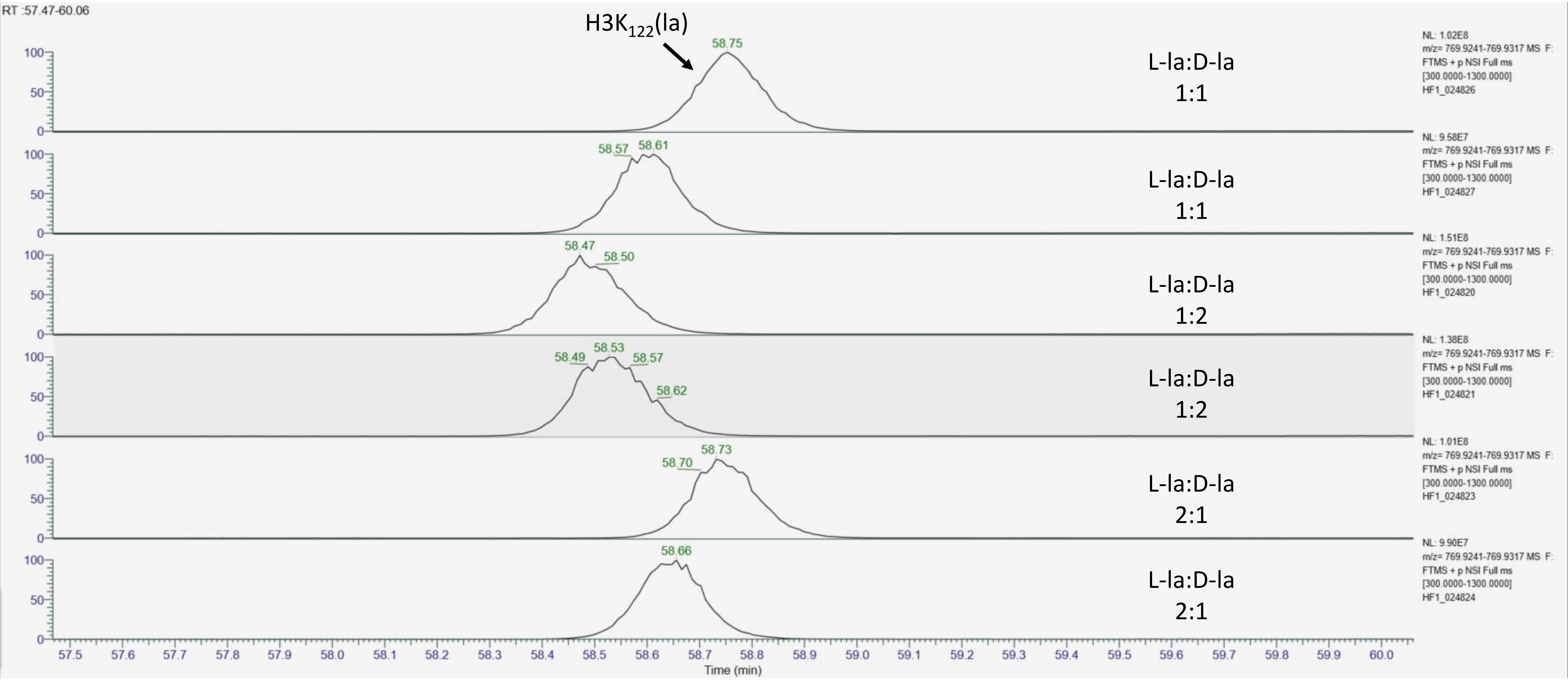

MS/MS spectra acquired at apex of each chromatographic peak for lactylated H3 VTIM(ox)P_{122}DIQLAR in three injections

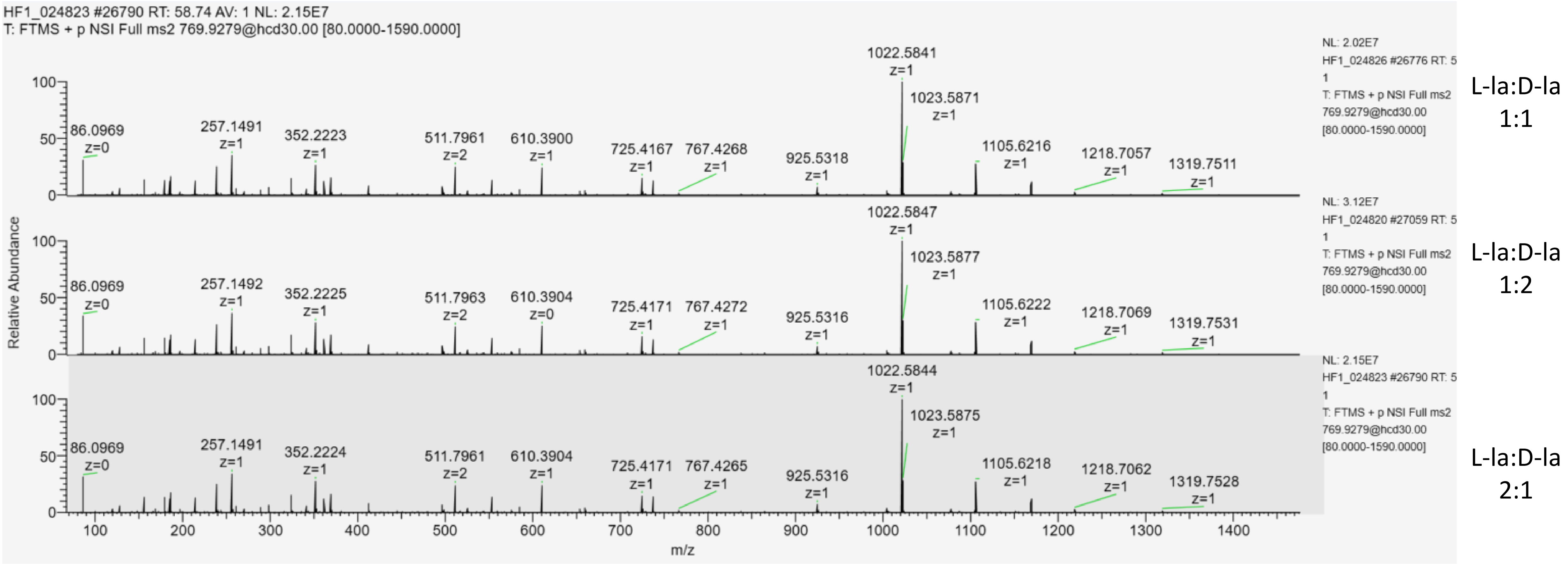

Extracted ion chromatogram of lactylated H4 $GK_5GGK_8GLGK_{12}GGAK_{16}R$ (m/z= 788.9559,2+)

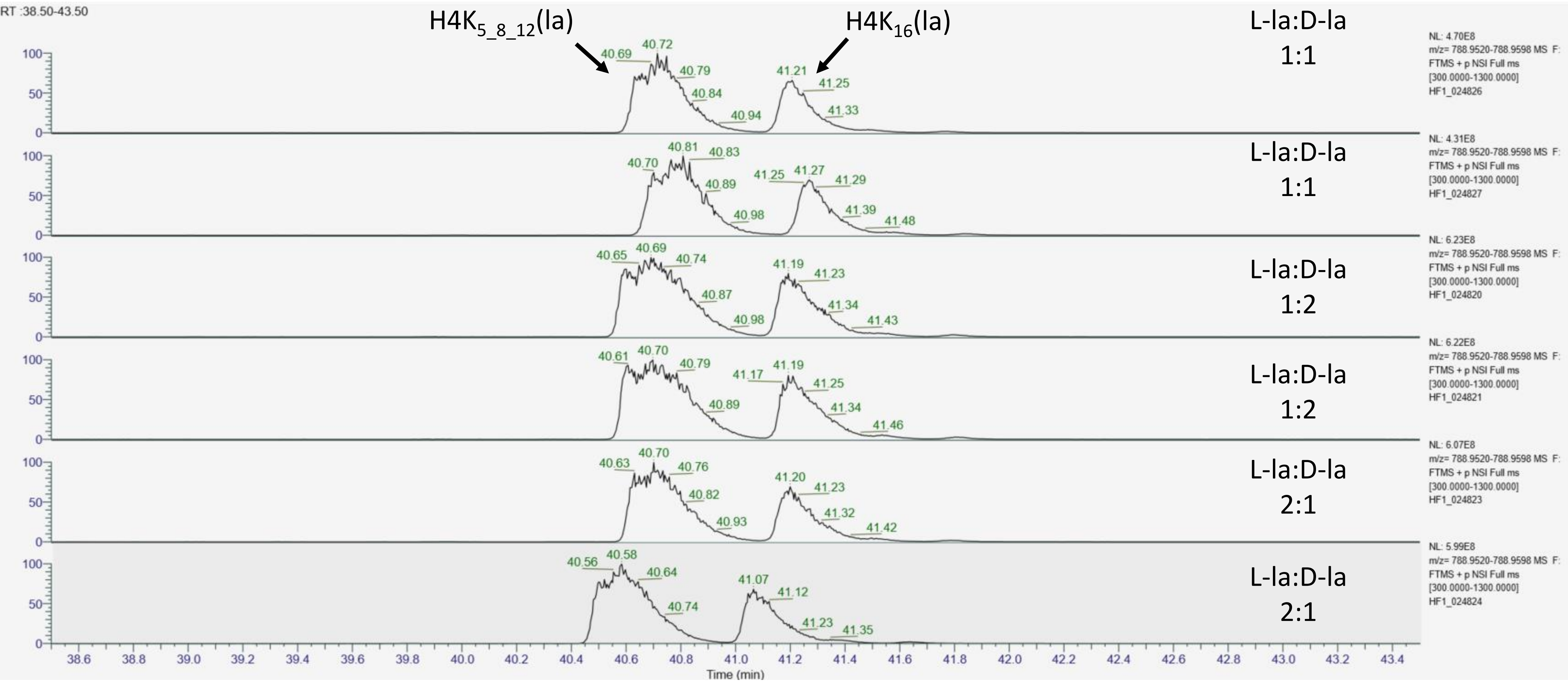

MS/MS spectra acquired at the apex of each chromatographic peak for H4 GK$_5$(pr)GGK$_8$(pr)GLGK$_{12}$(la)GGAK$_{16}$(pr)R in three injections

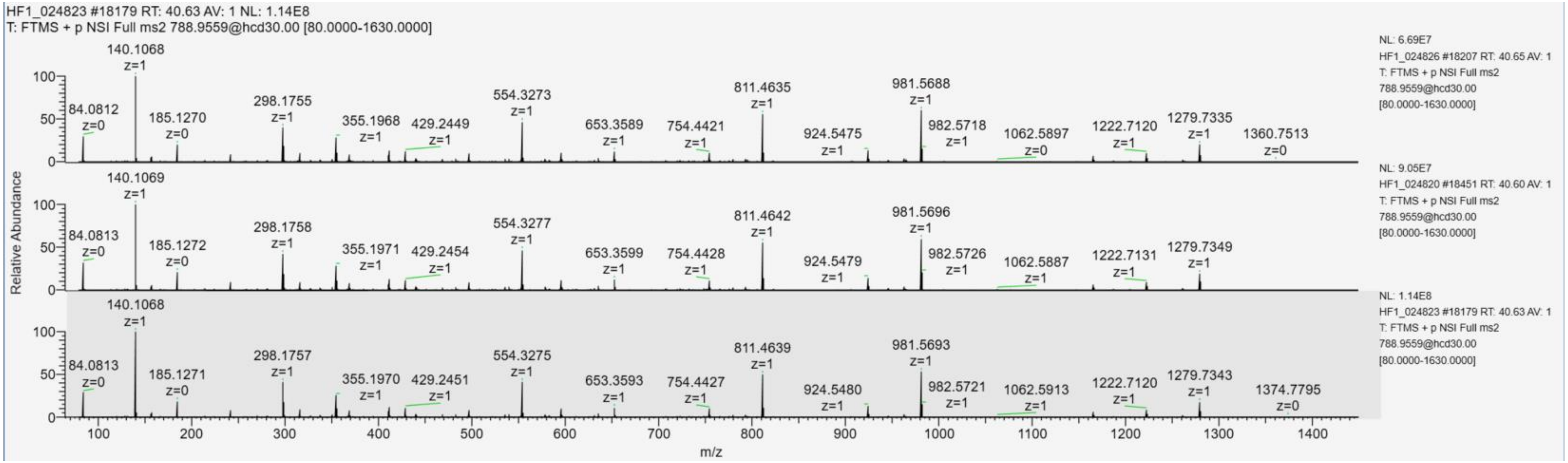

L-la:D-la
1:1

L-la:D-la
1:2

L-la:D-la
2:1

MS/MS spectra acquired at the apex of each chromatographic peak for H4 $GK_5(la)GGK_8(pr)GLGK_{12}(pr)GGAK_{16}(pr)R$ and $GK_5(pr)GGK_8(la)GLGK_{12}(pr)GGAK_{16}(pr)R$

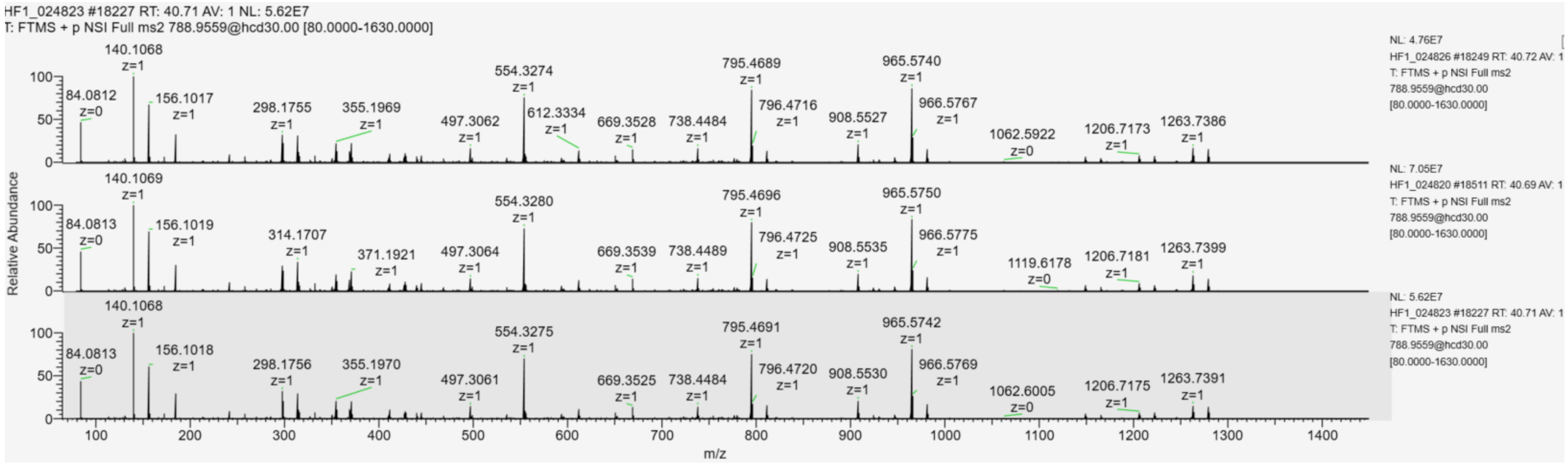

L-la:D-la
1:1

L-la:D-la
1:2

L-la:D-la
2:1

MS/MS spectra acquired at the apex of each chromatographic peak for H4 GK_5(pr)GGK_8(pr)$GLGK_{12}$(pr)$GGAK_{16}$(la)R

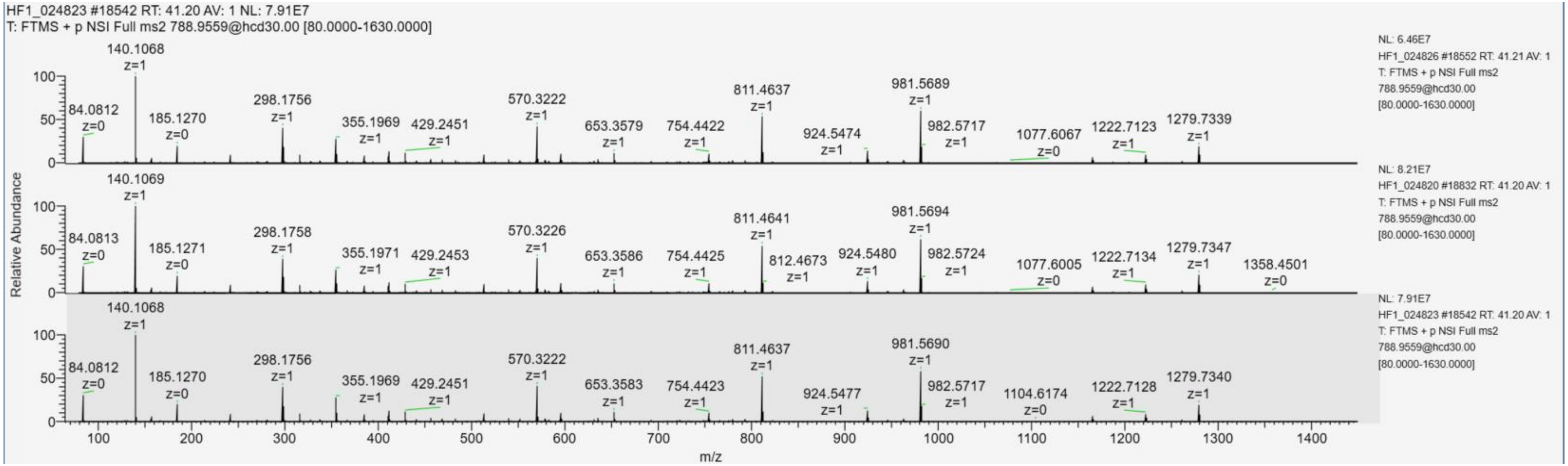

L-la:D-la 1:1

L-la:D-la 1:2

L-la:D-la 2:1

Extracted ion chromatogram of lactylated H4 DNIQGITK$_{31}$PAIR (m/z= 732.4082,2+)

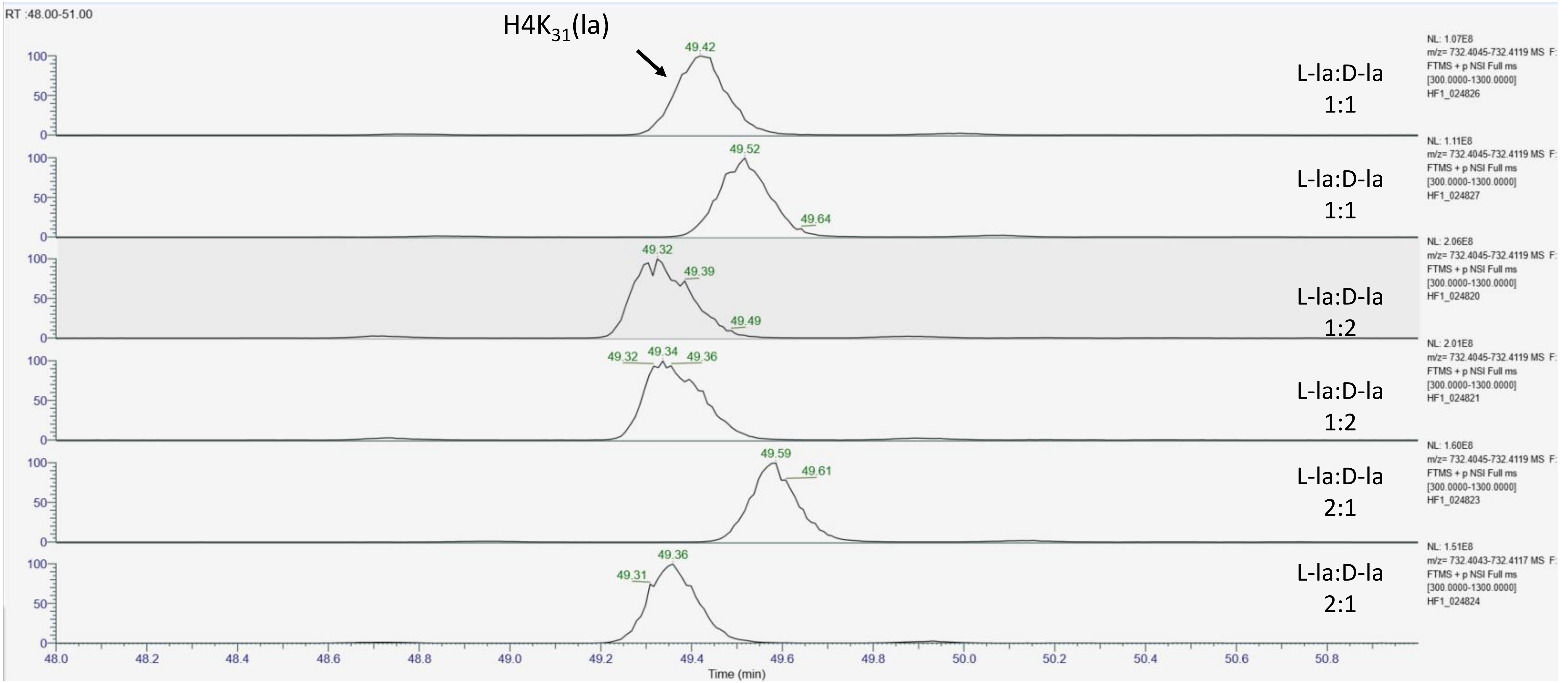

MS/MS spectra acquired at apex of each chromatographic peak for H4 DNIQGITK$_{31}$(la)R in three injections

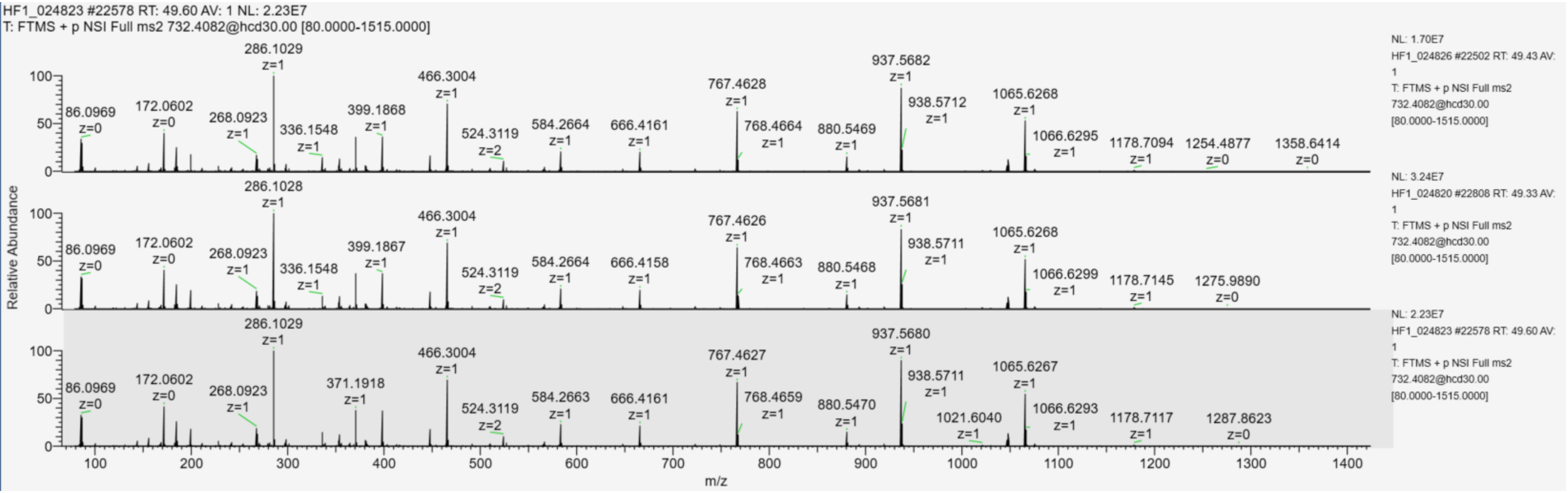

Extracted ion chromatogram of L- and D-lactylated H4 DAVTYTEHAK$_{77}$R (m/z=714.8533,2+)

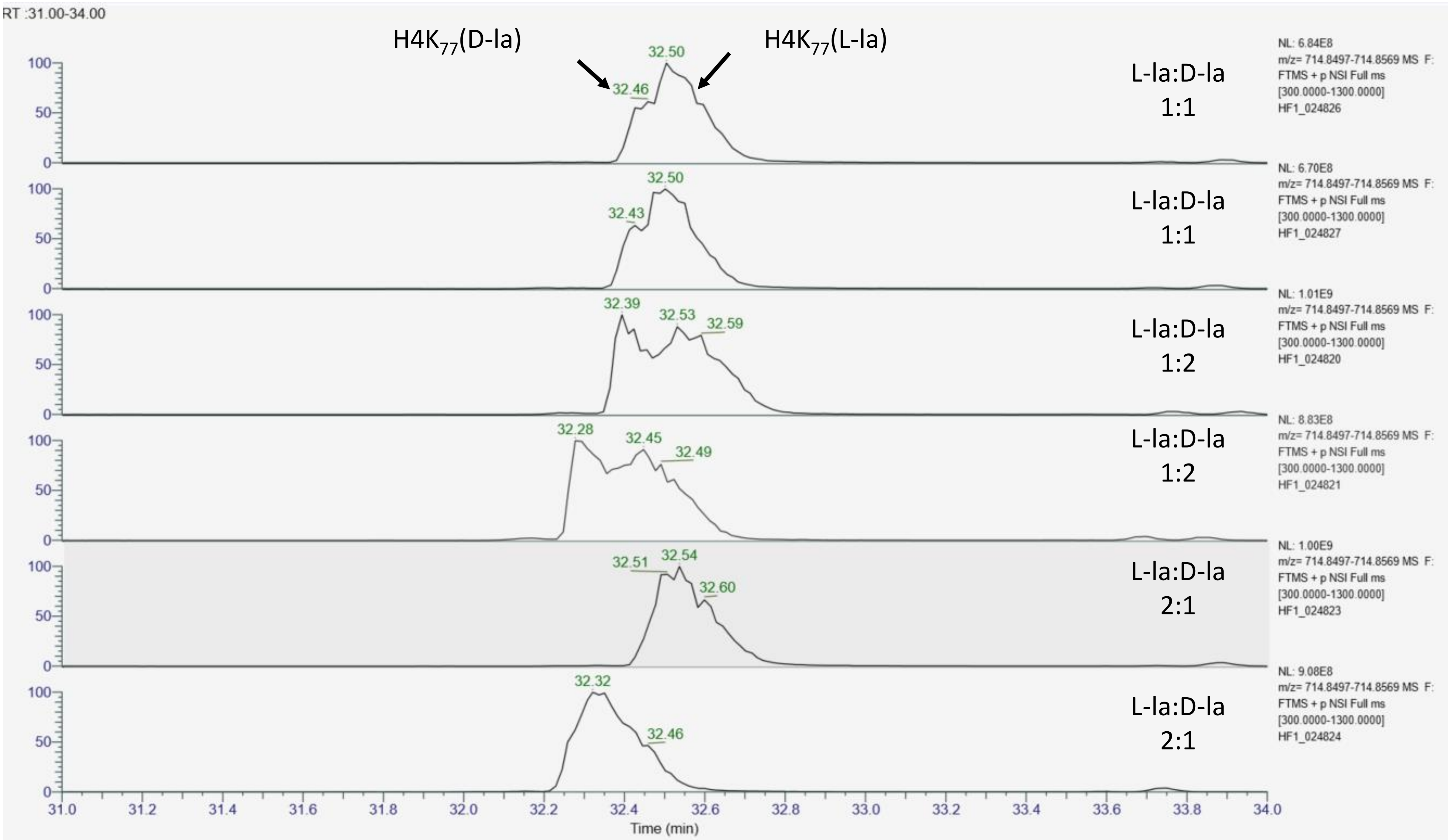

MS/MS spectra acquired at the apex of each chromatographic peak for L- and D-lactylated H4 DAVTYTEHAK_{77}R

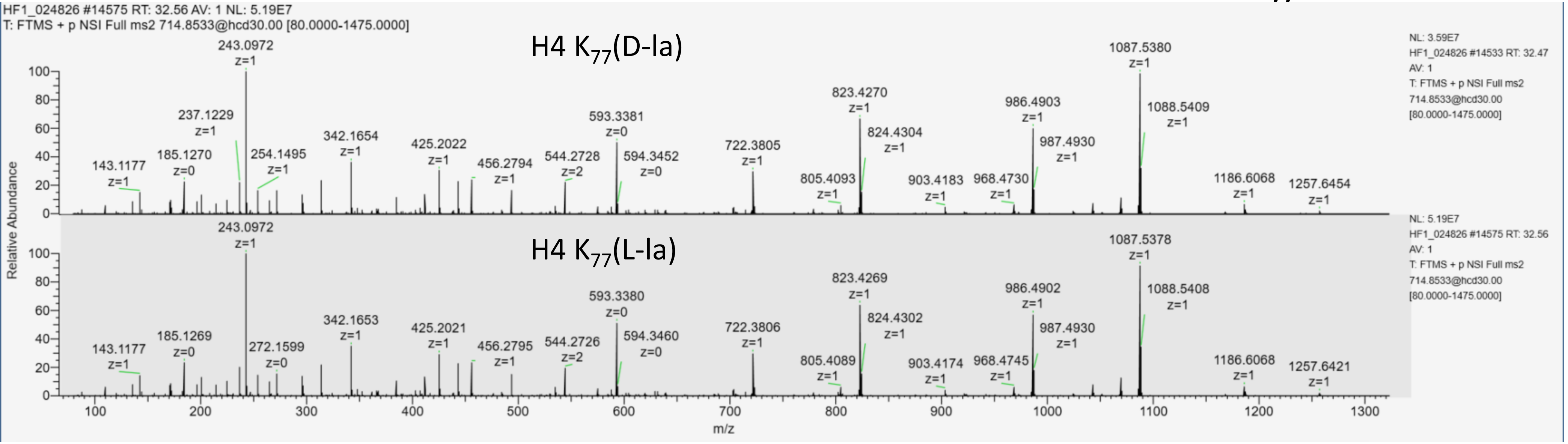

L-la:D-la
1:1

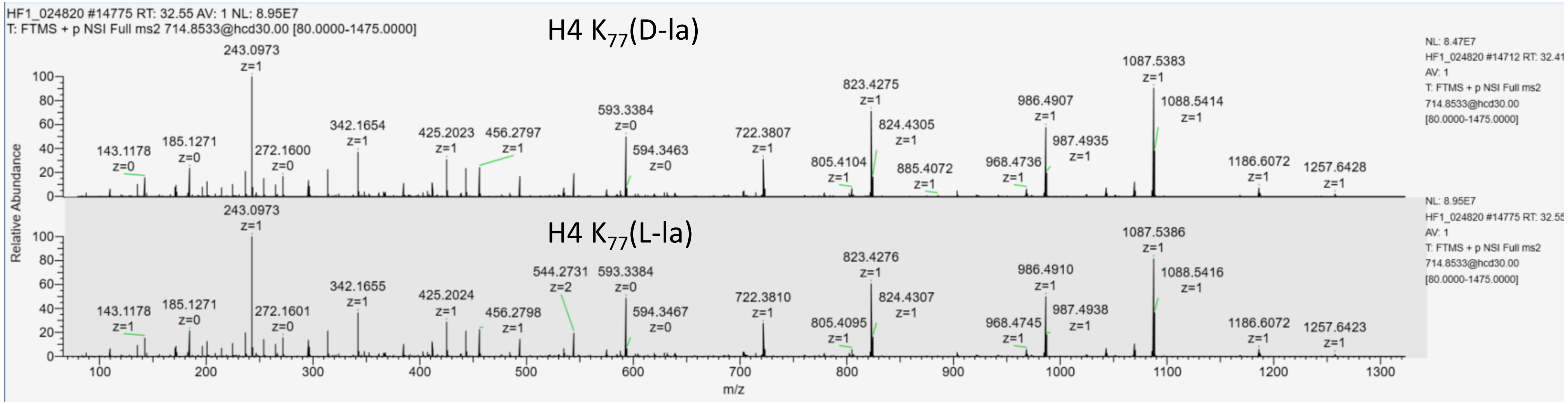

L-la:D-la
1:2

MS/MS spectra acquired at apex of each chromatographic peak for L- and D-lactylated H4 DAVTYTEHAK$_{77}$R

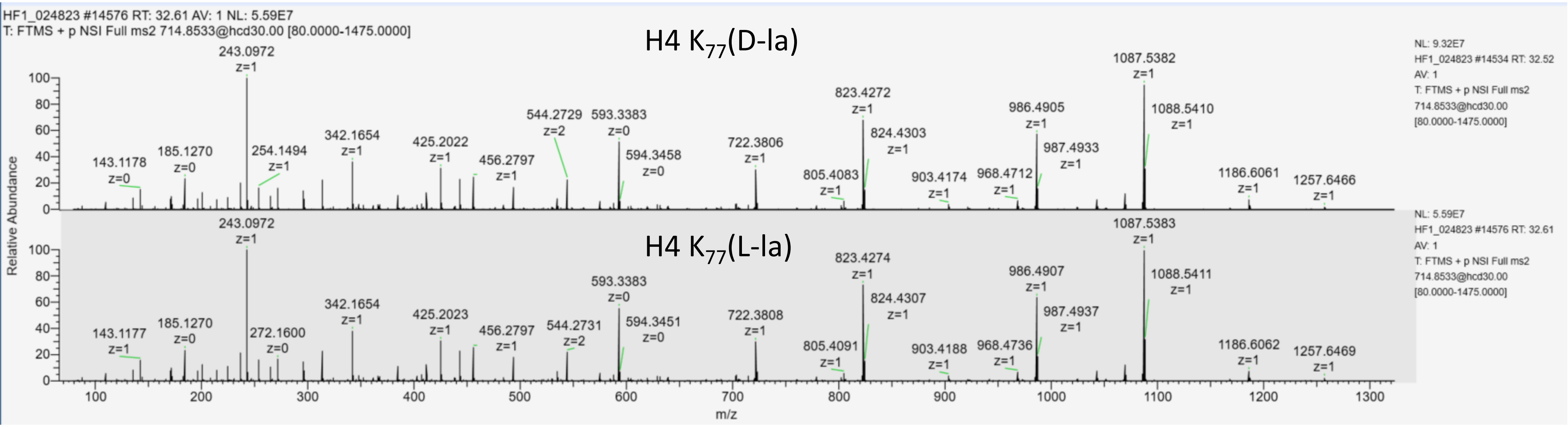

L-la:D-la
2:1

Extracted ion chromatogram of lactylated H4 K_{79}TVTAMDVVYALK_{91}R (m/z= 894.9938 ,2+)

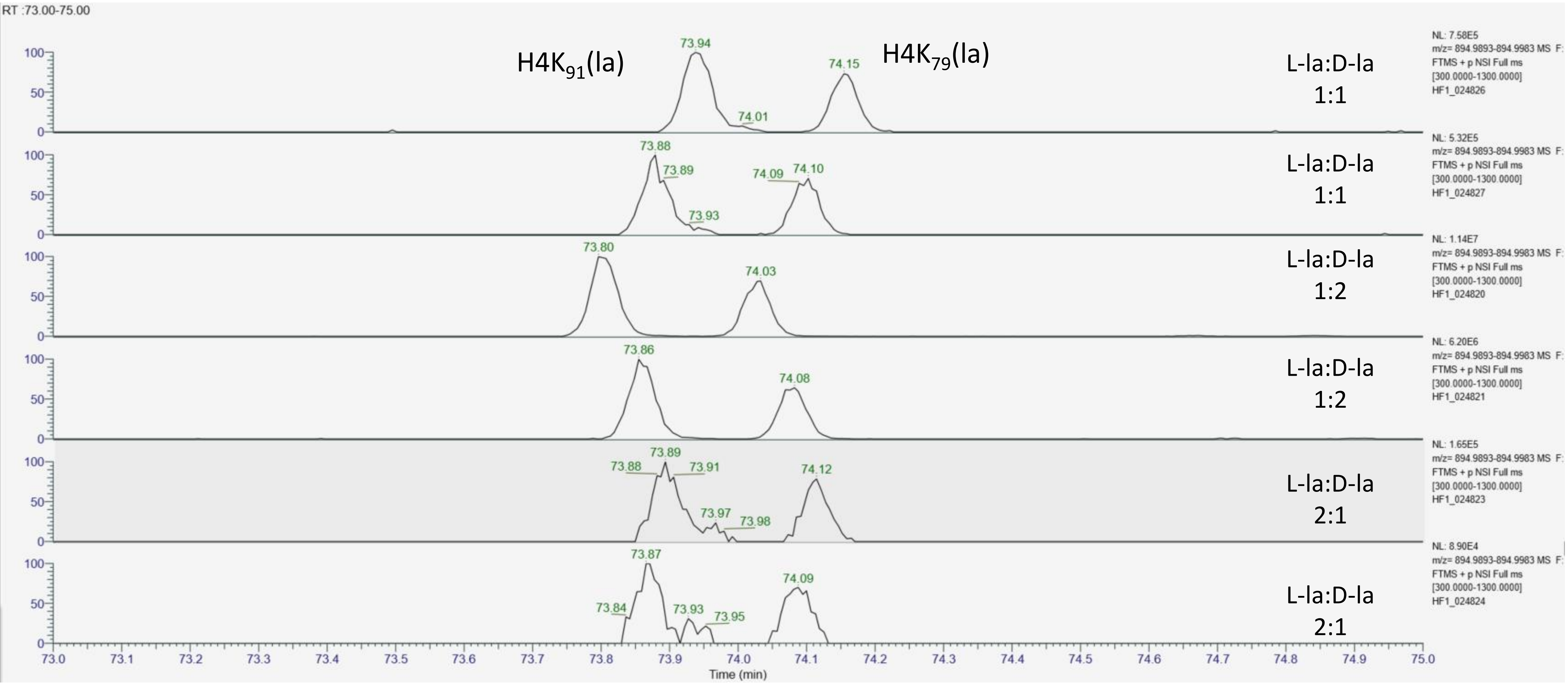

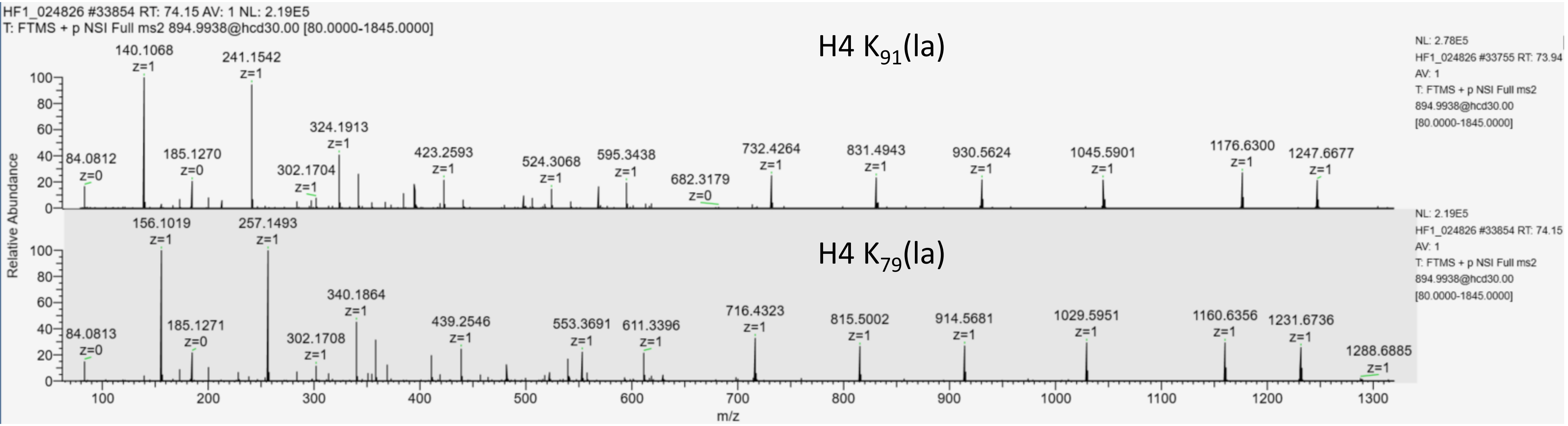

L-la:D-la
1:1

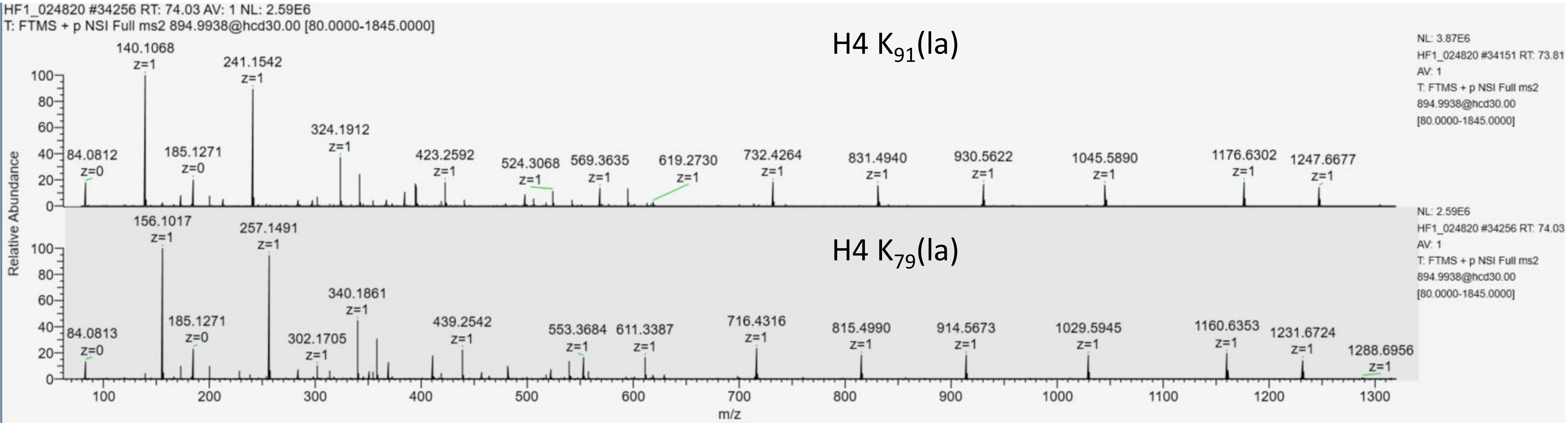

L-la:D-la
1:2

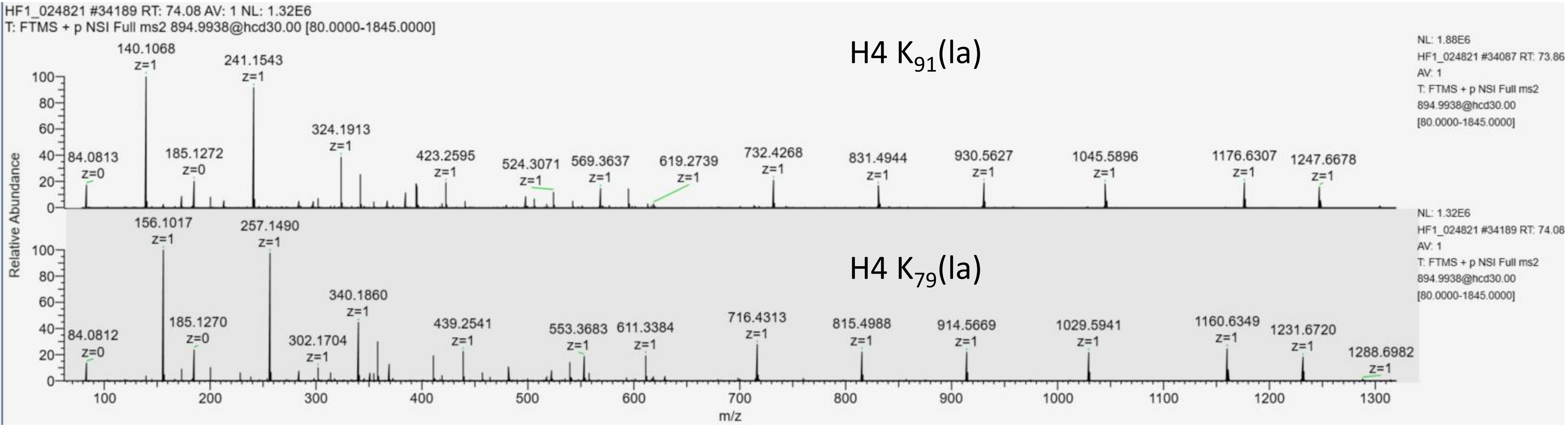
HF1_024821 #34189 RT: 74.08 AV: 1 NL: 1.32E6
T: FTMS + p NSI Full ms2 894.9938@hcd30.00 [80.0000-1845.0000]
H4 K91(la)
NL: 1.88E6
HF1_024821 #34087 RT: 73.86
AV: 1
T: FTMS + p NSI Full ms2
894.9938@hcd30.00
[80.0000-1845.0000]
140.1068 z=1
241.1543 z=1
324.1913 z=1
84.0813 z=0
185.1272 z=0
423.2595 z=1
524.3071 z=1
569.3637 z=1
619.2739 z=1
732.4268 z=1
831.4944 z=1
930.5627 z=1
1045.5896 z=1
1176.6307 z=1
1247.6678 z=1
H4 K79(la)
NL: 1.32E6
HF1_024821 #34189 RT: 74.08
AV: 1
T: FTMS + p NSI Full ms2
894.9938@hcd30.00
[80.0000-1845.0000]
156.1017 z=1
257.1490 z=1
340.1860 z=1
84.0812 z=0
185.1270 z=0
302.1704 z=1
439.2541 z=1
553.3683 z=1
611.3384 z=1
716.4313 z=1
815.4988 z=1
914.5669 z=1
1029.5941 z=1
1160.6349 z=1
1231.6720 z=1
1288.6982 z=1
Relative Abundance
m/z

L-la:D-la
2:1

Extracted ion chromatogram of lactylated H4 K_{79}TVTAM(ox)DVVYALK$_{91}$R (902.9918,2+)

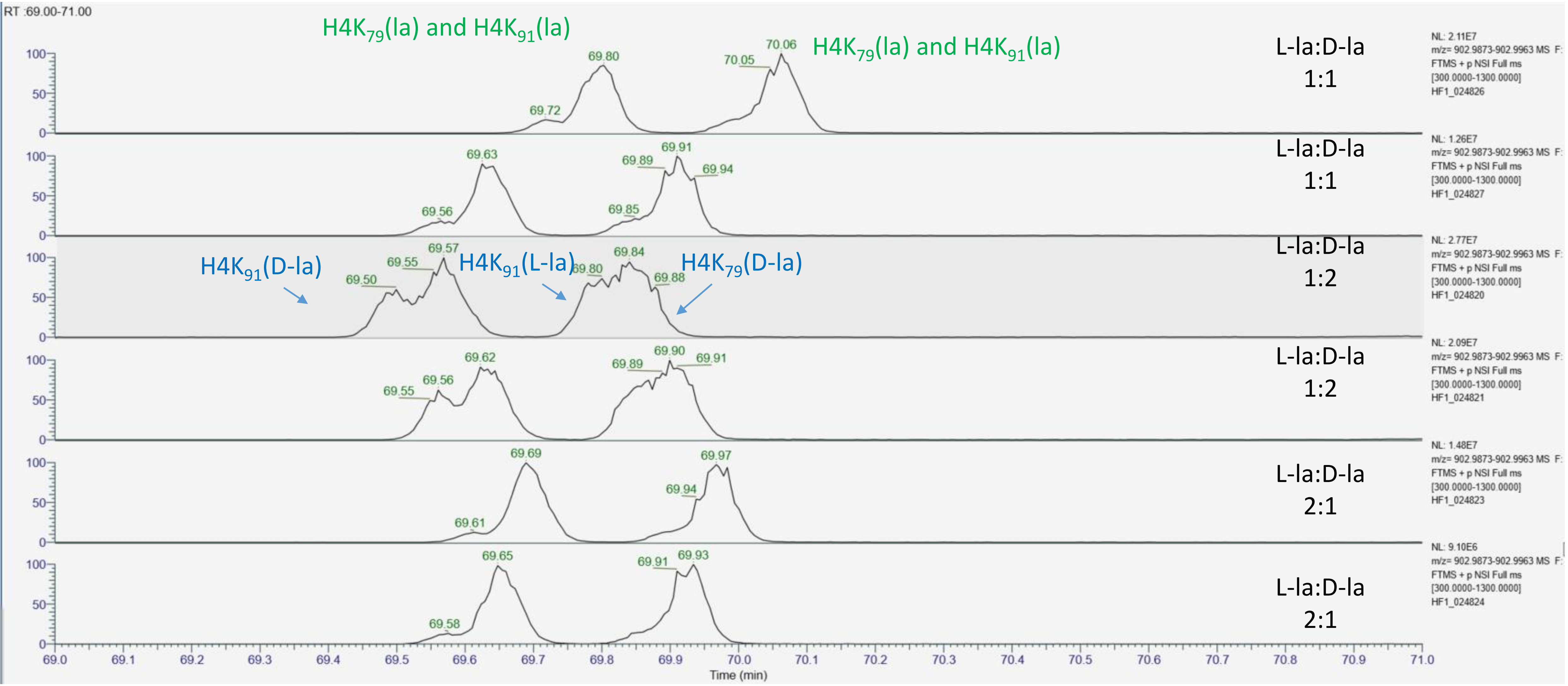

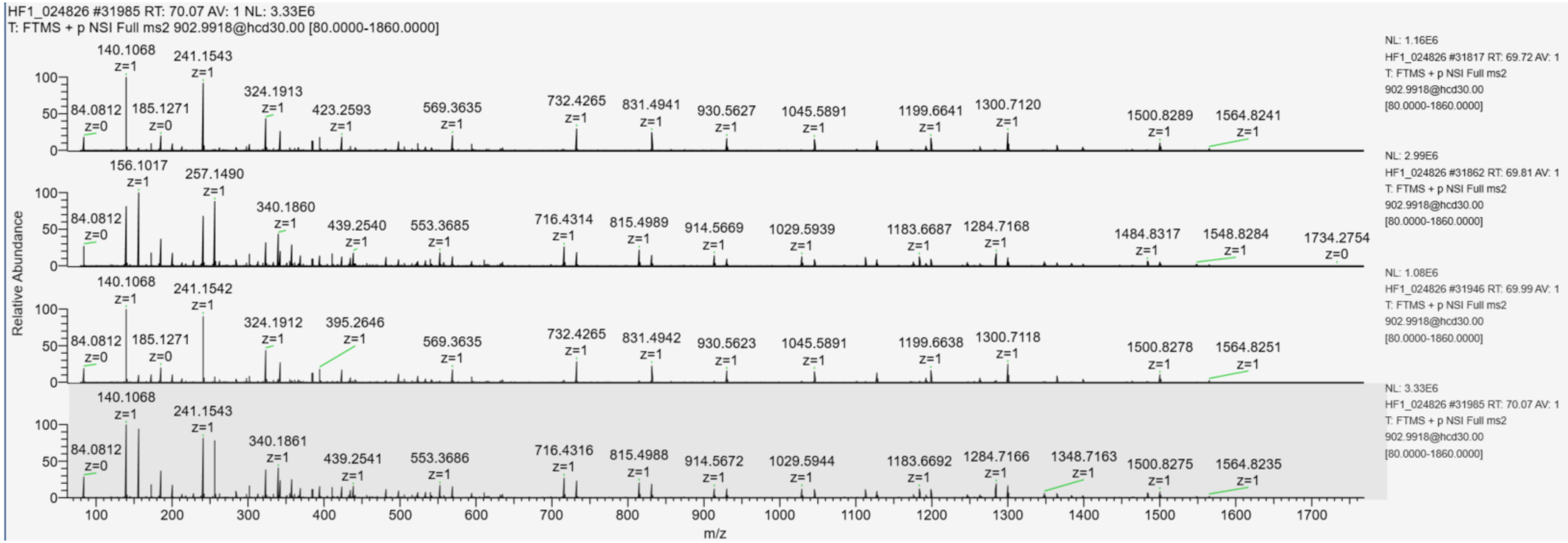

L-la:D-la
1:1

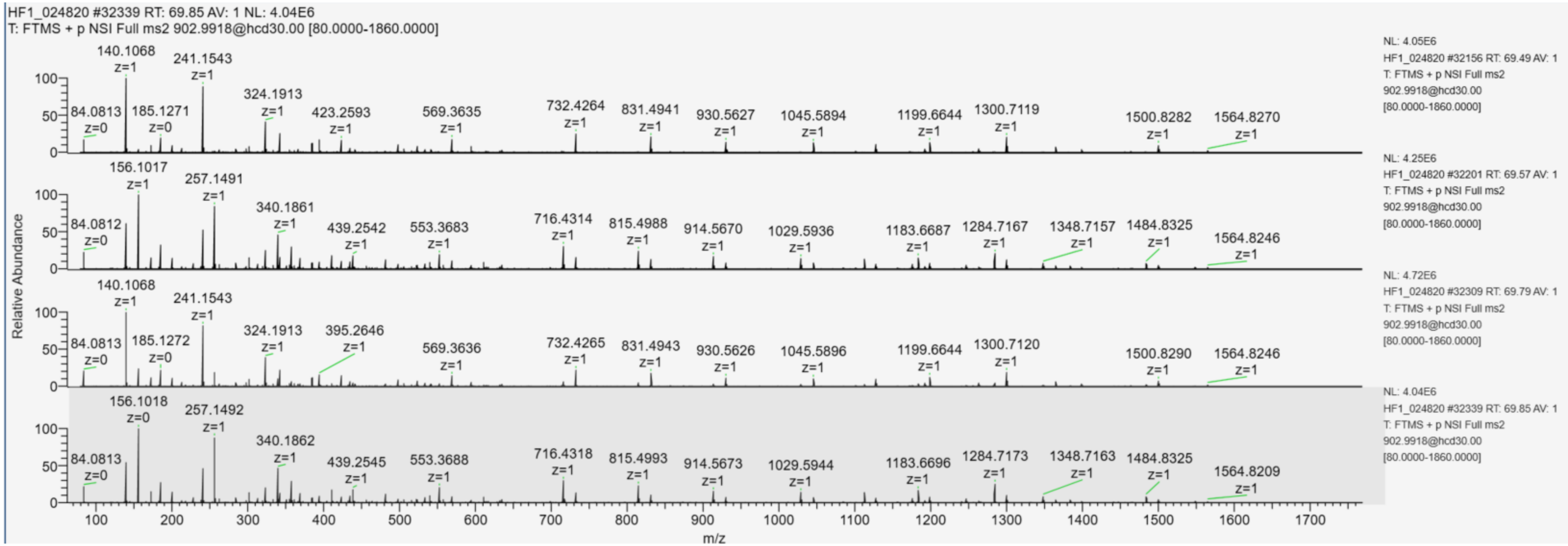

L-la:D-la
1:2

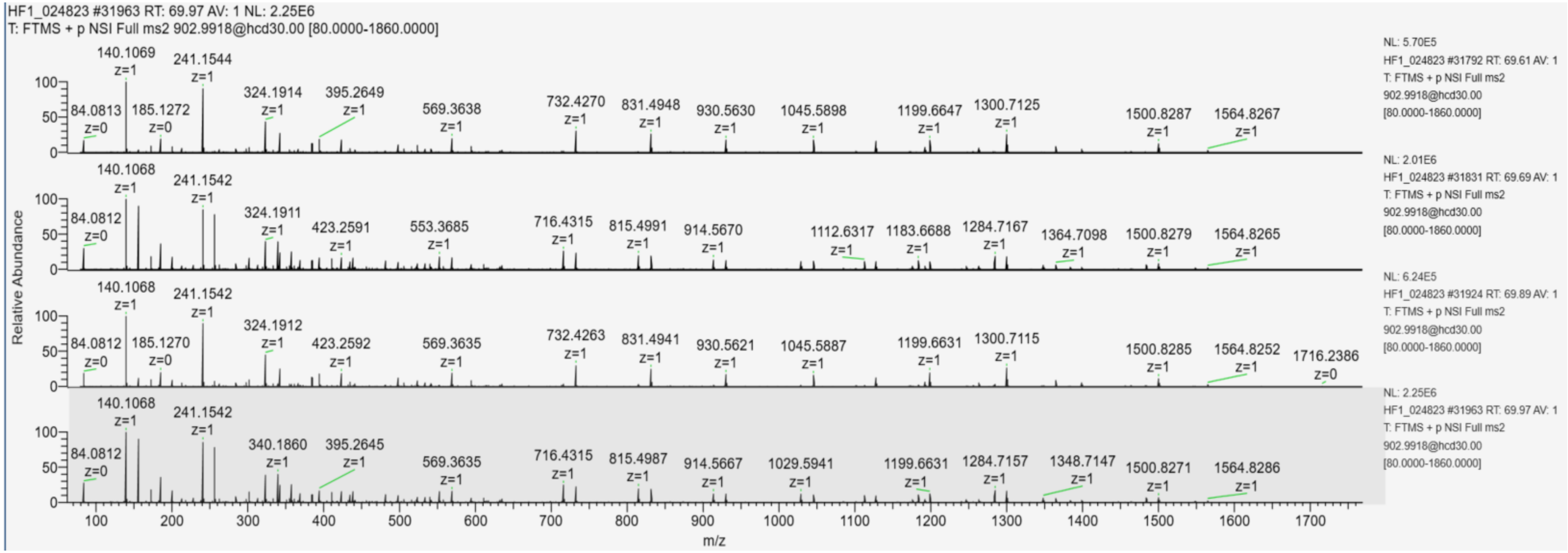
HF1_024823 #31963 RT: 69.97 AV: 1 NL: 2.25E6
T: FTMS + p NSI Full ms2 902.9918@hcd30.00 [80.0000-1860.0000]
Relative Abundance
m/z

L-la:D-la
2:1

Section 4: Statistical assessment of the abundance difference between L- and D-lactylated peptides that are chromatographically separated.

Two approaches were considered to normalize the MS signals detected for lactylated peptides in the analyses acquired on the four biological replicates of testis histones (Suppl. table 10).

Using the program JMP, for each variant and each modified lysine site, we compared:

- the abundance of peptides carrying L-La and D-La blocked by biological replicate (results on the left);
- the abundance of peptides normalized to the sum of abundances of lactylated peptides in each run (results on the right).

The results on the left take into account the effect of biological replicate (assuming that all variability comes from biology). The column on the right normalizes by the injected quantity (assuming that all variability comes from analysis). Reality probably lies in-between.

The results of statistical tests assessing whether L- and D-lactyl are of different abundance on the considered lysine residues are indicated at the bottom of the columns; they appear in red when the test concluded on a significant difference ($\alpha = 0.05$). Different tests indicated in the figures below (e.g. Wilcoxon and Kruskall-Wallis) led to the same conclusions.

H3K18

Oneway Analysis of Data By Modif Variant=H3, Site 2=K18

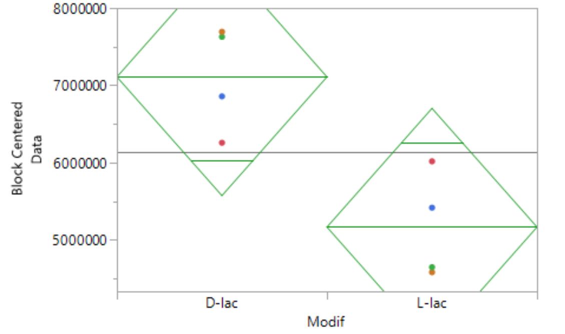

Oneway Anova

Summary of Fit

Rsquare	0.955218
Adj Rsquare	0.895509
Root Mean Square Error	965269.8
Mean of Response	6138750
Observations (or Sum Wgts)	8

Pooled t Test

L-lac-D-lac

Assuming equal variances

Difference	-1942500	t Ratio	-2.84595
Std Err Dif	682549	DF	3
Upper CL Dif	229675	Prob > |t|	0.0653
Lower CL Dif	-4114675	Prob > t	0.9673
Confidence	0.95	Prob < t	0.0327*

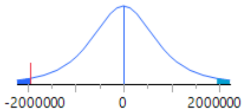

Analysis of Variance

Source	DF	Sum of Squares	Mean Square	F Ratio	Prob > F
Modif	1	7.5466e+12	7.547e+12	8.0994	0.0653
Label	3	5.2077e+13	1.736e+13	18.6307	0.0192*
Error	3	2.7952e+12	9.317e+11		
C. Total	7	6.2419e+13			

Means for Oneway Anova

Level	Number	Mean	Std Error	Lower 95%	Upper 95%
D-lac	4	7110000	482635	5574040.3	8645959.7
L-lac	4	5167500	482635	3631540.3	6703459.7

Std Error uses a pooled estimate of error variance

Block Means

Label	Mean	Number
Abundance 1	2180000	2
Abundance 2	9310000	2
Abundance 3	6530000	2
Abundance 4	6535000	2

Friedman Rank Test

Level	Count	Score Sum	Expected Score	Score Mean	(Mean-Mean0)/Std0
D-lac	4	8.000	6.000	2.00000	1.871
L-lac	4	4.000	6.000	1.00000	-1.871

2 - Sample Test, Normal Approximation

S	Z	Prob>|Z|
4	-1.87083	0.0614

1-Way Test, ChiSquare Approximation

ChiSquare	DF	Prob>ChiSq
4.0000	1	0.0455*

Small sample sizes. Refer to statistical tables for tests, rather than large-sample approximations.

Block Label

Oneway Analysis of Data By Modif Variant=H3, Lys=K18

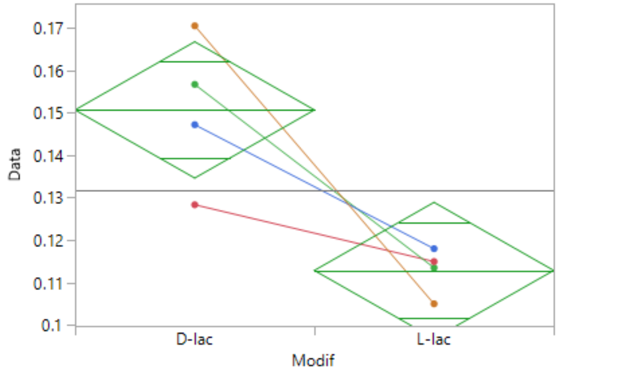

Oneway Anova

Summary of Fit

Rsquare	0.734724
Adj Rsquare	0.690512
Root Mean Square Error	0.013133
Mean of Response	0.131771
Observations (or Sum Wgts)	8

Pooled t Test

L-lac-D-lac

Assuming equal variances

Difference	-0.03786	t Ratio	-4.07652
Std Err Dif	0.00929	DF	6
Upper CL Dif	-0.01513	Prob > |t|	0.0065*
Lower CL Dif	-0.06058	Prob > t	0.9967
Confidence	0.95	Prob < t	0.0033*

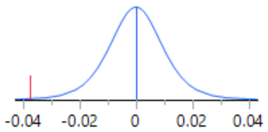

Analysis of Variance

Source	DF	Sum of Squares	Mean Square	F Ratio	Prob > F
Modif	1	0.00286640	0.002866	16.6180	0.0065*
Error	6	0.00103493	0.000172		
C. Total	7	0.00390133			

Means for Oneway Anova

Level	Number	Mean	Std Error	Lower 95%	Upper 95%
D-lac	4	0.150700	0.00657	0.13463	0.16677
L-lac	4	0.112842	0.00657	0.09677	0.12891

Std Error uses a pooled estimate of error variance

Wilcoxon / Kruskal-Wallis Tests (Rank Sums)

Level	Count	Score Sum	Expected Score	Score Mean	(Mean-Mean0)/Std0
D-lac	4	26.000	18.000	6.50000	2.165
L-lac	4	10.000	18.000	2.50000	-2.165

Wilcoxon Two-Sample Test, Normal Approximation

S	Z	Prob>|Z|
10	-2.16506	0.0304*

Kruskal-Wallis Test, ChiSquare Approximation

ChiSquare	DF	Prob>ChiSq
5.3333	1	0.0209*

Small sample sizes. Refer to statistical tables for tests, rather than large-sample approximations.

H3K23

Oneway Analysis of Data By Modif Variant=H3, Site 2=K23

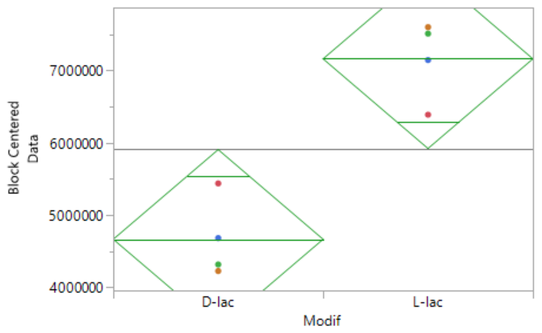

Oneway Anova

Summary of Fit

Rsquare	0.970035
Adj Rsquare	0.930082
Root Mean Square Error	778553.7
Mean of Response	5913750
Observations (or Sum Wgts)	8

Pooled t Test

L-lac-D-lac

Assuming equal variances

Difference	2492500	t Ratio	4.527533
Std Err Dif	550521	DF	3
Upper CL Dif	4244502	Prob > |t|	0.0202*
Lower CL Dif	740498	Prob > t	0.0101*
Confidence	0.95	Prob < t	0.9899

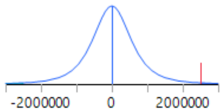

Analysis of Variance

Source	DF	Sum of Squares	Mean Square	F Ratio	Prob > F
Modif	1	1.2425e+13	1.243e+13	20.4986	0.0202*
Label	3	4.6442e+13	1.548e+13	25.5394	0.0123*
Error	3	1.8184e+12	6.061e+11		
C. Total	7	6.0685e+13			

Means for Oneway Anova

Level	Number	Mean	Std Error	Lower 95%	Upper 95%
D-lac	4	4667500	389277	3428647.4	5906352.6
L-lac	4	7160000	389277	5921147.4	8398852.6

Std Error uses a pooled estimate of error variance

Block Means

Label	Mean	Number
Abundance 1	2315000	2
Abundance 2	9105000	2
Abundance 3	6080000	2
Abundance 4	6155000	2

Friedman Rank Test

Level	Count	Score Sum	Expected Score	Score Mean	(Mean-Mean0)/Std0
D-lac	4	4.000	6.000	1.00000	-1.871
L-lac	4	8.000	6.000	2.00000	1.871

2 - Sample Test, Normal Approximation

S	Z	Prob>|Z|
8	1.87083	0.0614

1-Way Test, ChiSquare Approximation

ChiSquare	DF	Prob>ChiSq
4.0000	1	0.0455*

Small sample sizes. Refer to statistical tables for tests, rather than large-sample approximations.

Block Label

Oneway Analysis of Data By Modif Variant=H3, Lys=K23

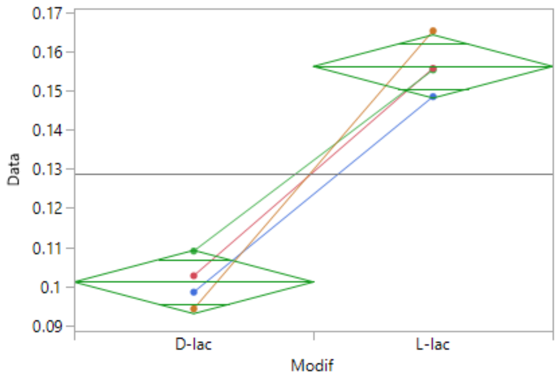

Oneway Anova

Summary of Fit

Rsquare	0.958572
Adj Rsquare	0.951667
Root Mean Square Error	0.00661
Mean of Response	0.128624
Observations (or Sum Wgts)	8

Pooled t Test

L-lac-D-lac

Assuming equal variances

Difference	0.055072	t Ratio	11.78254
Std Err Dif	0.004674	DF	6
Upper CL Dif	0.066508	Prob > |t|	<.0001*
Lower CL Dif	0.043635	Prob > t	<.0001*
Confidence	0.95	Prob < t	1.0000

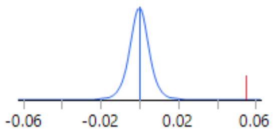

Analysis of Variance

Source	DF	Sum of Squares	Mean Square	F Ratio	Prob > F
Modif	1	0.00606576	0.006066	138.8284	<.0001*
Error	6	0.00026216	0.000044		
C. Total	7	0.00632792			

Means for Oneway Anova

Level	Number	Mean	Std Error	Lower 95%	Upper 95%
D-lac	4	0.101088	0.00331	0.09300	0.10917
L-lac	4	0.156159	0.00331	0.14807	0.16425

Std Error uses a pooled estimate of error variance

Wilcoxon / Kruskal-Wallis Tests (Rank Sums)

Level	Count	Score Sum	Expected Score	Score Mean	(Mean-Mean0)/Std0
D-lac	4	10.000	18.000	2.50000	-2.165
L-lac	4	26.000	18.000	6.50000	2.165

Wilcoxon Two-Sample Test, Normal Approximation

S	Z	Prob>|Z|
26	2.16506	0.0304*

Kruskal-Wallis Test, ChiSquare Approximation

ChiSquare	DF	Prob>ChiSq
5.3333	1	0.0209*

Small sample sizes. Refer to statistical tables for tests, rather than large-sample approximations.

H3.tK18

Oneway Analysis of Data By Modif Variant=H3.t, Site 2=K18

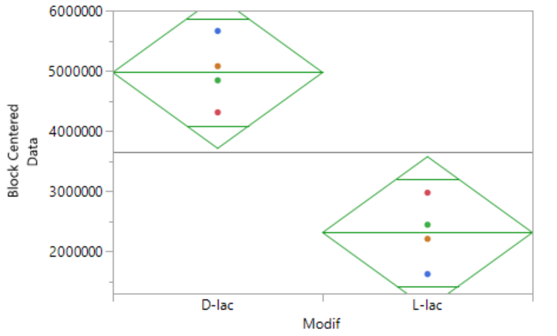

Oneway Anova

Summary of Fit

Rsquare	0.943242
Adj Rsquare	0.867565
Root Mean Square Error	792855.5
Mean of Response	3641875
Observations (or Sum Wgts)	8

Pooled t Test

L-lac-D-lac

Assuming equal variances

Difference	-2661250	t Ratio	-4.74686
Std Err Dif	560633	DF	3
Upper CL Dif	-877064	Prob > |t|	0.0177*
Lower CL Dif	-4445436	Prob > t	0.9911
Confidence	0.95	Prob < t	0.0089*

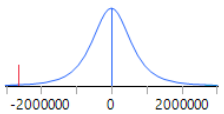

Analysis of Variance

Source	DF	Sum of Squares	Mean Square	F Ratio	Prob > F
Modif	1	1.4165e+13	1.416e+13	22.5327	0.0177*
Label	3	1.7176e+13	5.725e+12	9.1079	0.0512
Error	3	1.8859e+12	6.286e+11		
C. Total	7	3.3226e+13			

Means for Oneway Anova

Level	Number	Mean	Std Error	Lower 95%	Upper 95%
D-lac	4	4972500	396428	3710890	6234110
L-lac	4	2311250	396428	1049640	3572860

Std Error uses a pooled estimate of error variance

Block Means

Label	Mean	Number
Abundance 1	1492500	2
Abundance 2	5620000	2
Abundance 3	3870000	2
Abundance 4	3585000	2

Friedman Rank Test

Level	Count	Score Sum	Expected Score	Score Mean	(Mean-Mean0)/Std0
D-lac	4	8.000	6.000	2.00000	1.871
L-lac	4	4.000	6.000	1.00000	-1.871

2 - Sample Test, Normal Approximation

S	Z	Prob>|Z|
4	-1.87083	0.0614

1-Way Test, ChiSquare Approximation

ChiSquare	DF	Prob>ChiSq
4.0000	1	0.0455*

Small sample sizes. Refer to statistical tables for tests, rather than large-sample approximations.

Block Label

Oneway Analysis of Data By Modif Variant=H3.t, Lys=K18

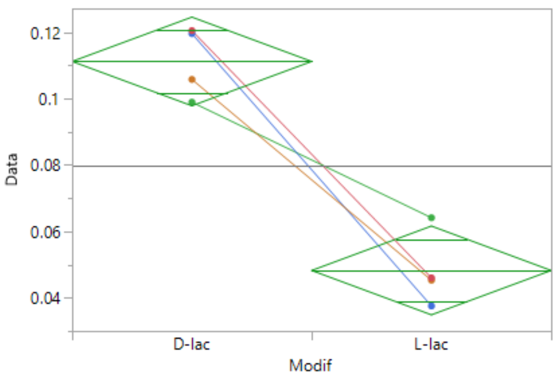

Oneway Anova

Summary of Fit

Rsquare	0.917146
Adj Rsquare	0.903337
Root Mean Square Error	0.010926
Mean of Response	0.079745
Observations (or Sum Wgts)	8

Pooled t Test

L-lac-D-lac

Assuming equal variances

Difference	-0.06296	t Ratio	-8.14964
Std Err Dif	0.00773	DF	6
Upper CL Dif	-0.04406	Prob > |t|	0.0002*
Lower CL Dif	-0.08187	Prob > t	0.9999
Confidence	0.95	Prob < t	<.0001*

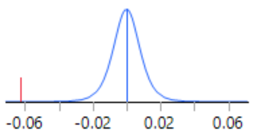

Analysis of Variance

Source	DF	Sum of Squares	Mean Square	F Ratio	Prob > F
Modif	1	0.00792845	0.007928	66.4166	0.0002*
Error	6	0.00071625	0.000119		
C. Total	7	0.00864470			

Means for Oneway Anova

Level	Number	Mean	Std Error	Lower 95%	Upper 95%
D-lac	4	0.111226	0.00546	0.09786	0.12459
L-lac	4	0.048264	0.00546	0.03490	0.06163

Std Error uses a pooled estimate of error variance

Wilcoxon / Kruskal-Wallis Tests (Rank Sums)

Level	Count	Score Sum	Expected Score	Score Mean	(Mean-Mean0)/Std0
D-lac	4	26.000	18.000	6.50000	2.165
L-lac	4	10.000	18.000	2.50000	-2.165

Wilcoxon Two-Sample Test, Normal Approximation

S	Z	Prob>|Z|
10	-2.16506	0.0304*

Kruskal-Wallis Test, ChiSquare Approximation

ChiSquare	DF	Prob>ChiSq
5.3333	1	0.0209*

Small sample sizes. Refer to statistical tables for tests, rather than large-sample approximations.

H3.tK23

Oneway Analysis of Data By Modif Variant=H3.t, Site 2=K23

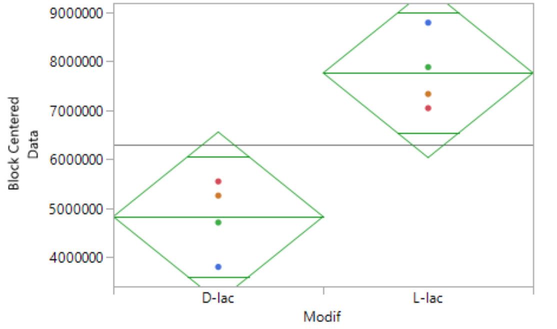

Oneway Anova

Summary of Fit

Rsquare	0.945756
Adj Rsquare	0.873431
Root Mean Square Error	1088914
Mean of Response	6295000
Observations (or Sum Wgts)	8

Pooled t Test

L-lac-D-lac

Assuming equal variances

Difference	2940000	t Ratio	3.818289
Std Err Dif	769978	DF	3
Upper CL Dif	5390415	Prob > |t|	0.0316*
Lower CL Dif	489585	Prob > t	0.0158*
Confidence	0.95	Prob < t	0.9842

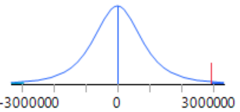

Analysis of Variance

Source	DF	Sum of Squares	Mean Square	F Ratio	Prob > F
Modif	1	1.7287e+13	1.729e+13	14.5793	0.0316*
Label	3	4.4733e+13	1.491e+13	12.5755	0.0332*
Error	3	3.5572e+12	1.186e+12		
C. Total	7	6.5578e+13			

Means for Oneway Anova

Level	Number	Mean	Std Error	Lower 95%	Upper 95%
D-lac	4	4825000	544457	3092295.1	6557704.9
L-lac	4	7765000	544457	6032295.1	9497704.9

Std Error uses a pooled estimate of error variance

Block Means

Label	Mean	Number
Abundance 1	2510000	2
Abundance 2	9010000	2
Abundance 3	7050000	2
Abundance 4	6610000	2

Friedman Rank Test

Level	Count	Score Sum	Expected Score	Score Mean	(Mean-Mean0)/Std0
D-lac	4	4.000	6.000	1.00000	-1.871
L-lac	4	8.000	6.000	2.00000	1.871

2 - Sample Test, Normal Approximation

S	Z	Prob>|Z|
8	1.87083	0.0614

1-Way Test, ChiSquare Approximation

ChiSquare	DF	Prob>ChiSq
4.0000	1	0.0455*

Small sample sizes. Refer to statistical tables for tests, rather than large-sample approximations.

Block Label

Oneway Analysis of Data By Modif Variant=H3.t, Lys=K23

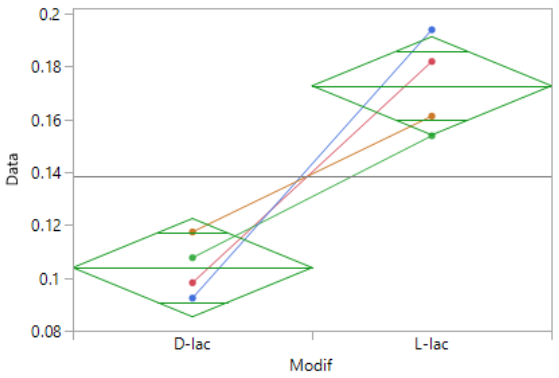

Oneway Anova

Summary of Fit

Rsquare	0.872447
Adj Rsquare	0.851189
Root Mean Square Error	0.015189
Mean of Response	0.138321
Observations (or Sum Wgts)	8

Pooled t Test

L-lac-D-lac

Assuming equal variances

Difference	0.068804	t Ratio	6.4062
Std Err Dif	0.010740	DF	6
Upper CL Dif	0.095084	Prob > |t|	0.0007*
Lower CL Dif	0.042523	Prob > t	0.0003*
Confidence	0.95	Prob < t	0.9997

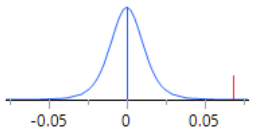

Analysis of Variance

Source	DF	Sum of Squares	Mean Square	F Ratio	Prob > F
Modif	1	0.00946787	0.009468	41.0394	0.0007*
Error	6	0.00138421	0.000231		
C. Total	7	0.01085208			

Means for Oneway Anova

Level	Number	Mean	Std Error	Lower 95%	Upper 95%
D-lac	4	0.103919	0.00759	0.08534	0.12250
L-lac	4	0.172723	0.00759	0.15414	0.19131

Std Error uses a pooled estimate of error variance

Wilcoxon / Kruskal-Wallis Tests (Rank Sums)

Level	Count	Score Sum	Expected Score	Score Mean	(Mean-Mean0)/Std0
D-lac	4	10.000	18.000	2.50000	-2.165
L-lac	4	26.000	18.000	6.50000	2.165

Wilcoxon Two-Sample Test, Normal Approximation

S	Z	Prob>|Z|
26	2.16506	0.0304*

Kruskal-Wallis Test, ChiSquare Approximation

ChiSquare	DF	Prob>ChiSq
5.3333	1	0.0209*

Small sample sizes. Refer to statistical tables for tests, rather than large-sample approximations.

H3K56

Oneway Analysis of Data By Modif Variant=H3, Site 2=K56

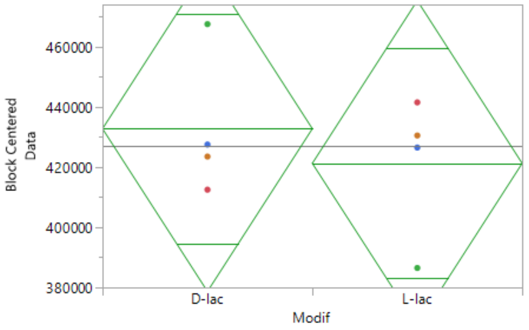

Oneway Anova

Summary of Fit

Rsquare	0.986129
Adj Rsquare	0.967634
Root Mean Square Error	33968.12
Mean of Response	427000
Observations (or Sum Wgts)	8

Pooled t Test

L-lac-D-lac

Assuming equal variances

Difference	-11500	t Ratio	-0.47879
Std Err Dif	24019	DF	3
Upper CL Dif	64939	Prob > |t|	0.6648
Lower CL Dif	-87939	Prob > t	0.6676
Confidence	0.95	Prob < t	0.3324

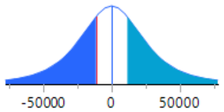

Analysis of Variance

Source	DF	Sum of Squares	Mean Square	F Ratio	Prob > F
Modif	1	264500000	264500000	0.2292	0.6648
Label	3	2.4582e+11	8.194e+10	71.0149	0.0028*
Error	3	3461500000	1.1538e+9		
C. Total	7	2.4954e+11			

Means for Oneway Anova

Level	Number	Mean	Std Error	Lower 95%	Upper 95%
D-lac	4	432750	16984	378699	486801
L-lac	4	421250	16984	367199	475301

Std Error uses a pooled estimate of error variance

Block Means

Label	Mean	Number
Abundance 1	187500	2
Abundance 2	677500	2
Abundance 3	458500	2
Abundance 4	384500	2

Friedman Rank Test

Level	Count	Score Sum	Expected Score	Score Mean	(Mean-Mean0)/Std0
D-lac	4	6.000	6.000	1.50000	0.000
L-lac	4	6.000	6.000	1.50000	0.000

2 - Sample Test, Normal Approximation

S	Z	Prob>|Z|
6	0.00000	1.0000

1-Way Test, ChiSquare Approximation

ChiSquare	DF	Prob>ChiSq
0.0000	1	1.0000

Small sample sizes. Refer to statistical tables for tests, rather than large-sample approximations.

Block Label

Oneway Analysis of Data By Modif Variant=H3, Lys=K56

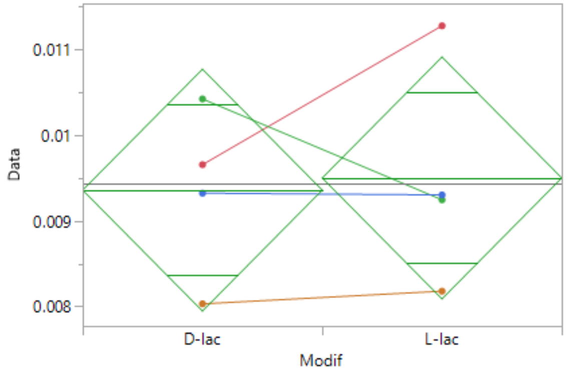

Oneway Anova

Summary of Fit

Rsquare	0.005075
Adj Rsquare	-0.16075
Root Mean Square Error	0.001152
Mean of Response	0.009427
Observations (or Sum Wgts)	8

Pooled t Test

L-lac-D-lac

Assuming equal variances

Difference	0.00014	t Ratio	0.174938
Std Err Dif	0.00081	DF	6
Upper CL Dif	0.00214	Prob > |t|	0.8669
Lower CL Dif	-0.00185	Prob > t	0.4334
Confidence	0.95	Prob < t	0.5666

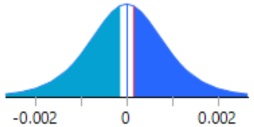

Analysis of Variance

Source	DF	Sum of Squares	Mean Square	F Ratio	Prob > F
Modif	1	4.06116e-8	4.0612e-8	0.0306	0.8669
Error	6	7.96222e-6	1.327e-6		
C. Total	7	8.00283e-6			

Means for Oneway Anova

Level	Number	Mean	Std Error	Lower 95%	Upper 95%
D-lac	4	0.009356	0.00058	0.00795	0.01077
L-lac	4	0.009498	0.00058	0.00809	0.01091

Std Error uses a pooled estimate of error variance

Wilcoxon / Kruskal-Wallis Tests (Rank Sums)

Level	Count	Score Sum	Expected Score	Score Mean	(Mean-Mean0)/Std0
D-lac	4	19.000	18.000	4.75000	0.144
L-lac	4	17.000	18.000	4.25000	-0.144

Wilcoxon Two-Sample Test, Normal Approximation

S	Z	Prob>|Z|
17	-0.14434	0.8852

Kruskal-Wallis Test, ChiSquare Approximation

ChiSquare	DF	Prob>ChiSq
0.0833	1	0.7728

Small sample sizes. Refer to statistical tables for tests, rather than large-sample approximations.

H4K77

Oneway Analysis of Data By Modif Variant=H4, Site 2=K77

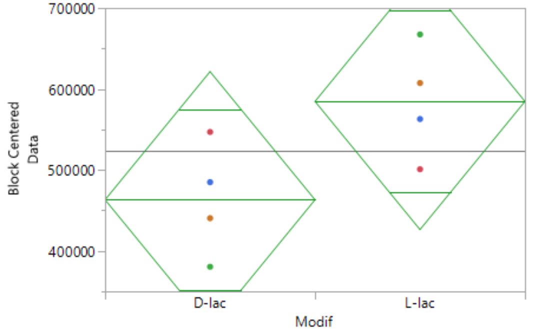

Oneway Anova

Summary of Fit

Rsquare	0.903023
Adj Rsquare	0.77372
Root Mean Square Error	99506.28
Mean of Response	524000
Observations (or Sum Wgts)	8

Pooled t Test

L-lac-D-lac
Assuming equal variances

Difference	121500	t Ratio	1.726795
Std Err Dif	70362	DF	3
Upper CL Dif	345422	Prob > |t|	0.1827
Lower CL Dif	-102422	Prob > t	0.0913
Confidence	0.95	Prob < t	0.9087

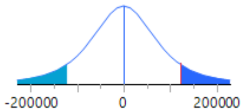

Analysis of Variance

Source	DF	Sum of Squares	Mean Square	F Ratio	Prob > F
Modif	1	2.9524e+10	2.952e+10	2.9818	0.1827
Label	3	2.4708e+11	8.236e+10	8.3178	0.0577
Error	3	2.9704e+10	9.9015e+9		
C. Total	7	3.063e+11			

Means for Oneway Anova

Level	Number	Mean	Std Error	Lower 95%	Upper 95%
D-lac	4	463250	49753	304913	621587
L-lac	4	584750	49753	426413	743087

Std Error uses a pooled estimate of error variance

Block Means

Label	Mean	Number
Abundance 1	276000	2
Abundance 2	734500	2
Abundance 3	635000	2
Abundance 4	450500	2

Friedman Rank Test

Level	Count	Score Sum	Expected Score	Score Mean	(Mean-Mean0)/Std0
D-lac	4	5.000	6.000	1.25000	-0.935
L-lac	4	7.000	6.000	1.75000	0.935

2 - Sample Test, Normal Approximation

S	Z	Prob>|Z|
7	0.93541	0.3496

1-Way Test, ChiSquare Approximation

ChiSquare	DF	Prob>ChiSq
1.0000	1	0.3173

Small sample sizes. Refer to statistical tables for tests, rather than large-sample approximations.

Block Label

Oneway Analysis of Data By Modif Variant=H4, Lys=K77

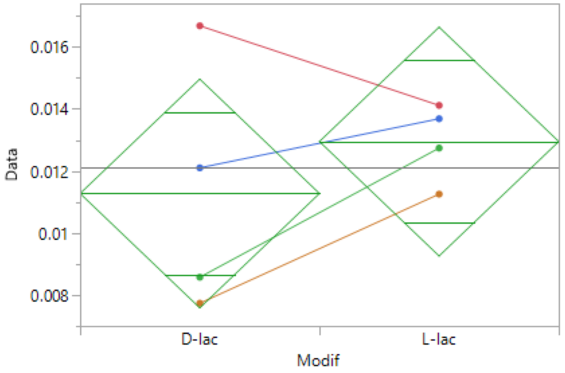

Oneway Anova

Summary of Fit

Rsquare	0.093306
Adj Rsquare	-0.05781
Root Mean Square Error	0.003016
Mean of Response	0.012112
Observations (or Sum Wgts)	8

Pooled t Test

L-lac-D-lac
Assuming equal variances

Difference	0.00168	t Ratio	0.785779
Std Err Dif	0.00213	DF	6
Upper CL Dif	0.00689	Prob > |t|	0.4619
Lower CL Dif	-0.00354	Prob > t	0.2309
Confidence	0.95	Prob < t	0.7691

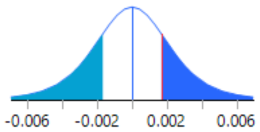

Analysis of Variance

Source	DF	Sum of Squares	Mean Square	F Ratio	Prob > F
Modif	1	0.00000561	5.6146e-6	0.6174	0.4619
Error	6	0.00005456	9.0933e-6		
C. Total	7	0.00006017			

Means for Oneway Anova

Level	Number	Mean	Std Error	Lower 95%	Upper 95%
D-lac	4	0.011274	0.00151	0.00759	0.01496
L-lac	4	0.012950	0.00151	0.00926	0.01664

Std Error uses a pooled estimate of error variance

Wilcoxon / Kruskal-Wallis Tests (Rank Sums)

Level	Count	Score Sum	Expected Score	Score Mean	(Mean-Mean0)/Std0
D-lac	4	15.000	18.000	3.75000	-0.722
L-lac	4	21.000	18.000	5.25000	0.722

Wilcoxon Two-Sample Test, Normal Approximation

S	Z	Prob>|Z|
21	0.72169	0.4705

Kruskal-Wallis Test, ChiSquare Approximation

ChiSquare	DF	Prob>ChiSq
0.7500	1	0.3865

Small sample sizes. Refer to statistical tables for tests, rather than large-sample approximations.

Section 5: Reason why H4K31la could not be quantified in the four biological replicates of mouse testis histones

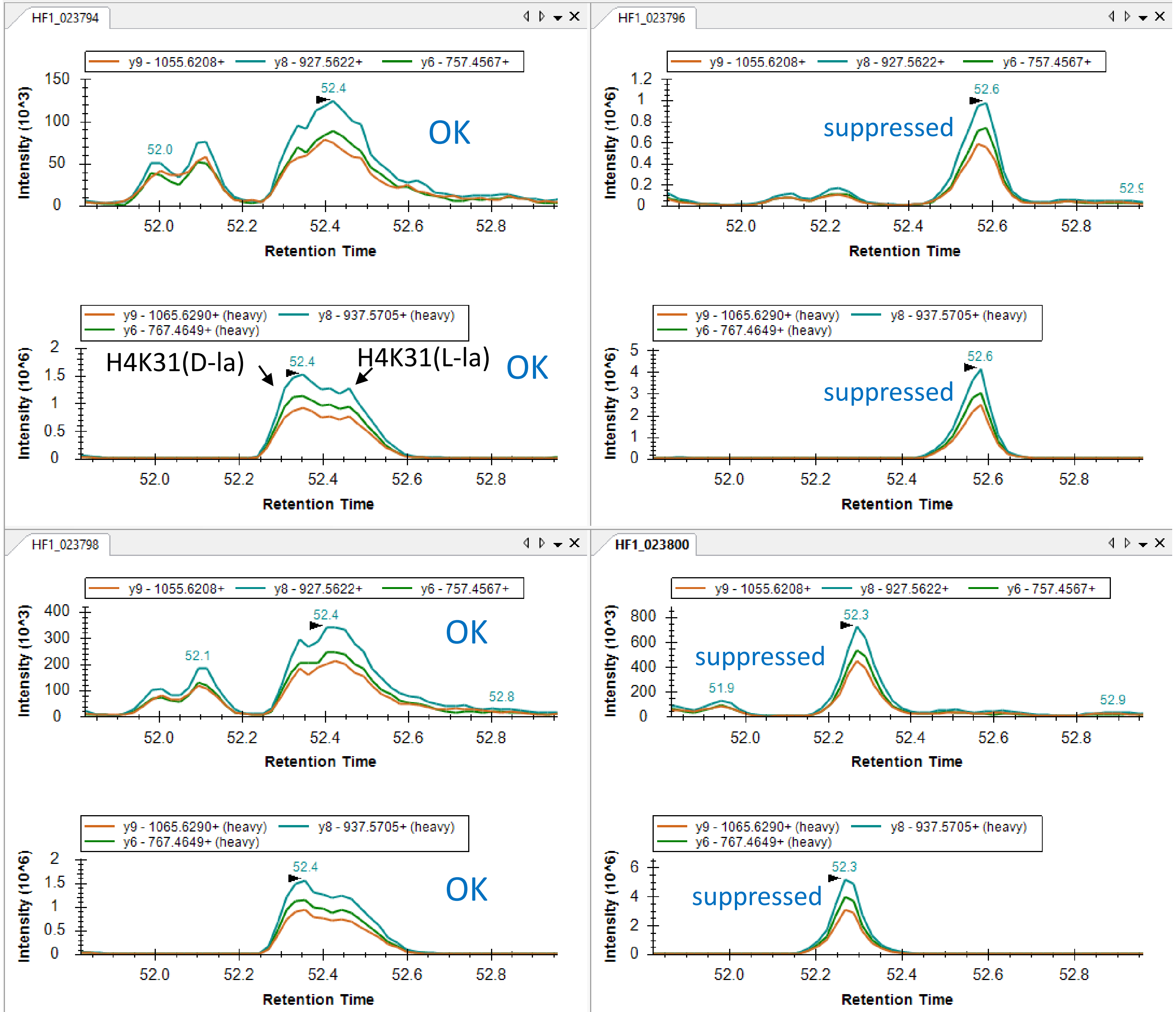

Detection of MS/MS signals for heavy and endogenous DNIQGITK31(la)PAIR is hampered by another abundant species in two analyses out of the four.

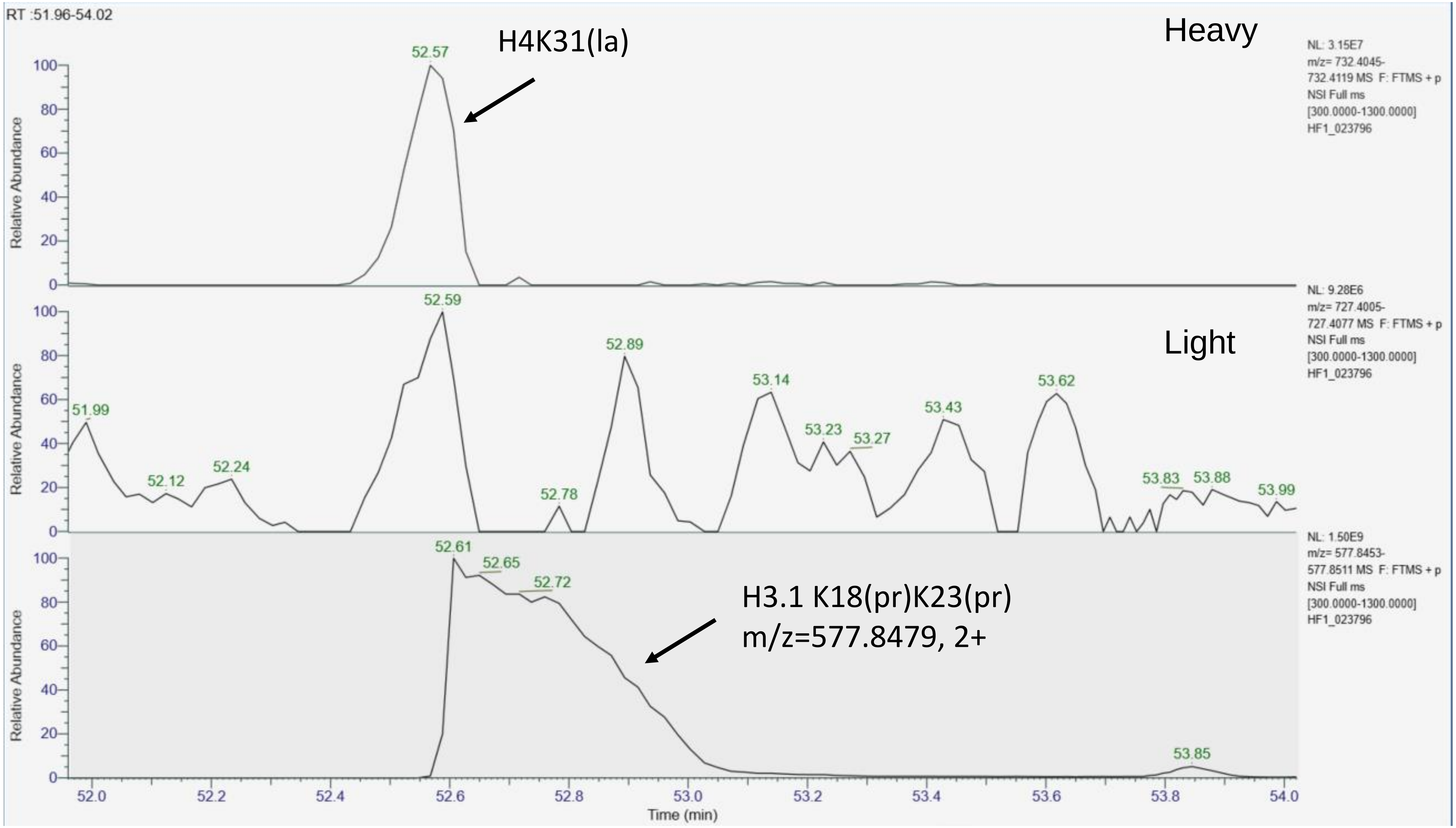

The interfering peptide is probably the highly intense (and abundant) peptide H3 K18prQLATK23prAAR.

Section 6: MS2 signals for endogenous and heavy H3K79la-containing peptides show that the endogenous peptide essentially matches the D-lactylated synthetic peptide

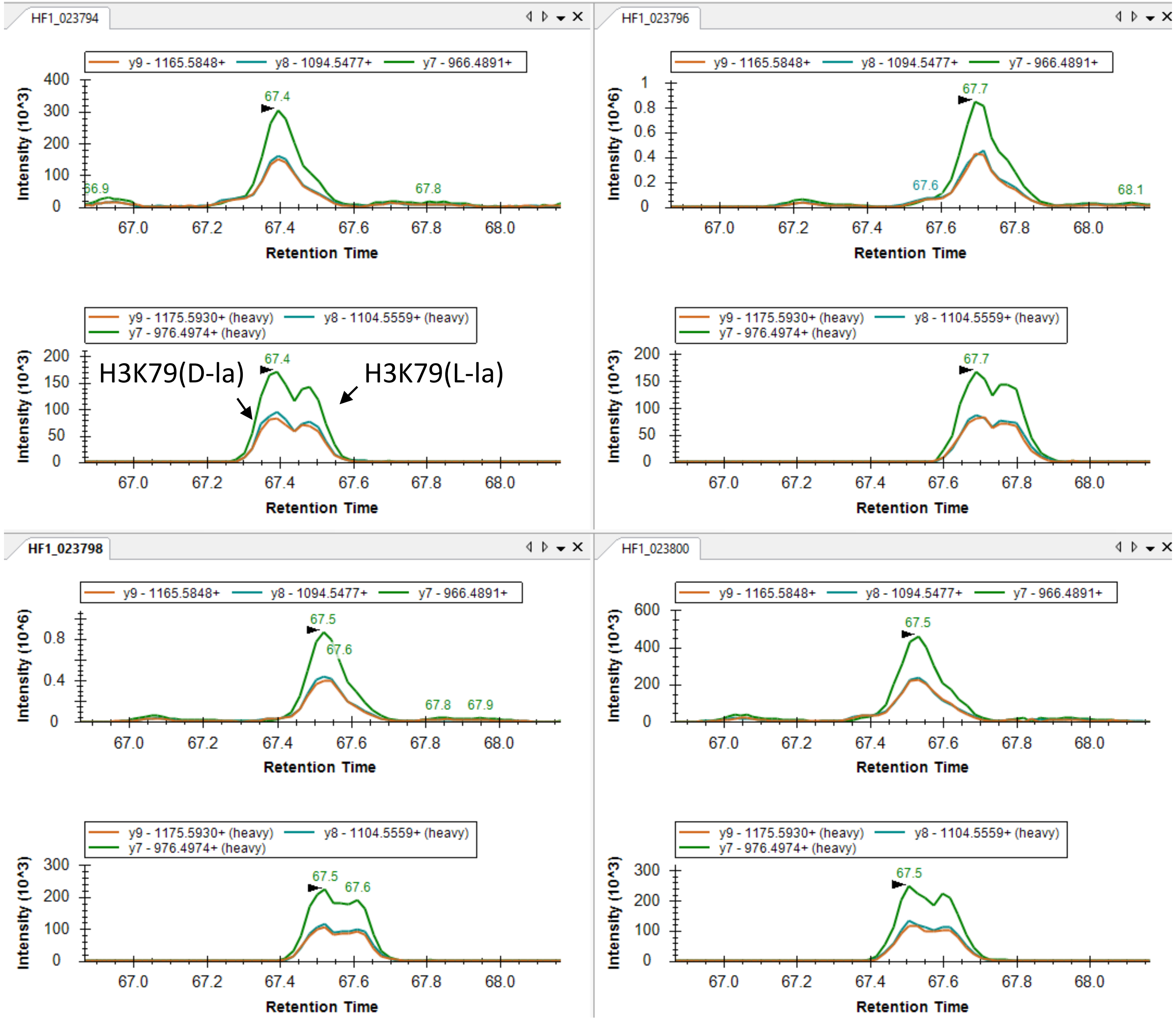

Section 7: Comparison of the retention times of acetylated peptides and their lactylated counterparts

Retention time of synthetic acetylated peptides versus D-lactylated peptides of H3 and H4

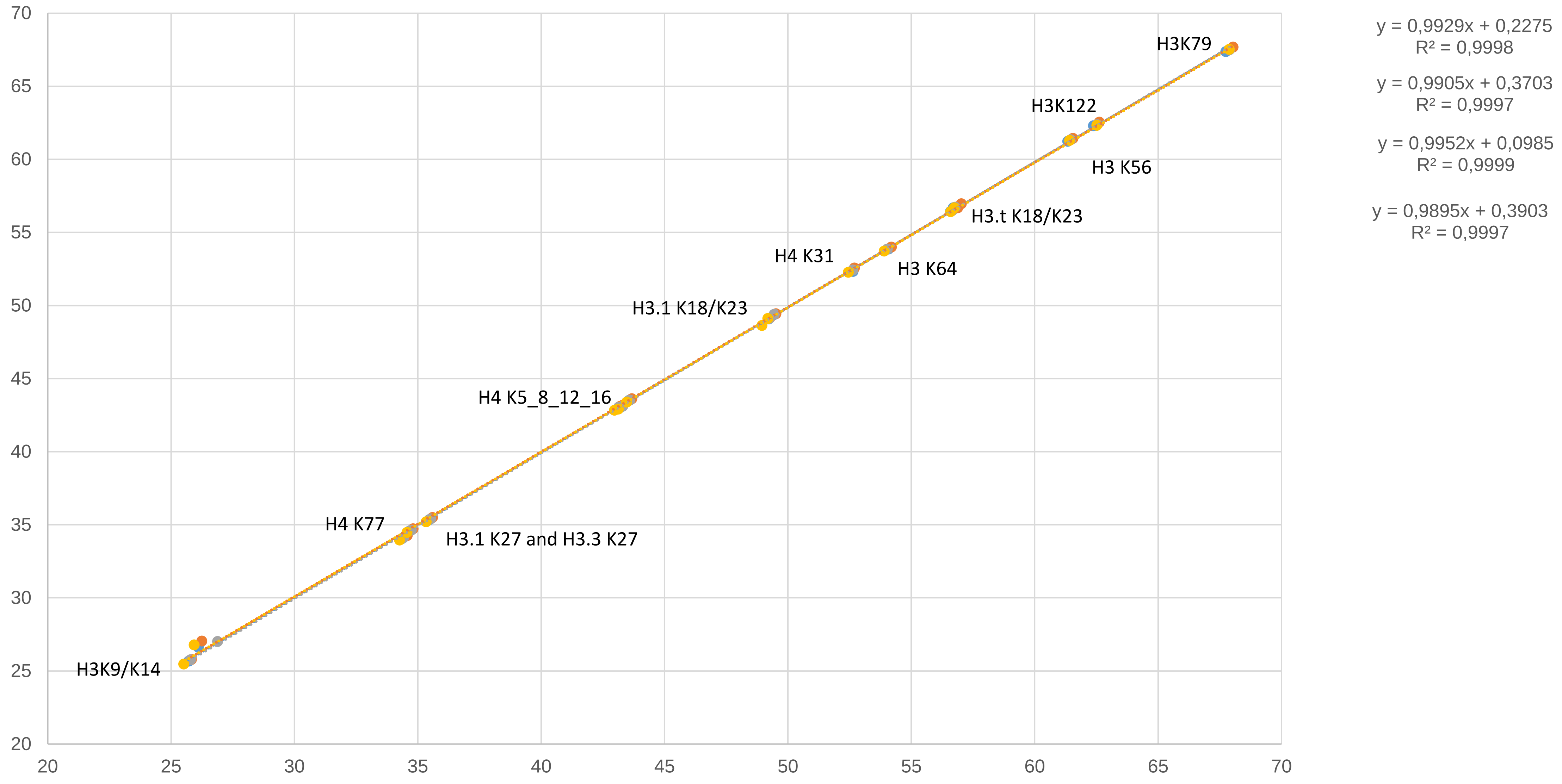

Four technical replicate injections were performed.

Retention time of synthetic acetylated peptides versus L-lactylated peptides of H3 and H4

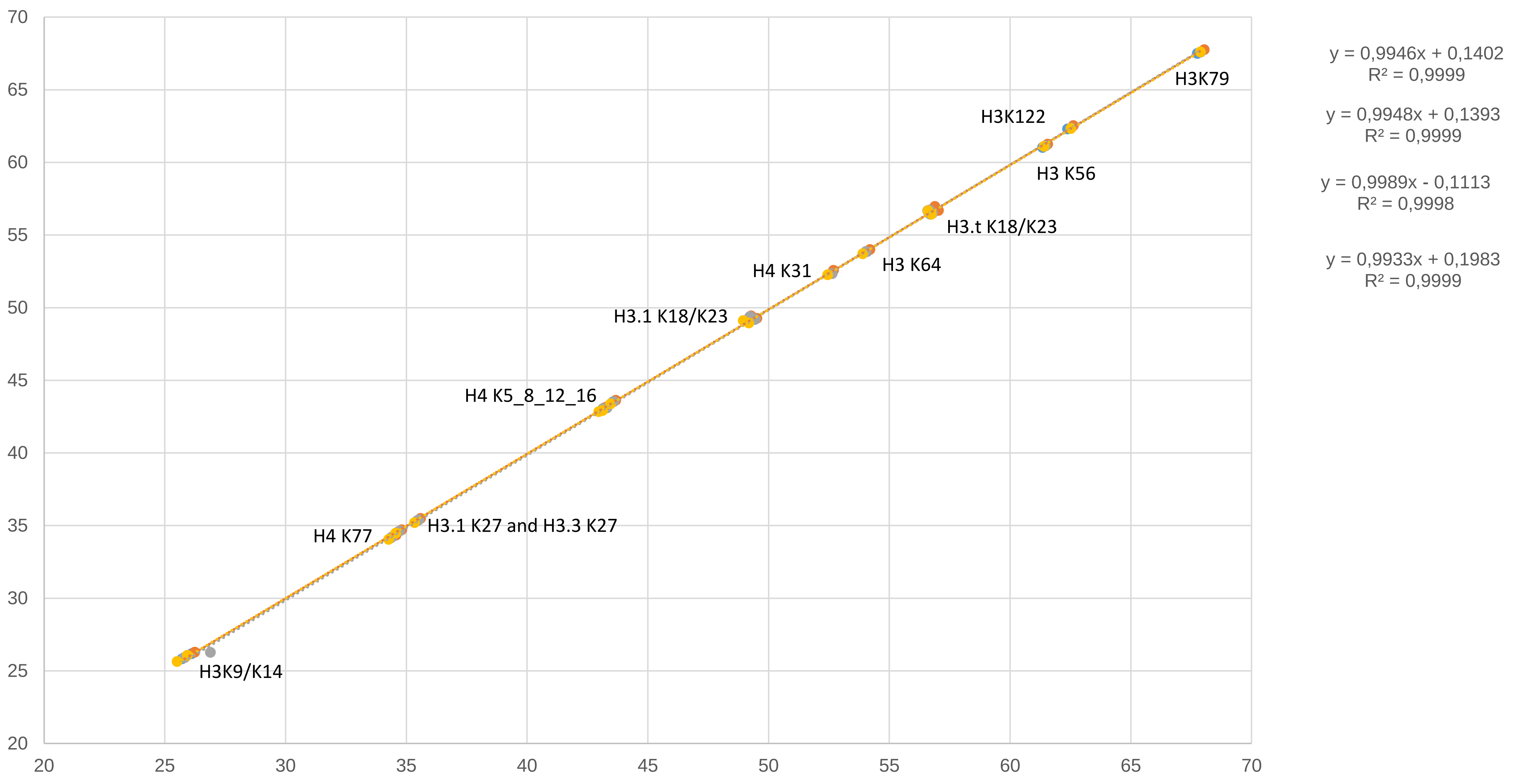

Four technical replicate injections were performed.

Section 8: Comparison of the abundances of acetylated peptides and their lactylated counterparts on H4

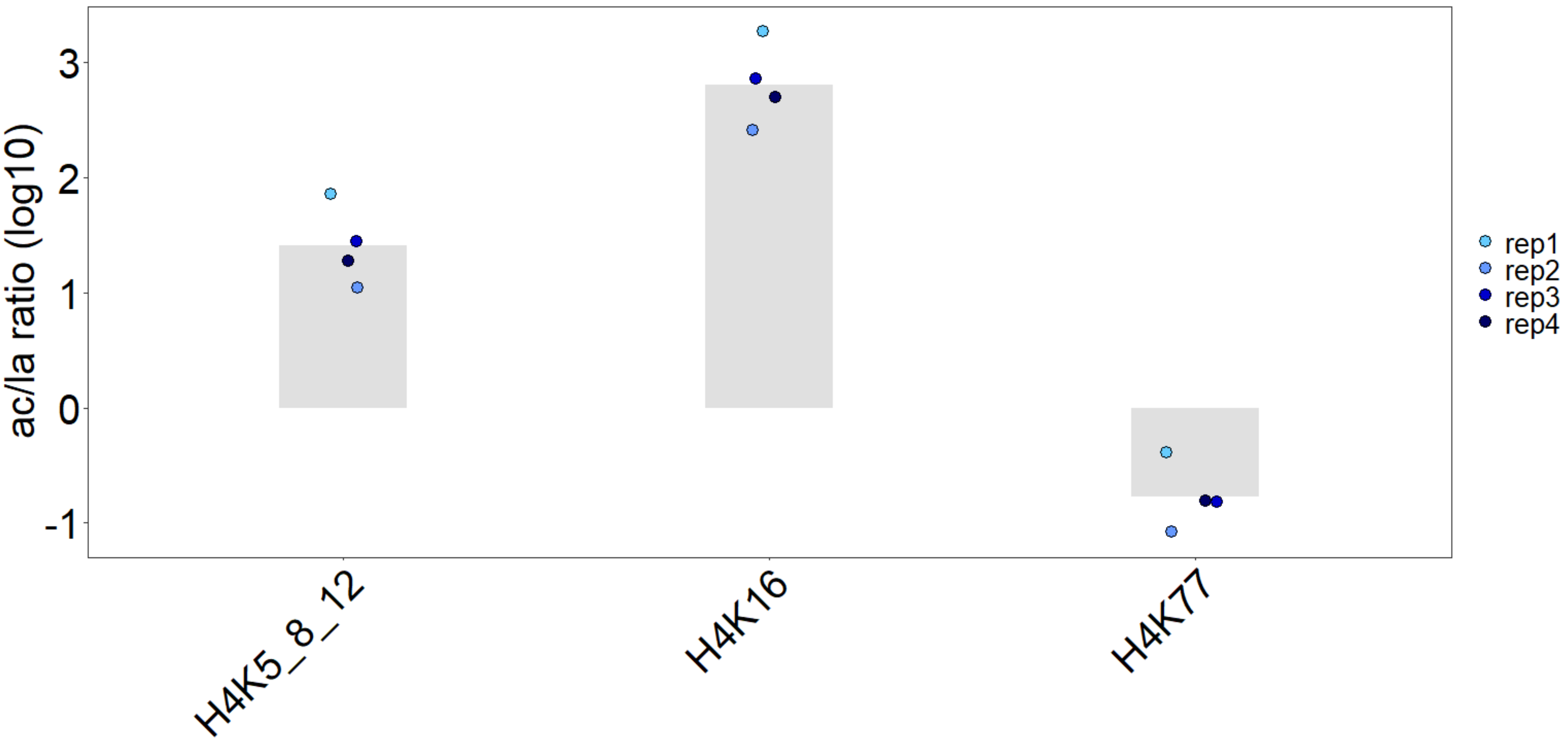

Section 9: Analysis of histones from spermatocytes and round spermatids

Gel obtained on histones extracted from spermatocytes (SC) and round spermatids (RS)

Samples were prepared in triplicate. Protein samples enriched in histones were loaded on a gel and bands corresponding to H3+H2B and H4 were cut to be processed by propionylation-digestion, before LC-MS/MS analysis.

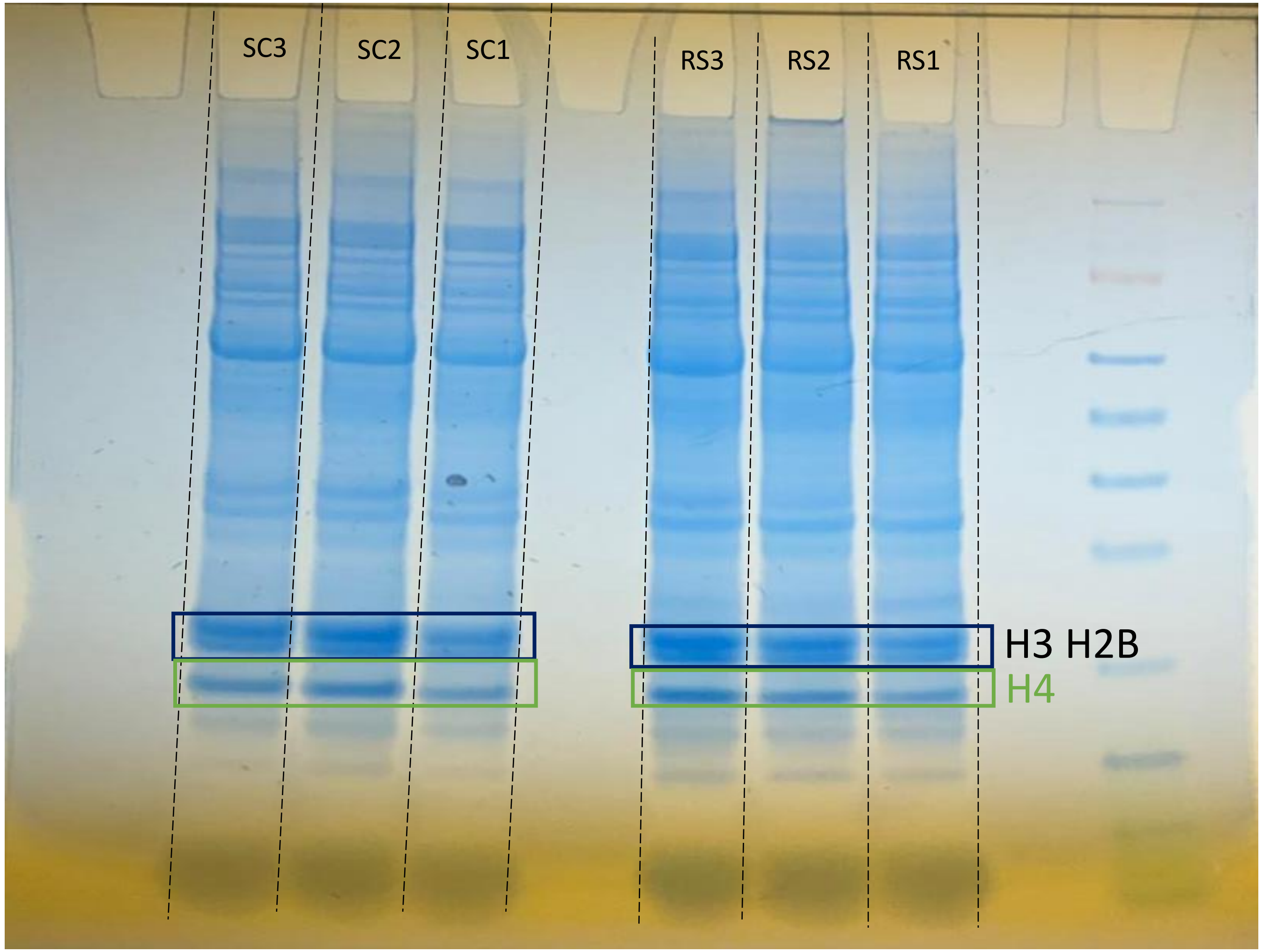

Estimation by PRM analysis of the relative abundance of L- and D-lactylation at H3.tK18 in spermatocytes (top panels) and round spermatids (bottom panels)

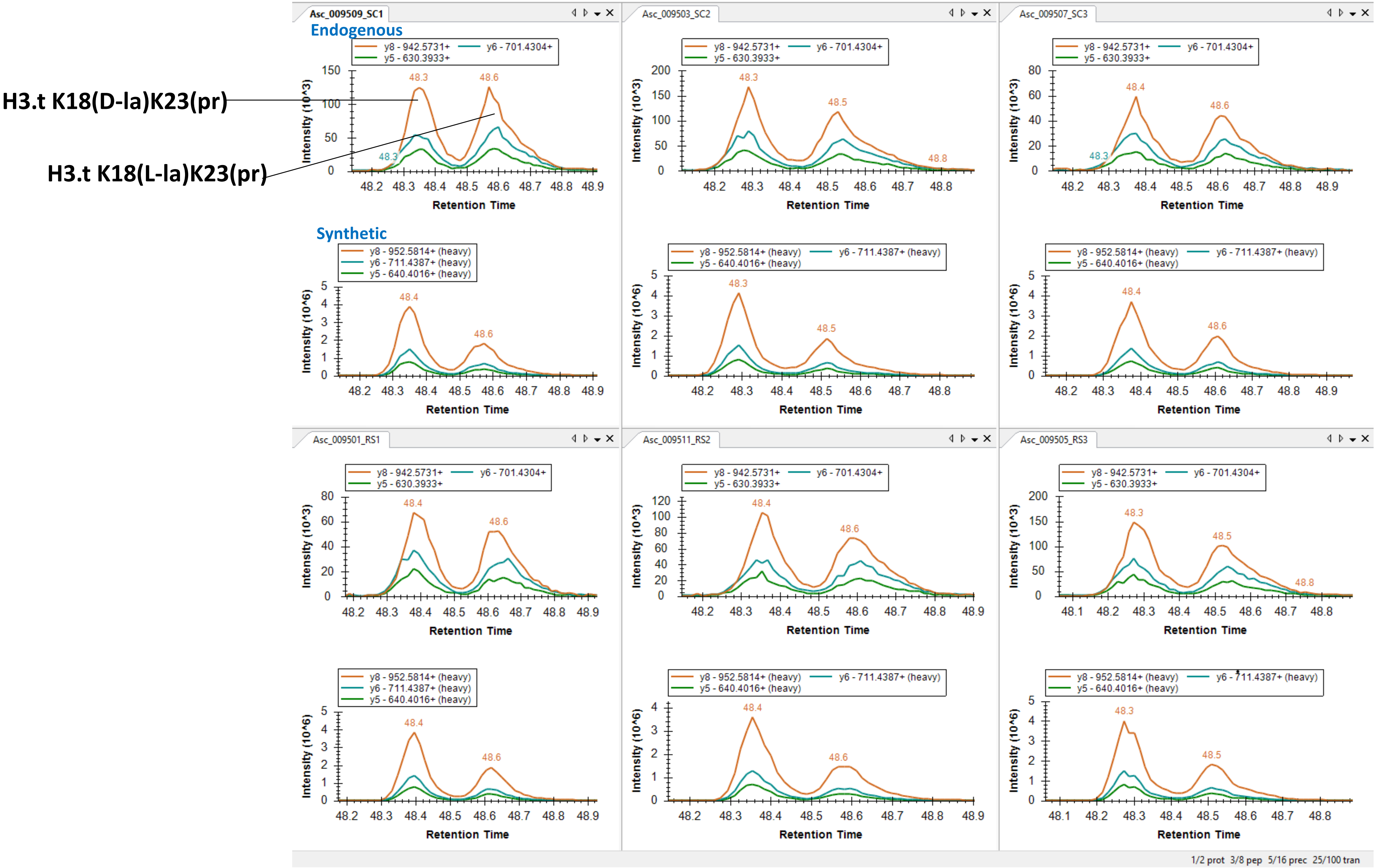

Estimation by PRM analysis of the relative abundance of L- and D-lactylation at H3.tK23 in spermatocytes (top panels) and round spermatids (bottom panels)

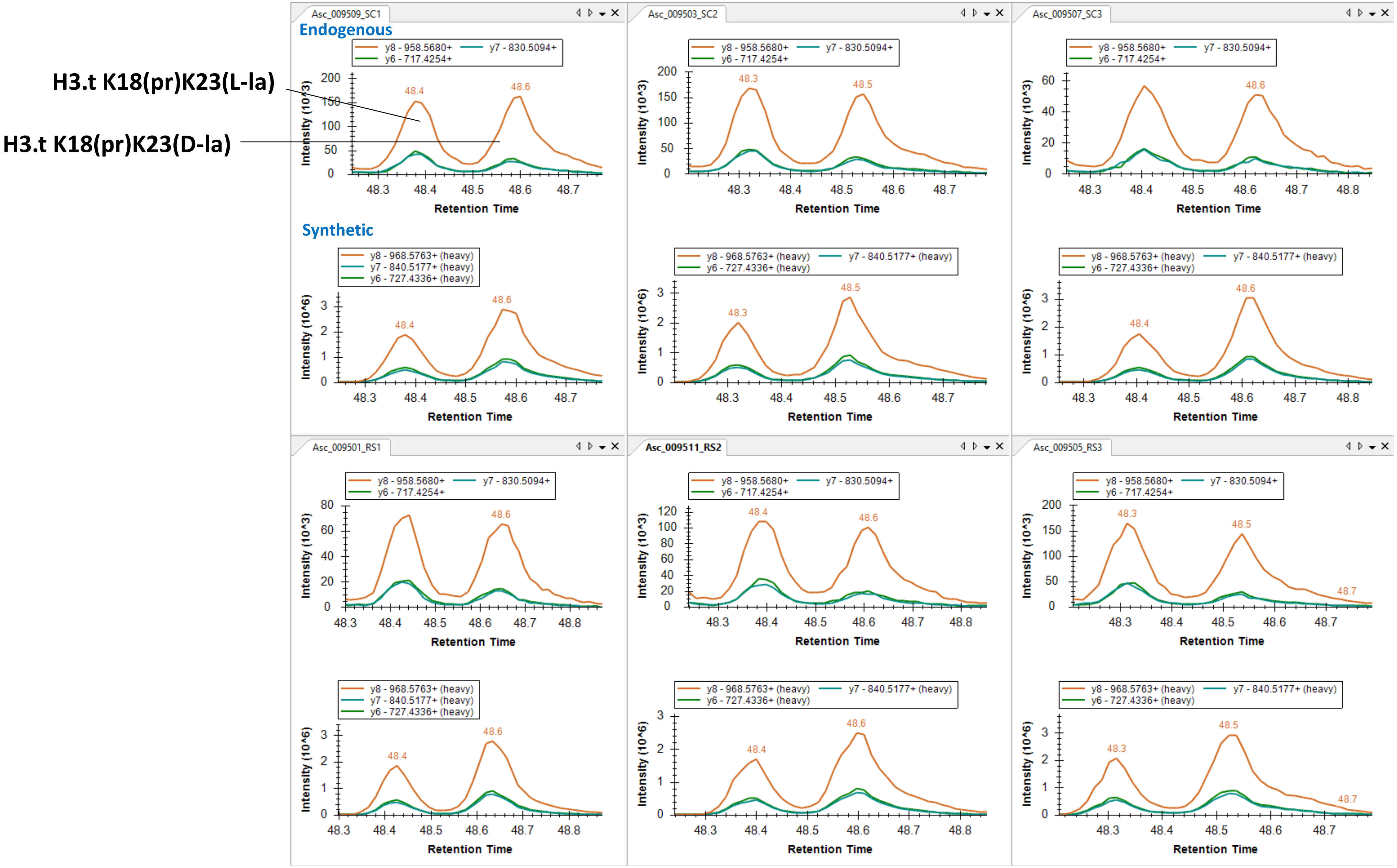

Estimation by PRM analysis of the relative abundance of L- and D-lactylation at H3.1K18 in spermatocytes (top panels) and round spermatids (bottom panels)

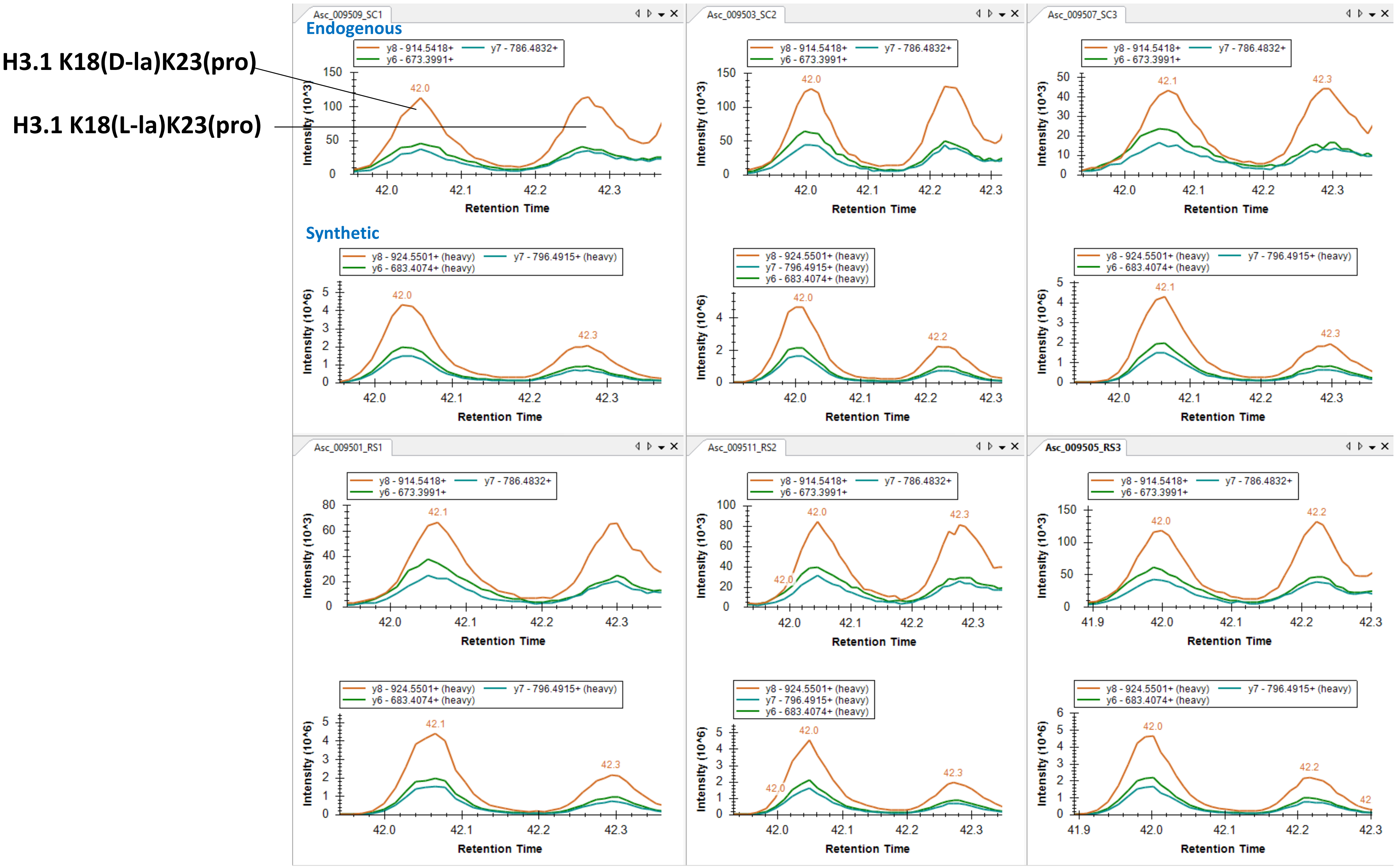

Estimation by PRM analysis of the relative abundance of L- and D-lactylation at H3.1K23 in spermatocytes (top panels) and round spermatids (bottom panels)

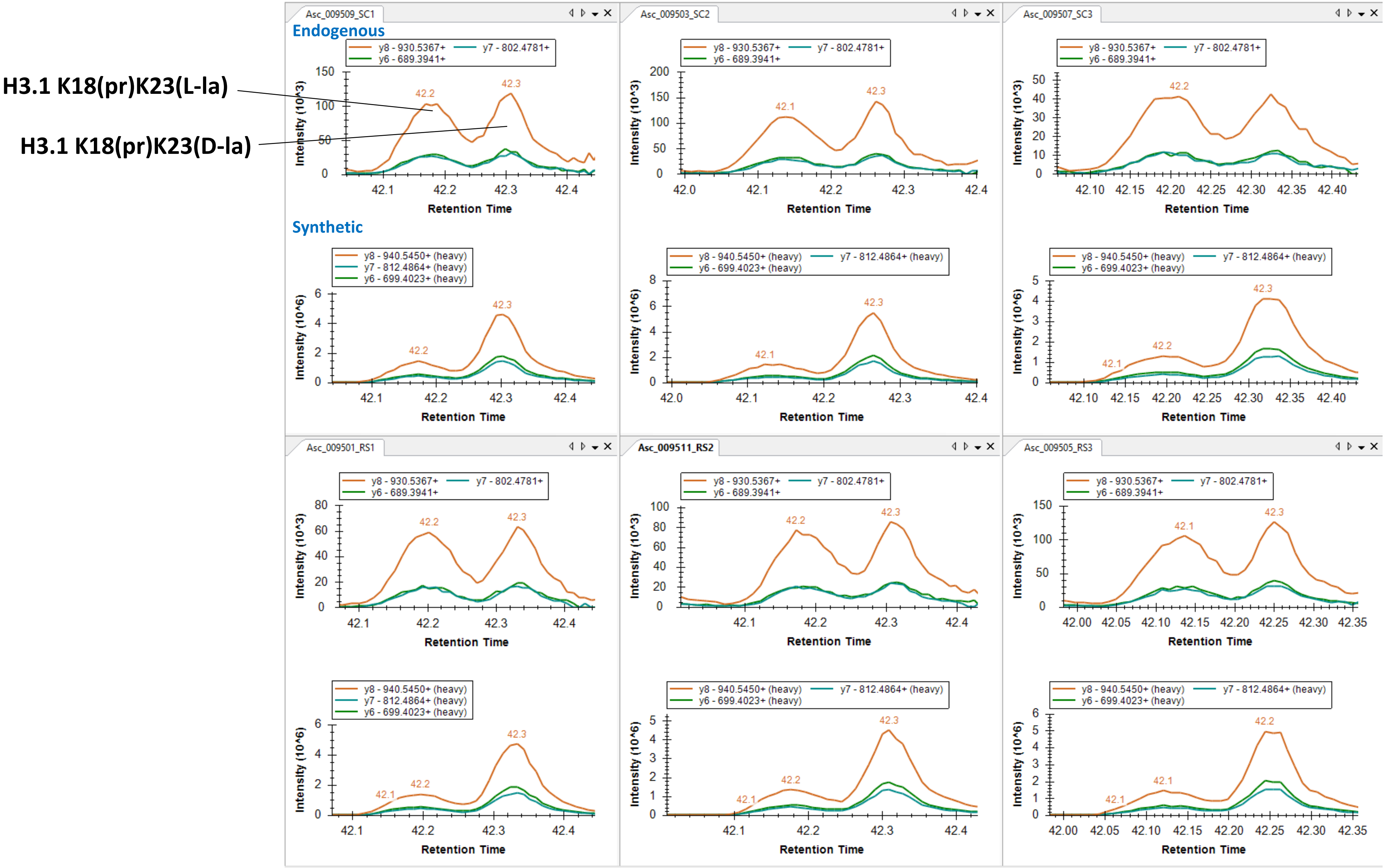

Section 10: Verification of the absence of the isomeric structure with lactyl, N-ε-(carboxyethyl)

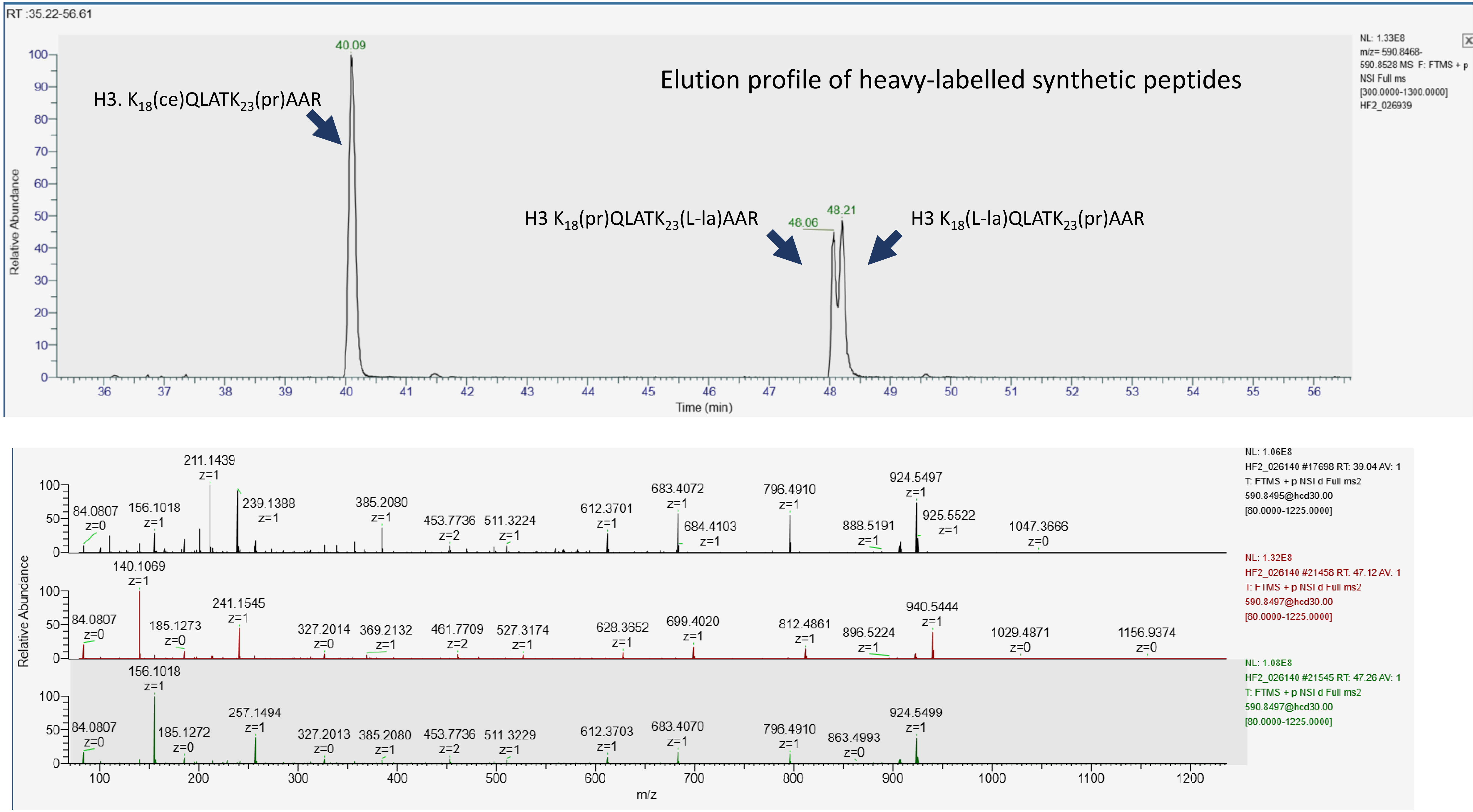

RT :35.22-56.61
Elution profile of heavy-labelled synthetic peptides
H3. K18(ce)QLATK23(pr)AAR
H3 K18(pr)QLATK23(L-la)AAR
H3 K18(L-la)QLATK23(pr)AAR
40.09
48.06
48.21
NL: 1.33E8
m/z= 590.8468-590.8528 MS F: FTMS + p NSI Full ms [300.0000-1300.0000]
HF2_026939
Relative Abundance
Time (min)
NL: 1.06E8
HF2_026140 #17698 RT: 39.04 AV: 1
T: FTMS + p NSI d Full ms2 590.8495@hcd30.00 [80.0000-1225.0000]
NL: 1.32E8
HF2_026140 #21458 RT: 47.12 AV: 1
T: FTMS + p NSI d Full ms2 590.8497@hcd30.00 [80.0000-1225.0000]
NL: 1.08E8
HF2_026140 #21545 RT: 47.26 AV: 1
T: FTMS + p NSI d Full ms2 590.8497@hcd30.00 [80.0000-1225.0000]
m/z

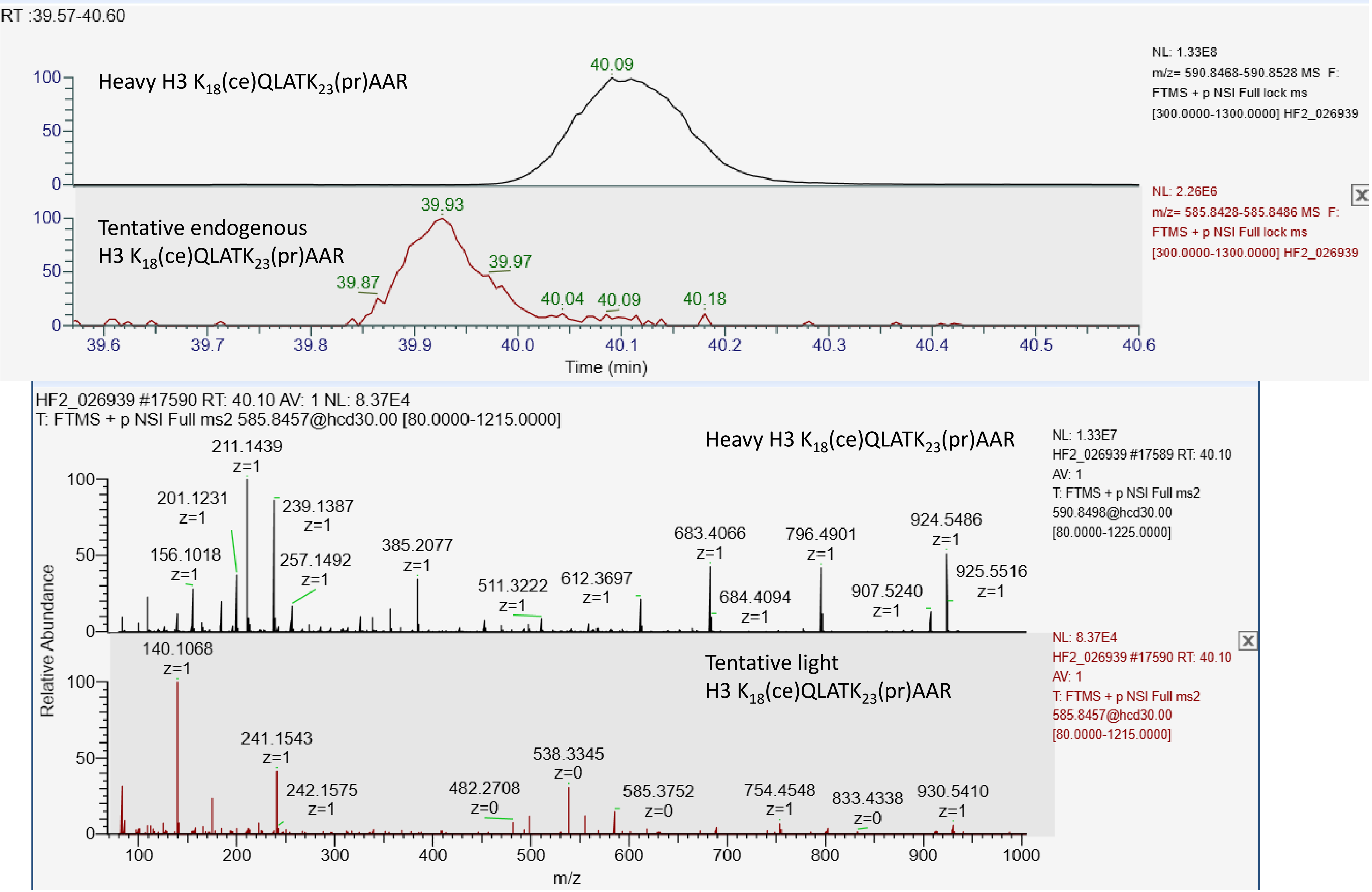
RT :39.57-40.60
Heavy H3 K18(ce)QLATK23(pr)AAR
NL: 1.33E8
m/z= 590.8468-590.8528 MS F: FTMS + p NSI Full lock ms [300.0000-1300.0000] HF2_026939
Tentative endogenous H3 K18(ce)QLATK23(pr)AAR
NL: 2.26E6
m/z= 585.8428-585.8486 MS F: FTMS + p NSI Full lock ms [300.0000-1300.0000] HF2_026939
40.09
39.93
39.87
39.97
40.04
40.09
40.18
Time (min)
HF2_026939 #17590 RT: 40.10 AV: 1 NL: 8.37E4
T: FTMS + p NSI Full ms2 585.8457@hcd30.00 [80.0000-1215.0000]
Heavy H3 K18(ce)QLATK23(pr)AAR
NL: 1.33E7
HF2_026939 #17589 RT: 40.10 AV: 1
T: FTMS + p NSI Full ms2 590.8498@hcd30.00 [80.0000-1225.0000]
211.1439 z=1
201.1231 z=1
239.1387 z=1
156.1018 z=1
257.1492 z=1
385.2077 z=1
511.3222 z=1
612.3697 z=1
683.4066 z=1
684.4094 z=1
796.4901 z=1
907.5240 z=1
924.5486 z=1
925.5516 z=1
Tentative light H3 K18(ce)QLATK23(pr)AAR
NL: 8.37E4
HF2_026939 #17590 RT: 40.10 AV: 1
T: FTMS + p NSI Full ms2 585.8457@hcd30.00 [80.0000-1215.0000]
140.1068 z=1
241.1543 z=1
242.1575 z=1
482.2708 z=0
538.3345 z=0
585.3752 z=0
754.4548 z=1
833.4338 z=0
930.5410 z=1
Relative Abundance
m/z
